# Variation in grain Zn concentration, and the grain ionome, in field-grown Indian wheat

**DOI:** 10.1371/journal.pone.0192026

**Published:** 2018-01-30

**Authors:** Jaswant Singh Khokhar, Sindhu Sareen, Bhudeva Singh Tyagi, Gyanendra Singh, Lolita Wilson, Ian P. King, Scott D. Young, Martin R. Broadley

**Affiliations:** 1 School of Biosciences, University of Nottingham, Sutton Bonington Campus, Loughborough, United Kingdom; 2 Indian Institute of Wheat & Barley Research, Karnal, (Haryana), India; National Institute of Plant Genome Research, INDIA

## Abstract

Wheat is an important dietary source of zinc (Zn) and other mineral elements in many countries. Dietary Zn deficiency is widespread, especially in developing countries, and breeding (genetic biofortification) through the HarvestPlus programme has recently started to deliver new wheat varieties to help alleviate this problem in South Asia. To better understand the potential of wheat to alleviate dietary Zn deficiency, this study aimed to characterise the baseline effects of genotype (G), site (E), and genotype by site interactions (GxE) on grain Zn concentration under a wide range of soil conditions in India. Field experiments were conducted on a diverse panel of 36 Indian-adapted wheat genotypes, grown on a range of soil types (pH range 4.5–9.5), in 2013–14 (five sites) and 2014–15 (six sites). Grain samples were analysed using inductively coupled plasma-mass spectrometry (ICP-MS). The mean grain Zn concentration of the genotypes ranged from 24.9–34.8 mg kg^-1^, averaged across site and year. Genotype and site effects were associated with 10% and 6% of the overall variation in grain Zn concentration, respectively. Whilst G x E interaction effects were evident across the panel, some genotypes had consistent rankings between sites and years. Grain Zn concentration correlated positively with grain concentrations of iron (Fe), sulphur (S), and eight other elements, but did not correlate negatively with grain yield, i.e. no yield dilution was observed. Despite a relatively small contribution of genotype to the overall variation in grain Zn concentration, due to experiments being conducted across many contrasting sites and two years, our data are consistent with reports that biofortifying wheat through breeding is likely to be effective at scale given that some genotypes performed consistently across diverse soil types. Notably, all soils in this study were probably Zn deficient and interactions between wheat genotypes and soil Zn availability/management (e.g. the use of Zn-containing fertilisers) need to be better-understood to improve Zn supply in food systems.

## Introduction

Zinc is an essential element for plants and people [1, 2, 3]. Approximately 20% percent of the world’s population is at risk of having insufficient Zn in their diets, based on food supply and composition, with much greater proportions at risk in sub-Saharan Africa and South Asia [4, 5, 6]. Dietary Zn deficiency is linked to diarrhoea, respiratory infections and stunting (low height to weight ratios) in children [7, 8]. Wheat (*Triticum aestivum* L.) is the third most widely consumed cereal crop by weight, after rice and maize, providing ~20% of total global energy and protein supply [9]. This figure rises to >50% of energy in some countries, for example, in India (www.dwr.in). Wheat is an important dietary source of Zn [10] and supplies ~50% Zn of daily needs in India [5] (http://harvestplus.org/where-we-work/india). Over the past 20 years, agronomic and breeding-based approaches have been developed to increase the Zn concentration of wheat grain [11, 12]. The most effective agronomic approaches to increasing wheat grain Zn concentrations involve foliar applications of ZnSO_4_ solution in the presence of adequate nitrogen [13]. This approach is likely to be cost-effective in terms of yield and health benefits but may require changes to current farming practices [14–18]. Breeding for increased grain Zn concentration is likely to be a more cost-effective approach in the longer term provided there is adequate plant-available Zn in the soil [16, 19–21].

The ‘HarvestPlus’ programme is developing and deploying new wheat varieties with higher grain Zn concentrations in South Asia [22]. The HarvestPlus target is to enhance the grain Zn concentration in locally-adapted wheat varieties by 8–12 mg kg^-1^, from a baseline of ~25 mg kg^-1^, without compromising grain yield or other grain quality attributes [23, 24]. Through collaboration with Banaras Hindu University (BHU), Uttar Pradesh (UP), India, and several private Indian seed companies including Sai Seeds, Nirmal Seeds, and Astha Beej, three varieties with increased grain Zn concentration have been multiplied and released in Eastern UP (North Eastern Plain Zone, NEPZ): Akshai (BHU-3), Abhay (Zinc Shakthi), and BHU-6 [25]. A high zinc wheat variety ‘Zincol-2016’ has been recommended for release and multiplication in Pakistan by the Pakistan Agriculture Research Council (PARC; http://www.harvestplus.org/node/1647). The wheat genotypes used in the present study have been selected to represent a wide diversity of crop types which span all of the agro-climatic wheat growing zones of India for good yield, yield components and quality traits. Most of these genotypes are used as reference lines within advanced variety trials for screening new wheat lines, including under saline/acid soils [26, 27].

It is likely that optimal grain Zn concentrations will ultimately be achieved using a combination of crop improvement and agronomy [28, 15, 16, 29, 30]. However, to date, little work has reported baseline relative contributions of genotype (G), environment (E) and G*E interactions on wheat grain Zn concentration under widely differing field conditions. Therefore, the aim of this study was to quantify baseline variation in grain Zn concentrations of field-grown wheat, using a diverse panel of Indian-adapted wheat genotypes grown on a wide range of soil types but without addition of Zn fertilisers. Simultaneously, the grain concentration of a wider range of elements was determined (NB, the mineral composition of biological tissues is typically referred to as the ‘ionome’ [31]).

## Material and methods

### Wheat genotypes, field sites and management

A panel of 36 elite wheat genotypes comprising *Triticum aestivum* L. (n = 34) and *Triticum durum* (n = 2) was selected for study under field conditions. These genotypes represent a diverse genetic background with adaptations to a range of climatic and soil environments. All genotypes and experimental conditions are described in Khokhar *et al*. [26], including data for yield and yield components. In brief, the panel was grown at six field sites in major wheat growing areas of India, during *rabi* (winter) seasons 2013–14 and 2014–15. The six sites were, (1) the Indian Institute of Wheat & Barley Research (IIWBR), Karnal (29.70° N; 76.99° E), Haryana (HR); (2) IIWBR, Hisar (29.18° N; 75.70° E) (HR); (3) Narendra Deva University of Agriculture and Technology (NDUA&T), Kumarganj (26.43° N; 82.17° E), Faizabad, Uttar Pradesh (UP), reclaimed site; (4) NDUA&T, Kumarganj (26.43° N; 82.17° E), Faizabad (UP), sodic site; (5) Uttar Banga Krishi Viswavidyalaya (UBKV), Regional Research Sub-Station (RRSS), Mathurapur (25.57° N; 87.10° E), Malda (WB) and (6) UBKV, Pundibari (26.32° N; 89.45° E), Cooch Behar, West Bengal (WB). Seeds were manually placed in four rows per plot at 25 cm spacing between rows. The plot length was 2.5 m. The plots were arranged in a simple lattice design (6x6) with two replicates according to standard IIWBR practices. The field experiments were conducted at research farms of IIWBR, Karnal; NDUA&T, Kumarganj and UBKV, Pundibari, with their research director’s permission and study received the permission from the Indian Council of Agricultural Research (ICAR) and Department of Biotechnology (DBT) under a collaborative project between India and University of Nottingham, UK; grant number BT/IN/UK/12/IS/2012.

### Determining plant-available soil Zn

Top- and sub-soil were sampled with a hand-auger during both growing seasons at all six sites. Five samples were taken at each experimental site at each depth from the vertices of a ‘W-pattern’. A single composite sample of 1 kg was retained for each soil depth. To determine plant-available soil Zn concentrations, air-dried soil samples were sieved to 2 mm and a 1.5 g subsample extracted with 10 mL of 0.01 M Ca(NO_3_)_2_ by shaking for 1 h on a rotary shaker. Soil suspensions were then centrifuged at 3300 rpm for 20 minutes and the supernatant liquid was filtered through 0.22 μm polyether sulfone (PES) syringe filters and acidified to a final concentration of 2% HNO_3_ (Primar Plus^™^, Fisher). Multi-elemental analyses was conducted on (technical) triplicate samples using inductively coupled plasma-mass spectrometry (ICP-MS), as described for the grain samples below. A total of 30 and 36 soil samples were analysed in 2013–14 and 2014–15, respectively; at Kumarganj-reclaimed and Kumarganj-sodic sites, soil samples were taken only from one depth, 0–30 cm, in 2013–14.

### Grain sampling and digestion

Grains of each plot from 5 sites (all sites except Pundibari) in 2013–14, and from plots on all 6 sites in 2014–15 were harvested at physiological maturity and sun-dried. A representative sub-sample of grain from each plot was carefully hand-cleaned by discarding broken grains and foreign material. In 2013–14, ~10 grains of each genotype were weighed and digested using a microwave system comprising a Multiwave 3000 platform with a 48-vessel MF50 rotor (Anton Paar Gmbh, Graz, Austria); digestion vessels were perfluoroalkoxy (PFA) tubes in polyethylethylketone (PEEK) pressure jackets (Anton Paar GmbH). Grains were digested in 2 mL 70% Trace Analysis Grade (TAG) HNO_3_, 1 mL Milli-Q water (18.2 MΩ cm; Fisher Scientific UK Ltd, Loughborough, UK), and 1 mL H_2_O_2_. Prior to digestion, the samples were soaked in this solution for 16 h at room temperature. In 2014–15, grain samples were crushed inside a paper bag, and subsamples (~0.200 g DW) were then used without the soaking step. The microwave settings were: power = 1400 W, temperature = 140°C, pressure = 2 MPa, time = 45 minutes. Two operational blanks were included in each digestion run. Duplicate samples of a certified reference material (CRM: Wheat flour SRM 1567b, NIST, Gaithersburg, MD, USA) were included approximately every fourth digestion run. A laboratory reference material (LRM), Paragon was used for each digestion run. Following digestion, each tube was made up to a final volume of 15 mL by adding 11 mL Milli-Q water, then transferred to a 25 mL universal tube (Sarstedt Ltd., Nümbrecht, Germany) and stored at room temperature. Samples were further diluted 1:5 with Milli-Q water into 13 ml tubes (Sarstedt Ltd.) prior to analysis.

### Zinc and multi-element analysis

The grain and plant-available soil concentrations of Zn and 30 other elements were determined by ICP-MS (Thermo Fisher Scientific iCAPQ, Thermo Fisher Scientific, Bremen, Germany). The other elements were Ag, Al, As, B, Ba, Be, Ca, Cd, Cr, Co, Cs, Cu, Fe, K, Li, Mg, Mn, Mo, Na, Ni, P, Pb, Rb, S, Se, Sr, Ti, Tl, U, and V. Three operational ICP-MS modes were used: (i) a helium collision-cell (He-cell) with kinetic energy discrimination to remove polyatomic interferences, (ii) standard mode (STD) in which the collision cell was evacuated, and (iii) a hydrogen collision-reaction cell (H_2_-cell). Samples were introduced from an auto sampler incorporating an ASXpress^™^ rapid uptake module (Cetac ASX-520, Teledyne Technologies Inc., Omaha, NE, USA) through a PEEK nebuliser (Burgener Mira Mist, Mississauga, Burgener Research Inc., Canada). Internal standards were introduced to the sample stream on a separate line via the ASXpress unit and included Sc (20 μg L^-1^), Rh (10 μg L^-1^), Ge (10 μg L^-1^) and Ir (5 μg L^-1^) in 2% TAG HNO3 (Fisher Scientific UK Ltd). External multi-element calibration standards (Claritas-PPT grade CLMS-2; SPEX Certiprep Inc., Metuchen, NJ, USA) included Ag, Al, As, B, Ba, Be, Cd, Ca, Co, Cr, Cs, Cu, Fe, K, Li, Mg, Mn, Mo, Na, Ni, P, Pb, Rb, S, Se, Sr, Ti, Tl (semi-quant), U, V and Zn, in the range 0–100 μg L^-1^ (0, 20, 40, 100 μg L^-1^). A bespoke external multi-element calibration solution (PlasmaCAL, SCP Science, Courtaboeuf, France) was used to create Ca, K, Mg and Na standards in the range 0–30 mg L^-1^. Boron, P and S calibration utilised in-house standard solutions (KH_2_PO_4_, K_2_SO_4_ and H_3_BO_3_). In-sample switching was used to measure B and P in STD mode, Se in H_2_-cell mode and all other elements in He-cell mode. Sample processing was undertaken using Qtegra^™^ software (Thermo Fisher Scientific) with external cross-calibration between pulse-counting and analogue detector modes when required. In total, 288 grain samples were analysed in 7 runs in 2013–14, and 432 grain samples in 12 runs in 2014–15, excluding blank, CRM and LRM samples. The Zn-specific recovery from CRMs was 96% in 2013–14 and 94% in 2014–15 compared with certified CRM values.

### Data analysis of grain Zn concentration and the grain ionome

For each data-point, an element-specific operational blank concentration (mean of each ICP-MS run) was subtracted. Data were then multiplied by initial sample volume, divided by the initial dry mass of material, and converted to mg element kg^-1^ dry grain material. Element-specific limits of detection (LODs) were reported as 3 times the standard deviation (SD) of the ten operational blank concentrations, assuming a notional starting dry weight of 0.4 g in 2013–14 and 0.2 g in 2014–15 (S1 Table). Out of 31 mineral elements, 15 (Ag, Al, B, Be, Cd, Co, Cr, Li, Na, Ni, Pb, Tl, Ti, U, V) were removed from further analysis because their mean grain concentration across all plots was less than the LOD (S2 Table). For the remaining elements, when an individual sample had a grain element concentration less than the LOD, actual values were replaced with 50% LOD. Grain element concentrations >5 standard deviation (SDs) greater than the global arithmetic mean for each element were also removed from the analysis as a precaution against using contaminated samples (15 and 31 data points were removed in 2013–14 and 2014–15, respectively). All data are provided in S3 Table.

### Statistical analyses

Variance components associated with grain concentration of Zn and 15 other grain mineral elements (As, Ba, Ca, Cs, Cu, Fe, K, Mg, Mn, Mo, P, Rb, S, Se, and Sr) were calculated. Analyses of Variance (ANOVA) and Least Significant Difference (LSD) tests were used to test for differences in mean grain elemental concentration between sites and genotypes. Broad-sense heritability (H^2^) was calculated from ANOVA for all the grain mineral elements across all sites as H^2^ = V_g_/V_p_ where V_g_ is the genotypic variance and V_p_ is the phenotypic variance. A genotype plus genotype by environment (site) interaction (GGE) analysis was performed to construct a biplot for grain Zn concentration to study the genotype and genotype by environment interaction effect, to explore the adaptation of genotypes to the specific sites. Differences between sites and genotypes for grain mineral composition traits were considered significant at P<0.01. Pearson correlation coefficients were calculated for the 16 grain mineral elements, along with grain yield, for each site in each year. All analyses were conducted using GenStat 17^th^ Edition (VSN International Ltd, Hemel Hempstead, UK).

## Results

This study reports the contribution of genotype and site factors to variation in grain Zn concentration, and the grain ionome, of 36 wheat genotypes in India. Primary data are provided in S4 Table.

### Variation in wheat grain Zn concentration and the grain ionome

The mean grain Zn concentration across all plots and years (n = 719) was 29.3 mg kg^-1^ and varied from 3.4 to 60.9 mg kg^-1^, across all plots (Table 1). Among the macronutrients, the mean grain concentration of Ca, K, Mg, P and S were 369, 4190, 1160, 3610 and 1490 mg kg^-1^, respectively and varied 20-fold for Ca, 14-fold for K, 15-fold for Mg, 189-fold for P and 17-fold for S. Among the micronutrients, the mean grain concentration of Cu, Fe, Mn and Mo across all plots were 4.3, 37.8, 37.5 and 0.74 mg kg^-1^, respectively (n = 719). Grain concentration varied 12-fold for Cu, 13-fold for Fe, 11-fold for Mn and 41-fold for Mo. Among trace elements, the mean grain As and Se concentrations were 0.02 and 0.16 mg kg^-1^, respectively, and varied from 0.003 to 0.089 for As and from 0.02 to 0.8 mg kg^-1^ for Se (Table 1). Cadmium (Cd) and lead (Pb) are trace element of interest in the context of Zn biofortification [32] because they are potentially toxic to humans [33–36]. In this study, the mean grain Cd and Pb concentrations across all plots were less than the LODs of 0.0341 and 0.0660 mg kg^-1^ for Cd and Pb, respectively, and were therefore not included in further data analyses.

**Table 1:** Grain concentrations of Zn and other mineral elements of Indian wheat. Data are in mg kg^-1^, summarised across all plots (n = 719). Grain yield and yield components data are summarised across all plots (n = 864).

Element	Mean	Median	SD	Range	LOD	Heritability
**Zn**	29.3	28.4	6.99	3.44–60.9	0.744	56.8
**As**	0.0186	0.0107	0.0183	0.003–0.089	0.00564	49.9
**Ba**	4.29	4.09	2.12	0.27–13.2	0.0493	89.2
**Ca**	369	362	86.1	36.9–726	17.6	83.3
**Cs**	0.0161	0.0100	0.0170	0.0004–0.093	0.00077	78.5
**Cu**	4.34	4.28	0.939	0.65–7.53	1.3	81.1
**Fe**	37.8	37.4	9.74	6.43–85.9	12.9	75.5
**K**	4190	4180	609	476–6520	11.9	80.4
**Mg**	1160	1130	216	140–2090	2.9	71.5
**Mn**	37.5	37.1	10.1	5.82–66.5	3.51	85.5
**Mo**	0.740	0.762	0.471	0.056–2.44	0.111	80.3
**P**	3610	3640	524	311–6030	7.2	51.1
**Rb**	8.55	7.91	6.34	0.156–26.7	0.00426	88.2
**S**	1490	1520	292	140–2270	280	73.6
**Se**	0.159	0.0998	0.138	0.015–0.824	0.0297	8.35
**Sr**	5.07	5.27	3.3	0.20–17.9	0.161	66.9
**Grain yield and yield components**						
**GYD**	3.83	3.38	1.68	0.28–8.43		33.4
**TGW**	38.1	38.3	6.44	14.9–63.8		63.1
**GWS**	1.76	1.72	0.48	0.218–3.41		62.2
**HI**	34.2	35.1	8.11	2.55–58.1		63.9
**PHT**	92.7	91.7	19.7	48.2–167		75.0
**DTM**	126	127	11.6	90–148		95.7

The mean grain yield (GYD) across all plots and years (n = 864) was 3.83 t ha^-1^ and varied from 0.28 to 8.43 t ha^-1^, across all plots (Table 1). The mean 1000 grain weight (TGW, g), grain weight per spike (GWS, g), harvest index (HI, %), plant height at maturity (PHT, cm) and days to maturity (DTM, days) were 38.1 g, 1.76 g, 34.2%, 92.7 cm and 126 days, respectively.

### Variance components associated with grain Zn concentration and the grain ionome

Genotype (G) and site terms (E) were associated with 10% and 6% of the variation in grain Zn concentration, respectively (Table 2). The G x E interaction term was associated with 20% of the variation in grain Zn concentration. The residual term (R) was associated with 64% of the variation in grain Zn concentration, and represents variation due to year, plot-to-plot variation between replicates, and technical/measurement errors.

**Table 2:** The contribution of G, E, G*E and residual factors to variation percentage (%) in grain Zn concentration and the grain ionome of a panel of 36 wheat genotypes grown at five sites in 2013–14 and at six sites in 2014–15 and in grain yield and yield components of a panel of 36 genotypes grown at six sites over two years.

Element	Variation %				P value		
	G	E	G*E	Residual	G	E	G*E
**Zn**	10	6	20	64	<0.001	<0.001	0.816
**Others**							
**As**	2	70	6	21	0.015	<0.001	0.908
**Ba**	27	39	11	22	<0.001	<0.001	<0.001
**Ca**	18	28	10	44	<0.001	<0.001	0.999
**Cs**	2	89	4	5	<0.001	<0.001	<0.001
**Cu**	14	14	11	61	<0.001	<0.001	1
**Fe**	9	19	9	63	<0.001	<0.001	1
**K**	18	18	15	49	<0.001	<0.001	0.841
**Mg**	11	36	12	41	<0.001	<0.001	0.93
**Mn**	11	55	9	25	<0.001	<0.001	0.591
**Mo**	4	79	4	13	<0.001	<0.001	0.798
**P**	12	8	21	59	<0.001	<0.001	0.446
**Rb**	2	87	3	8	<0.001	<0.001	0.213
**S**	8	12	9	71	0.019	<0.001	1
**Se**	1	67	5	27	0.960	<0.001	1
**Sr**	7	71	6	16	<0.001	<0.001	0.299
***d*.*f*.**	**35**	**5**	**175**	**503**			
**Grain yield and yield components**							
**GYD**	8	62	11	20	<0.001	<0.001	<0.001
**TGW**	16	37	17	30	<0.001	<0.001	<0.001
**GWS**	15	31	12	42	<0.001	<0.001	0.377
**HI**	16	21	13	50	<0.001	<0.001	0.545
**PHT**	23	55	5	17	<0.001	<0.001	0.443
**DTM**	3	75	3	19	<0.001	<0.001	1
**d.f.**	**35**	**5**	**175**	**648**			

Site (E) was typically associated with a greater proportion of the variation in the grain concentration of other minerals compared to Zn (Table 2). Among the macronutrients, 28, 18, 36, 8 and 12% of the variation in grain Ca, K, Mg, P and S concentration were associated with E; 18, 18, 11, 12 and 8% of the variation were associated with G, respectively. Among the micronutrients, E was associated with 14, 19, 55 and 79% of the variation in grain Cu, Fe, Mn and Mo concentration, respectively; 14, 9, 11 and 4% of the variation were associated with G, respectively. Among non-essential trace elements, 70 and 67% of the variation in grain As and Se concentration, respectively, were associated with E, with just 2 and 1% of the variation associated with G, respectively.

Most of the variation in yield and yield components was associated with site; 62, 55 and 75% of the variation in GYD, PHT and DTM, respectively (Table 2).

### Differences in grain Zn concentration and the grain ionome between genotypes

**Fig 1:**
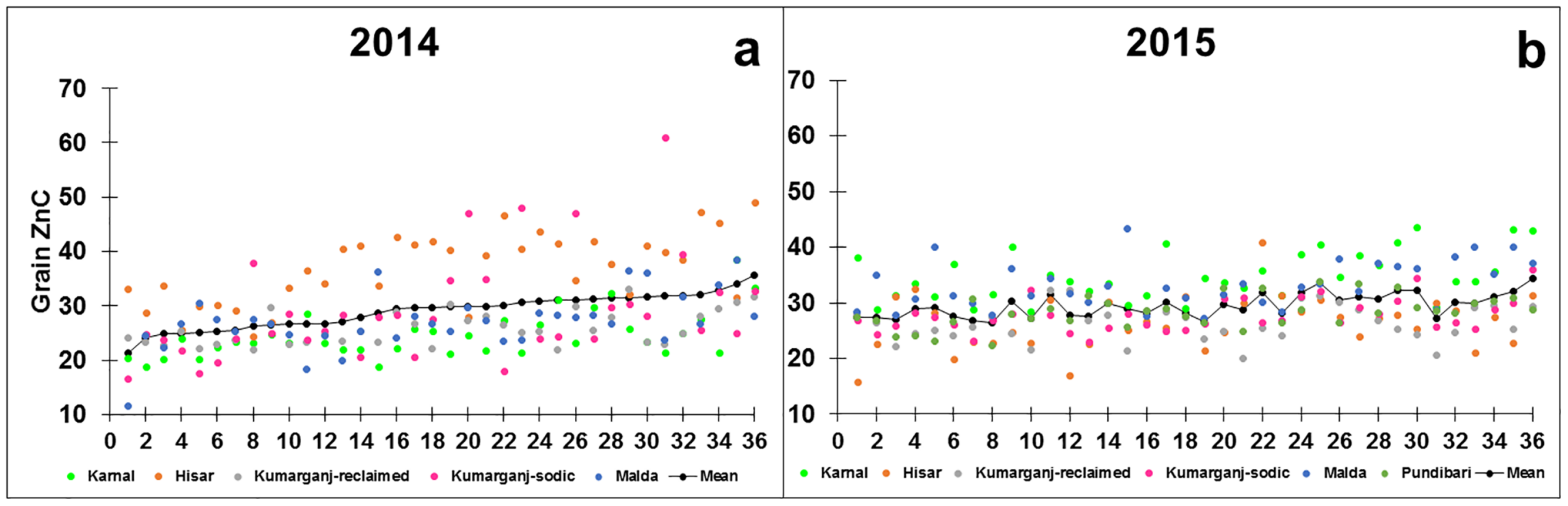
Grain Zn concentration in a panel of 36 genotypes, averaged across five sites in 2013–14 and six sites in 2014–15. Data represent the means of two replicate plots per genotype at Karnal, Hisar and Malda, and one replicate per genotype at Kumarganj-reclaimed and Kumarganj-sodic sites in 2013–14, and the means of two replicate plots of each genotype at all six sites in 2014–15. Genotypes 1–36 are labelled in the same order in both years on the x-axis: 1) HW 2044; 2) HD 2932; (3) RW 3684; (4) WH 1021; (5) HD 2967; (6) DBW 46; (7) KRL 1–4; (8) GW 322; (9) NW 4092; (10) GW 322; (11) NW 4092; (12) PDW 314; (13) RAJ 4229; (14) MACS 6222; (15) DPW 621–50; (16) WH 1105; (17) HI 1563; (18) KRL 210; (19) DBW 71; (20) NW 1067; (21) NW 4018; (22) DBW 14; (23) KRL 213; (24) HI 8498; (25) BH 1146; (26) DBW 51; (27) KRL 19; (28) UP 262; (29) DBW 17; (30) K 0307; (31) HD 2009; (32) HD 2733; (33) RAJ 4238; (34) DBW 39; (35) KRL 3–4; (36) Kharchia 65.

Grain Zn concentration differed between genotypes in both 2013–14 and 2014–15 (P<0.001). The mean grain Zn concentration of 36 genotypes, averaged across sites, varied from 21.4 to 35.6 mg kg^-1^ in 2013–14 (Fig 1a) and from 26.5 to 34.3 mg kg^-1^ in 2014–15 (Fig 1b). The Kharchia 65 genotype had the greatest grain Zn concentration in both years. The HW 2044 and GW 322 genotypes had the smallest grain Zn concentration in 2013–14 and in 2014–15, respectively.

Among the macronutrients and micronutrients, the grain concentrations of Ca, Mg, S, Fe, Cu and Mn differed between genotypes in both 2013–14 and 2014–15 (P<0.001). Among the non-essential trace elements, the grain As and Se concentration did not differ significantly between genotypes in either year.

The mean grain Ca concentration of 36 genotypes, averaged across sites, varied from 285.2 to 437 mg kg^-1^ in 2013–14 and from 317 to 453 mg kg^-1^ in 2014–15. The KRL 3–4 and NW 1067 genotypes had the greatest grain Ca concentration in 2013–14 and in 2014–15, respectively. The CBW 38 and NW 4018 genotypes had the smallest grain Ca concentration in 2013–14 and in 2014–15, respectively. The mean grain Mg concentration of 36 genotypes, averaged across sites, varied from 911 to 1330 mg kg^-1^ in 2013–14 and from 1050 to 1380 mg kg^-1^ in 2014–15. The KRL 3–4 genotype had the greatest grain Mg concentration in both years. The HW 2044 and KRL 213 genotypes had the smallest grain Mg concentration in 2013–14 and in 2014–15, respectively. The mean grain S concentration of 36 genotypes, averaged across sites, varied from 1080 to 1570 mg kg^-1^ in 2013–14 and from 1480 to 1870 mg kg^-1^ in 2014–15. The KRL 3–4 and BH 1146 genotypes had the greatest grain S concentration in 2013–14 and in 2014–15, respectively. The HW 2044 and GW 322 genotypes had the smallest grain S concentration in 2013–14 and in 2014–15, respectively.

Among the micronutrients, the mean grain Cu concentration of 36 genotypes, averaged across sites, varied from 3.1 to 4.9 mg kg^-1^ in 2013–14 and from 3.8 to 5.3 mg kg^-1^ in 2014–15. The KRL 19 and HI 1563 genotypes had the greatest grain Cu concentration in 2013–14 and in 2014–15, respectively. The WH 1021 and DBW 46 genotypes had the smallest grain Cu concentration in 2013–14 and in 2014–15, respectively. The mean grain Fe concentration of 36 genotypes, averaged across sites, varied from 26.7 to 42.9 mg kg^-1^ in 2013–14 and from 34.6 to 47.6 mg kg^-1^ in 2014–15. The Kharchia 65 and KRL 3–4 genotypes had the greatest grain Fe concentration in 2013–14 and in 2014–15, respectively. The HW 2044 and PDW 314 genotypes had the smallest grain Fe concentration in 2013–14 and in 2014–15, respectively. The mean grain Mn concentration of 36 genotypes, averaged across sites, varied from 25.0 to 43.1 mg kg^-1^ in 2013–14 and from 29.0 to 47.8 mg kg^-1^ in 2014–15. The BH 1146 and KRL 210 genotypes had the greatest grain Mn concentration in 2013–14 and in 2014–15, respectively. The PDW 314 genotype had the smallest grain Mn concentration in both years.

**Fig 2:**
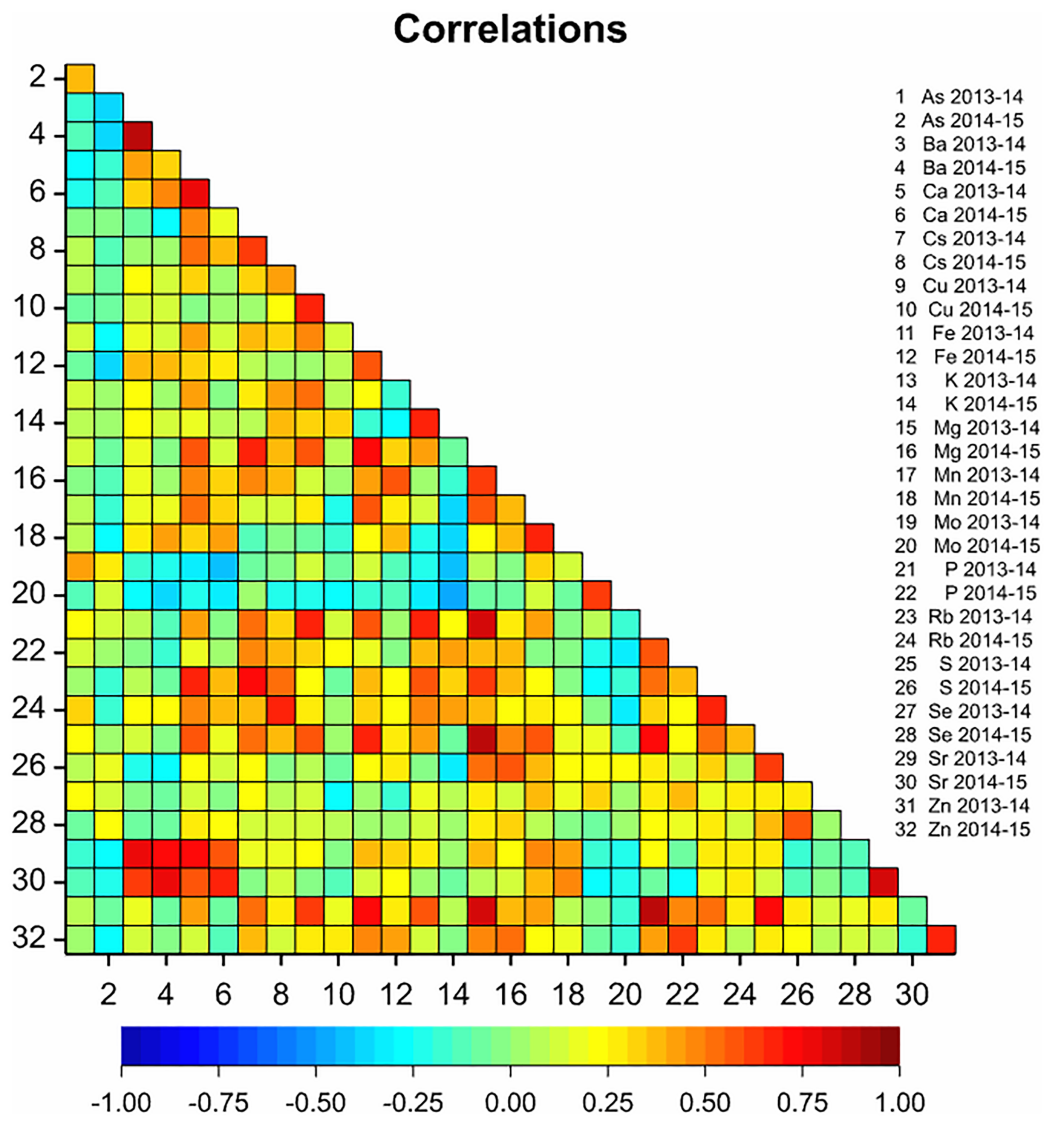
The correlation coefficients in grain ionome of a panel of 36 genotypes between 2013–14 and 2014–15. Data represent the means of two replicate plots per genotype at Karnal, Hisar and Malda, and one replicate per genotype at Kumarganj-reclaimed and Kumarganj-sodic sites in 2013–14, and the means of two replicate plots of each genotype at all six sites in 2014–15. Colour represents strength of correlation from strongly negative (dark blue) to strongly positive (dark red).

The mean grain Zn concentration of 36 genotypes showed a positive relationship between 2013–14 and 2014–15 (r = 0.65, P<0.001; Fig 2). Within sites, the mean grain Zn concentration of 36 genotypes showed positive relationships between 2013–14 and 2014–15 at Karnal (r = 0.64; P<0.001), Hisar (r = 0.36; P<0.05) and Malda (r = 0.63; P<0.001).

The mean grain Ca, Mg, S, Cu, Fe and Mn concentrations of 36 genotypes showed positive relationships between 2013–14 and 2014–15 (r between 0.61 and 0.77; P<0.001; Fig 2). The mean grain As concentration of 36 genotypes showed a positive relationship between 2013–14 and 2014–15 (r = 0.37, P<0.05). However, the mean grain Se concentration of 36 genotypes did not correlate between 2013–14 and 2014–15 (Fig 2).

### Differences in grain Zn concentration and the grain ionome between sites

**Fig 3:**
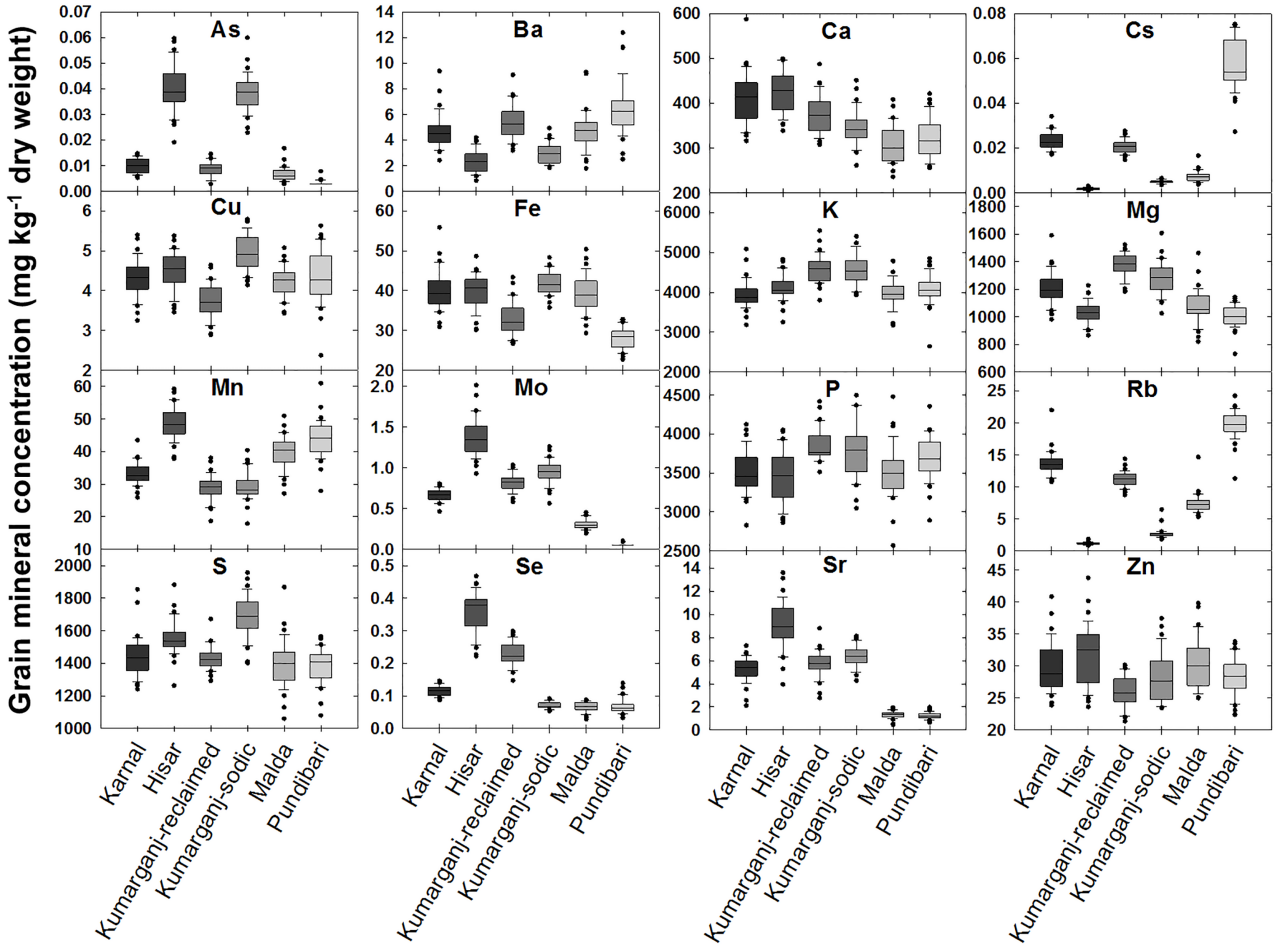
Grain element concentrations of 36 genotypes of wheat at six sites over two years. Data represent the means of two replicate plots per genotype at Karnal, Hisar, Malda and one replicate plot per genotype at Kumarganj-reclaimed and Kumarganj-sodic sites in 2013–14 and two replicate plots per genotype at all six sites in 2014–15. Boxes represent the two mid-quartiles with the median drawn; whiskers are the 95% confidence limits; circles are the outliers.

Grain Zn concentration differed between sites (P<0.001) (Fig 3). The mean grain Zn concentration varied from 26.1 (Kumarganj-reclaimed) to 31.7 (Hisar) mg kg^-1^, averaged across 36 genotypes and years (LSD = 1.74) (Fig 3).

Among the macronutrients, grain Ca, Mg, S concentrations differed between sites. The mean grain Ca concentration varied from 309 (Malda) to 425 (Hisar) mg kg^-1^, averaged across 36 genotypes and years (LSD = 17.77). The mean grain Mg concentration varied from 1000 (Pundibari) to 1370 (Kumarganj-reclaimed) mg kg^-1^ (LSD = 43.3). The mean grain S concentration varied from 1380 (Pundibari) to 1690 (Kumarganj-sodic) mg kg^-1^ (LSD = 76.8).

Among the micronutrients, the mean grain Cu concentration varied from 3.7 (Kumarganj-reclaimed) to 5.0 (Kumarganj-sodic) mg kg^-1^, averaged across 36 genotypes and years (LSD = 0.23). The mean grain Fe concentration varied from 28.1 (Pundibari) to 41.9 (Kumarganj-sodic) mg kg^-1^ (LSD = 2.41). The mean grain Mn concentration varied from 28.9 (Kumarganj-reclaimed) to 48.7 (Hisar) mg kg^-1^ (LSD = 1.59). Among the non-essential trace elements, the mean grain As concentration varied from 0.003 (Pundibari) to 0.040 (Hisar) mg kg^-1^, averaged across 36 genotypes and years (LSD = 0.003). The mean grain Se concentration varied from 0.07 (Malda) to 0.36 (Hisar) mg kg^-1^ (LSD = 0.02) (Fig 3).

**Fig 4:**
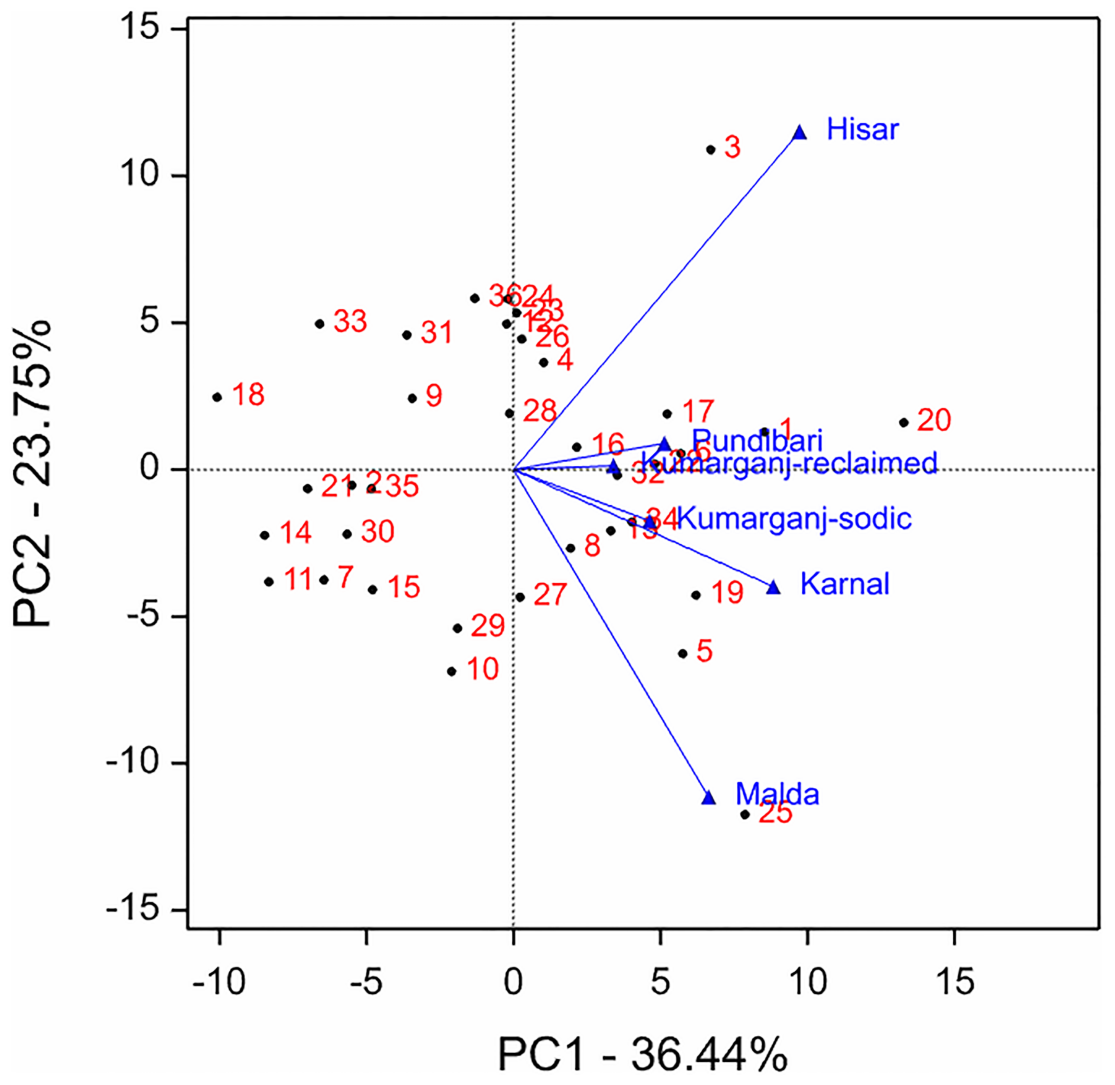
GGE biplot for grain Zn concentration of 36 genotypes evaluated at 6 sites over two years. Genotypes 1–36 are as: 1 (BH 1146); 2 (CBW 28); 3 (DBW 14); 4 (DBW 16); 5 (DBW 17); 6 (DBW 39); 7 (DBW 46); 8 (DBW 51); 9 (DBW 71); 10 (DPW 621–50); 11 (GW 322); 12 (HD 2009); 13 (HD 2733); 14 (HD 2932); 15 (HD 2967); 16 (HI 1563); 17 (HI 8498); 18 (HW 2044); 19 (K 0307); 20 (Kharchia 65); 21 (KRL 1–4); 22 (KRL 19); 23 (KRL 210); 24 (KRL 213); 25 (KRL 3–4); 26 (MACS 6222); 27 (NW 1067); 28 (NW 4018); 29 (NW 4092); 30 (PDW 314); 31 (Raj 4229); 32 (Raj 4238); 33 (RW 3684); 34 (UP 262); 35 (WH 1021); 36 (WH 1105).

The genotype and genotype by environment interaction (GGE) effects were visualised using a biplot (Fig 4). The biplot explained 60.2% of total variation with the contribution of 36.4% from principal component-1 (PC-1) and 23.8% from principal component-2 (PC-2). The genotypes 1 (BH 1146) and 20 (Kharchia 65) which have the PC1 value >0 and PC2 value near to zero being considered the more stable and highest Zn containing genotypes across all the sites. Genotypes 6 (DBW 39), 16 (HI 1563), 22 (KRL 19) and 32 (Raj 4238) were also among the more stable genotypes for grain Zn concentration. Genotypes which are closest to specific sites indicates that they are likely to be better suited to that particular site for high grain Zn concentration. For example, genotype 3 (DBW 14) at Hisar; 5 (DBW 17) and 19 (K 0307) at Karnal; 25 (KRL 3–4) at Malda; 1 (BH 1146), 6 (DBW 39) and 22 (KRL 19) at Kumarganj-reclaimed and Pundibari, and 8 (DBW 51) and 34 (Up 262) at Kumarganj-sodic site, showed higher grain Zn concentration (Fig 4).

### Relationship of grain Zn concentration with grain ionome and grain yield

**Fig 5:**
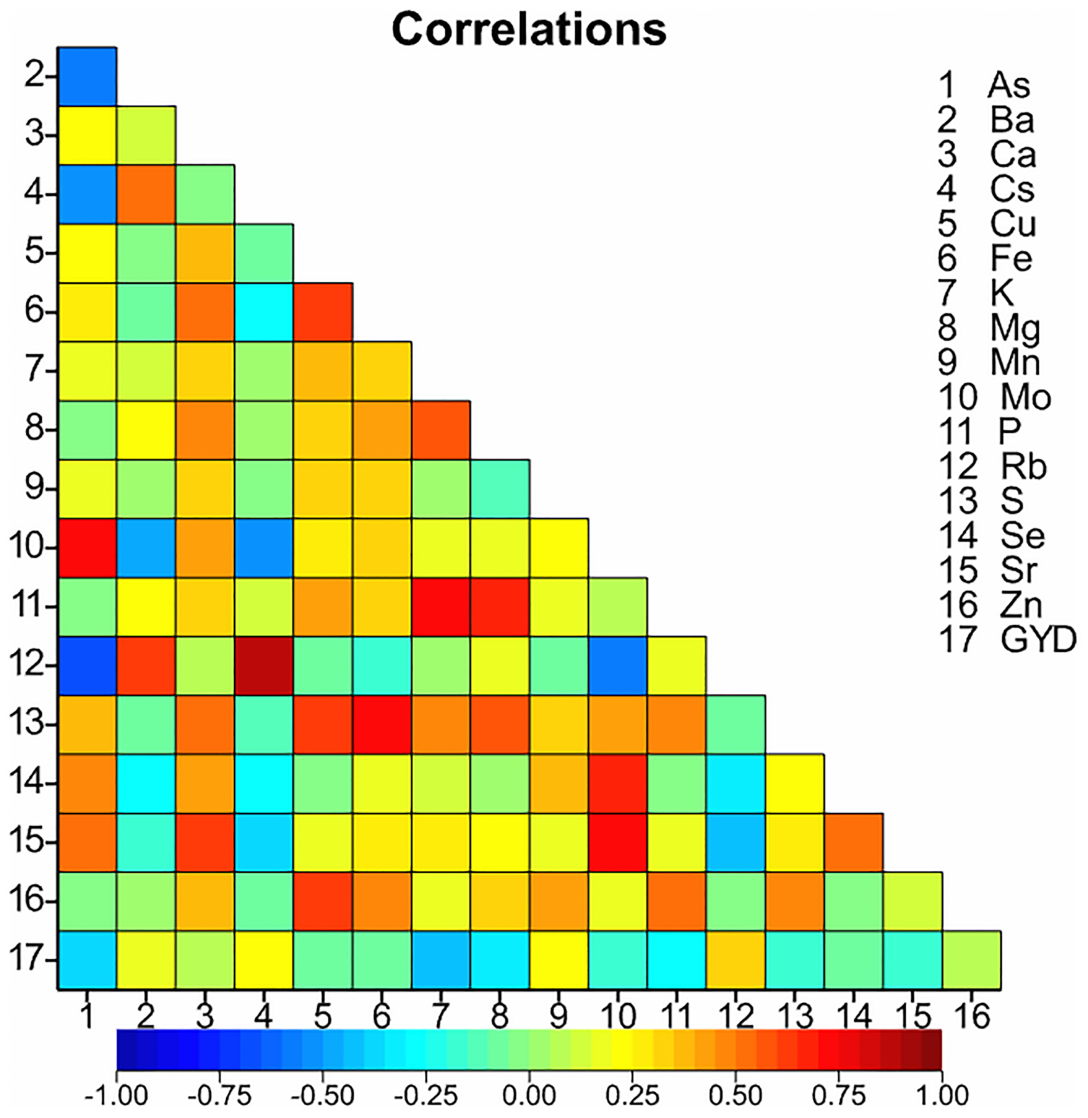
The relationship of grain Zn concentration (mg kg^-1^) with GYD (t ha^-1^) and other grain mineral elements in a panel of 36 wheat genotypes grown at five sites in 2013–14 and six sites in 2014–15. Data are means of two replicate per plot at Karnal, Hisar and Malda and one replicate at Kumarganj-reclaimed and Kumarganj-sodic sites in 2013–14 and two replicate per plot at six sites in 2004–15 (n = 719). Colour represents strength of correlation from strongly negative (dark blue) to strongly positive (dark red).

There was no negative correlation between grain Zn concentration and grain yield (GYD), rather a weakly positive correlation (r = 0.08; P = 0.03) (Fig 5). The grain Zn concentration showed positive significant relationships with grain Ca, Cu, Fe, K, Mg, Mn, Mo, P, S and Se (P<0.001) (Fig 5). However, grain Zn concentration did not show any relationships with grain As, Ba, Cs, Rb or Se concentrations. The grain Zn concentration showed stronger relationships with grain Cu, Fe, Mn, P and S (r = 0.61, 0.46, 0.43, 0.53, 0.46, respectively) than with grain Ca, K, Mg, Mo and Sr (r = 0.36, 0.20, 0.33, 0.18, 0.15, respectively) concentrations (Fig 5).

### Variation in grain Zn concentration due to variation in plant-available soil Zn

In the present study, plant-available soil Zn concentrations were reported in top and sub-surface soils at six sites in 2013–14 and 2014–15 based on the use of a non-conventional extract of Ca(NO_3_)_2_ [37, 38] (Table 3). Plant available soil Zn concentration varied from 0.04–1.10 mg kg^-1^ in 0–15 cm and from 0.03–2.14 mg kg^-1^ in 15–30 cm soil depths in 2013–14 while in 2014–15, it varied from 0.03–0.22 mg kg^-1^ in 0–15 cm and from 0.003–0.06 mg kg^-1^ in 15–30 cm soil depths, at six sites. All sites showed very low concentrations of immediately plant available Zn in the soil in both years except at Pundibari in 2013–14.

**Table 3:** Plant available soil Zn (Ca(NO_3_)_2_-extractable) and grain Zn concentration at six sites in 2013–14 and 2014–15. Soil Zn data are means of three replicate soil samples of each depth at each site. Grain Zn data are mean of two replicate at Karnal, Hisar, Malda and one replicate at Kumarganj-reclaimed and Kumarganj-sodic sites in 2013–14 and two replicates at all six sites in 2014–15.

Sites	[Zn]_soil_ (mg kg^-1^) at two soil depths (cm)				[Zinc]_grain_	
	2013–14		2014–15			
	0–15	15–30	0–15	15–30	2013–14	2014–15
**Karnal**	0.04	0.03	0.10	0.06	24.6	34.9
**Hisar**	0.33	0.03	0.22	0.04	36.8	26.4
**Kumarganj-reclaimed**	0.03*	0.03*	0.03	0.03	25.8	26.3
**Kumarganj-sodic**	0.03*	0.03*	0.17	0.03	29.1	27.8
**Malda**	0.28	0.46	0.03	0.03	27.1	33.3
**Pundibari**	1.10	2.14	0.21	0.05	-	28.3

* Zn concentration at 0–30 cm of soil depth.

At Karnal, soil available Zn concentration was greater in 2014–15 than in 2013–14 at both soil depths and also showed greater grain Zn concentration in 2014–15 than in 2013–14. Contrastingly, at Malda, the soil available Zn concentration was greater in 2013–14 than 2014–15 in both the soil depths, but, grain Zn concentration was greater in 2014–15 than in 2013–14 (Table 3).

## Discussion

Wheat is an important dietary source of Zn for the Indian population and there is scope to increase grain Zn concentrations in wheat through breeding to help alleviate dietary Zn deficiency. In the present study, the grain Zn concentration of 36 wheat genotypes varied from 26–32 mg kg^-1^. Our results are consistent with Zhao *et al*. [39] who reported grain Zn concentration from 14 to 35 mg kg^-1^ in 150 field grown bread wheat lines. A similar range of grain Zn concentration were reported among 66 advanced wheat genotypes (20–39 mg kg^-1^) selected from Central Asia Breeding Programs by Morgounov *et al*. [40], and grown in Kazakhstan, Kyrgyzstan and Tajikistan. Among 40 bread wheat lines from CIMMYT breeding programmes grown at nine locations in South Asia and Mexico, grain Zn concentration from 29–40 mg kg^-1^ [23]. A summary of grain Zn concentrations was recently published at global scale in field grown wheat and reported grain Zn concentration from 20–31 mg kg^-1^, showed a difference of 10 to 20 mg kg^-1^ from the biofortification target of 40 mg kg^-1^ for human diet [41].

Because experiments were conducted at a wide range of soil conditions, and over two seasons, genotype (G) accounted for a relatively small proportion of the total variation in grain Zn concentration. This is not surprising given that grain Zn concentration is a complex trait, under the control of many genes that can be modified under different environments. Joshi *et al*. [42], Velu *et al*. [23, 43] and Swamy *et al*. 2016 [44] also reported a significant effect of different environments on variation in grain Zn concentration. However, some genotypes still had a consistent ranking across sites and years for grain Zn concentration. For example, Kharchia 65 and BH 1146 genotypes showed comparatively greater grain Zn concentration across six sites. Genotype, Kharchia 65 is a salt tolerant genotype [45] and BH 1146 is an aluminium tolerant genotype [46]. Therefore, Kharchia 65 and BH 1146 might be potential new sources of background variation for crossing with new Zn-biofortified wheat varieties, including for saline/sodic and acidic soil areas. In addition to Kharchia 65 and BH 1146, several genotypes achieved more than 31 mg kg^-1^ of grain Zn concentration at different locations, which is equivalent to 50% of the HarvestPlus target of 12 mg kg^-1^ above a notional baseline grain Zn concentration of 25 mg kg^-1^. Given that these lines already have implicitly good grain yield and other quality attributes for India, such lines could also be useful for crossing with high-Zn lines derived from the HarvestPlus, to support site-specific approaches as adopted previously for yield and yield component traits [26].

The high-Zn wheat varieties were developed by HarvestPlus using the high Zn containing accessions of synthetic wheats, spelt wheats and, further crossing with well adapted high-yielding hexaploid wheat varieties. Synthetic wheat lines were developed by crossing of *Triticum durum* and high-Zn containing wild accessions of tetraploid *Triticum dicoccon* with *Aegilops squarrosa* [22]. The *T*. *durum* based synthetic hexaploid wheat was developed by crossing of *T*. *durum* wheat cultivar with *Aegilops squarrosa*, a D genome donor of wheat, and further selections have developed a high-Zn variety, the Zinc Shakti (CROC1_/AE.SQUARROSA (210)//INQALAB91*2/KUKUNA/3/PBW343*2/KUKUNA) for North-Eastern Plain Zones of India and showed 14 mg kg^-1^ greater grain Zn concentration than a notional baseline grain Zn concentration of 25 mg kg^-1^. The *T*. *dicoccon* based synthetic hexaploid wheat was developed by crossing of a high Zn containing accession of wild *T*. *dicoccon* with *Aegilops squarrosa* and further selections have developed the WB02 and HPBW-01 (T.DICOCCON, CI9309/AE.SQUARROSA (409)//MUTUS/3/2*MUTUS) wheat varieties for North-Western Plain Zones of India and showed 7 mg kg ^-1^ greater grain Zn concentration than the notional baseline grain Zn concentration of 25 mg kg^-1^. Similarly, Zincol-2016 has been released for the Pakistan and developed by crossing of a well-adapted NARC2011 wheat variety with a high Zn containing accession of *T*. *spelta* and showed 9 mg kg ^-1^ greater grain Zn concentration than the notional baseline grain Zn concentration of 25 mg kg^-1^ [22].

There was no evidence of a yield dilution effect on grain Zn concentration in this study. Our result is therefore consistent with findings of Welch and Graham [47] and Joshi *et al*. [42], who reported no trade- off between grain Zn and grain yield concentration in wheat. This observation supports the argument that locally adapted, high yielding, genotypes have potential value for developing Zn-biofortified varieties for a wide range of soil conditions than reported previously. In contrast, other studies reported negative correlation of grain Zn with grain yield concentration in wheat [48, 49]. Oury *et al*. [49], reported lower grain Zn concentrations in high yielding genotypes which may have been due to yield dilution. It is important to continue to monitor grain Zn concentration in the wider context of yield and yield component traits in breeding programmes, especially given that grain yields in the region are relatively low.

Variation in plant-available Zn in soils is likely to represent a large proportion of environmental sources of variation in grain Zn concentration [11, 50, 51, 52]. Most of the soils in the present study were Zn deficient, albeit based on the use of a Ca(NO_3_)_2_ soil extraction to assess Zn-availability rather than the standard diethylene triamine pentaacetic acid (DTPA) method. Karami *et al*. [50], Joshi *et al*. [42] and Velu *et al*. [43] reported lower grain Zn concentrations in wheat genotypes when grown under Zn-deficient soil conditions. Soil Zn concentrations can be increased through the use of fertilisers [11, 15, 16]. Zinc can be applied with granular fertilisers to soil or as a foliar spray. Application of Zn fertiliser in granular form has produced increases in both grain yield and grain Zn concentration. However, application of Zn fertiliser as a foliar spray can reduce the amount Zn fertiliser needed in granular form and cereals are more responsive in terms of increase in grain Zn concentration, albeit at higher application costs. It is possible to reduce the cost of application by combining foliar application of Zn with pesticide applications [6, 17, 53]. Based on a wide survey of farmers, application of basal Zn fertilisers increased grain yield in wheat in Indo-Gangetic area of Sindh and Punjab Provinces of Pakistan [16]. A novel aspect of this study is that the same panel was grown in an extremely diverse range of hostile soils that will have complex trace element geochemistry. For example, sodic soils at the Kumarganj site had a pH value of 9.5, which is much higher than in the arable soils routinely studied in crop trials. At such sites, it is likely that reclamation using addition of gypsum (calcium sulphate, CaSO_4_.2H_2_O), and farm yard manure (FYM) coupled with measures to offset soil structural damage and impeded drainage would be needed to optimise grain yields and quality.

Increases in grain Zn concentration may also help to increase the grain Ca, Cu, Fe, K, Mg, Mn, Mo, P, and S concentrations given the observed positive correlation of grain Zn with these elements. The ionome data of high-Zn wheat varieties released by HarvestPlus in India and Pakistan is not available because the standard HarvestPlus method to measure the whole grain Zn concentration is X-ray fluorescence (XRF) [54]. However, our results are consistent with those of Pandey *et al*. [55], who reported a positive relationship between grain Zn concentrations and Ca, Cu, Fe, K, Mg, P and S concentrations in 150 bread wheat lines collected from India and Turkey. Many researchers also reported relationships between grain Zn and Fe [56, 23, 57], P [58, 59] and S [60]. Ozkan *et al*. [61] reported a major QTL on chromosome 5 which increased the grain Fe, Zn, Cu and Mn concentration in *Triticum monococcum* wheat genotypes. Velu *et al*. [57] reported co-localisation of grain Zn and Fe QTLs on 2B chromosomes which may help to simultaneous improvement of both the micronutrients. Similarly, McDonald and Mousavvi [60] showed that slightly increase in sulphur concentration increased the grain Zn concentration. It may be due to increase in S containing amino acid methionine, which further increased the level of phytosiderophores and nicotinamide, involved in uptake and translocation process of zinc. However, P is an important component of phytic acid (PA) which affects the bioavailability of Zn [59]. Therefore, the role of PA should be considered concomitantly in breeding programs intended to increase the grain Zn concentration in wheat genotypes.

Taken together, the results from this study show considerable potential for genetic improvement for Indian wheat genotypes. Some elite varieties (e.g. Kharchia 65) could be useful sources of variation that can be used at a wide range of sites to cross with new Zn-biofortified lines. This study also reveals the wealth of information that can be gleaned from studying the wider grain ionome across sites with a wide range of conditions, so that both breeding and spatial approaches (e.g. site-specific crop selection, fertiliser-use, etc.) to improving diet quality can deployed most effectively.

## Supporting information

S1 Table Element specific limits of detection (LOD) for concentration of 31 elements measured in grain of 36 Indian wheat genotypes by ICP-MS. (PDF)

S2 Table The grain mineral elements whose grain concentrations were below the limit of detection (LOD) values (highlighted in red). Elements highlighted in red excluded from data analyses.
(PDF)

S3 Table Grain mineral concentrations (mg kg^-1^) of 31 elements of 36 wheat genotypes grown at 5 sites in 2013–14 and at 6 sites in 2014–15 in India. Elements highlighted in red excluded from analysis (values below than LOD) and values higher than mean+(5*SD) (highlighted in yellow) are removed from data before analysis.
(PDF)

S4 Table Data of grain mineral composistion traits (mg kg^-1^) of 36 wheat genotypes grown at five sites in 2013–14 and at six sites in 2014–15 in India. Data below limit of detection (LOD) have been replaced by half LOD values and values above than mean+(5*SD) have been removed.
(PDF)
