# Supplementary material for: Variation in grain Zn concentration, and the grain ionome, in field-grown Indian wheat
Source: PLoS One. 2018 Jan 30;13(1):e0192026. doi: 10.1371/journal.pone.0192026 (PMC5790267; doi:10.1371/journal.pone.0192026)
Supplement: S1 Table — (PDF) [file pone.0192026.s001.pdf]

Supplementary Table 1. Element specific limits of detection (LOD) for concentrations of 31elements measured in grain of 36 *Indian wheat genotypes* by ICP-MS.

	Elements	LOD (mg kg^{-1})
	**B**	2.69
	**Na**	99.1
	**Mg**	2.90
	**P**	7.20
	**S**	280
	**K**	11.9
	**Ca**	17.6
	**Ti**	105
	**Li**	0.00989
	**Be**	0.000784
	**Al**	13.99
	**V**	0.0113
	**Cr**	2.14935
	**Mn**	3.51
	**Fe**	12.9
	**Co**	0.0555
	**Ni**	1.03
	**Cu**	1.30
	**Zn**	0.744
	**As**	0.00564
	**Se**	0.0297
	**Rb**	0.00426
	**Sr**	0.161
	**Mo**	0.111
	**Ag**	0.0127
	**Cd**	0.0341
	**Cs**	0.000770
	**Ba**	0.0493
	**Tl**	0.00105
	**Pb**	0.0660
	**U**	0.00524
