# Supplementary material for: Variation in grain Zn concentration, and the grain ionome, in field-grown Indian wheat
Source: PLoS One. 2018 Jan 30;13(1):e0192026. doi: 10.1371/journal.pone.0192026 (PMC5790267; doi:10.1371/journal.pone.0192026)
Supplement: S2 Table — Elements highlighted in red excluded from data analyses. (PDF) [file pone.0192026.s002.pdf]

Supplementary Table 2. The grain mineral elements whose mean grain concentrations were below the limit of detection (LOD) values. Elements highlighted in red excluded from analyses.

Elements	n	Mean concentration (Mg/Kg)	Median concentration (Mg/Kg)	Minimum concentration (Mg/Kg)	Maximun concentration (mg/kg)	SD	LOD
Ag	715	0.00764	0.00634	0.00634	0.0400	0.00392	0.0127
Al	717	7.94	7.00	7.00	39.4	3.74	13.99
As	719	0.0186	0.0107	0.00282	0.0893	0.0183	0.00564
B	717	1.46	1.35	1.35	8.62	0.687	2.69
Ba	719	4.29	4.10	0.273	13.2	2.12	0.0493
Be	717	0.000396	0.000392	0.000392	0.000935	0.0000429	0.000784
Ca	719	369	361.9	36.9	726	86.1	17.6
Cd	717	0.0287	0.0171	0.0171	0.131	0.0184	0.0341
Co	718	0.0312	0.0277	0.0277	0.129	0.0144	0.0555
Cr	718	1.08	1.07	1.07	2.930	0.111	2.14935
Cs	719	0.0161	0.0100	0.000385	0.0933	0.0170	0.000770
Cu	718	4.34	4.28	0.651	7.93	0.939	1.30
Fe	719	37.8	37.4	6.43	85.9	9.74	12.9
K	719	4190	4175	476	6519	609	11.9
Li	715	0.0137	0.00494	0.00494	0.0630	0.0112	0.00989
Mg	719	1162	1132	140	2086	216	2.90
Mn	719	37.5	37.1	5.82	66.5	10.1	3.51
Mo	719	0.740	0.762	0.056	2.44	0.471	0.111
Na	713	56.6	49.5	49.5	273	28.7	99.1
Ni	717	0.534	0.517	0.517	2.13	0.144	1.03
P	719	3611	3640	311	6026	524	7.20
Pb	717	0.0374	0.0330	0.0330	0.278	0.0235	0.0660
Rb	719	8.54	7.91	0.156	26.7	6.34	0.00426
S	719	1486	1515	140	2271	292	280
Se	719	0.159	0.0998	0.0148	0.824	0.138	0.0297
Sr	719	5.06	5.27	0.201	18.0	3.30	0.161
Ti	710	58.3	52.6	52.6	287	29.4	105
Tl	719	0.000523	0.000523	0.000523	0.000523	0.0000	0.00105
U	713	0.00438	0.00262	0.00262	0.0476	0.00584	0.00524
V	717	0.00906	0.00565	0.00565	0.0501	0.00644	0.0113
Zn	719	29.3	28.4	3.44	60.9	7.00	0.744
