# Supplementary material for: Variation in grain Zn concentration, and the grain ionome, in field-grown Indian wheat
Source: PLoS One. 2018 Jan 30;13(1):e0192026. doi: 10.1371/journal.pone.0192026 (PMC5790267; doi:10.1371/journal.pone.0192026)
Supplement: S3 Table — Elements highlighted in red excluded from analysis (values below than LOD) and values higher than mean+(5*SD) (highlighted in yellow) are removed from data before analysis. (PDF) [file pone.0192026.s003.pdf]

Supplementary Table 3. Grain mineral concentrations (mg/kg) of 31 elements of 36 wheat genotypes grown at 5 sites in 2013/14 and at 6 sites in 2014/15 in India. Elements highlighted in red excluded from analysis (values below than LOD) and values higher than mean+5*SD (highlighted in yellow) are removed from data before analysis.

Samples	Year	Year1	Year2	Site_Name	Site2014	site2015	Site Ranking	ZONE	Soil	HOSTILE_NOT	SOIL_pH	Replication	Genotype	Genotype Rank	Species	Plot_No	TGW	GYD_t/ha
1	2014	2014		KARNAL	KARNAL		1	NWPZ	NORMAL	NOT_HOSTILE	7.5	1	WH_1105	36	Triticum_aestivum	1	38.35	8.016
2	2014	2014		KARNAL	KARNAL		1	NWPZ	NORMAL	NOT_HOSTILE	7.5	1	NW_4092	29	Triticum_aestivum	2	36.46	6.44
3	2014	2014		KARNAL	KARNAL		1	NWPZ	NORMAL	NOT_HOSTILE	7.5	1	DBW_16	4	Triticum_aestivum	3	38.64	5.44
4	2014	2014		KARNAL	KARNAL		1	NWPZ	NORMAL	NOT_HOSTILE	7.5	1	NW_4018	28	Triticum_aestivum	4	33.52	6.372
5	2014	2014		KARNAL	KARNAL		1	NWPZ	NORMAL	NOT_HOSTILE	7.5	1	HD_2733	13	Triticum_aestivum	5	32.15	3.88
6	2014	2014		KARNAL	KARNAL		1	NWPZ	NORMAL	NOT_HOSTILE	7.5	1	DBW_39	6	Triticum_aestivum	6	41.35	7.52
7	2014	2014		KARNAL	KARNAL		1	NWPZ	NORMAL	NOT_HOSTILE	7.5	1	CBW_38	2	Triticum_aestivum	7	40.83	7.208
8	2014	2014		KARNAL	KARNAL		1	NWPZ	NORMAL	NOT_HOSTILE	7.5	1	NW_1067	27	Triticum_aestivum	8	33.57	5.32
9	2014	2014		KARNAL	KARNAL		1	NWPZ	NORMAL	NOT_HOSTILE	7.5	1	DBW_51	8	Triticum_aestivum	9	44.18	6.64
10	2014	2014		KARNAL	KARNAL		1	NWPZ	NORMAL	NOT_HOSTILE	7.5	1	KRL_19	22	Triticum_aestivum	10	28.69	3.86
11	2014	2014		KARNAL	KARNAL		1	NWPZ	NORMAL	NOT_HOSTILE	7.5	1	HI_1563	16	Triticum_aestivum	11	39.50	5.776
12	2014	2014		KARNAL	KARNAL		1	NWPZ	NORMAL	NOT_HOSTILE	7.5	1	KHARCHIA_65	20	Triticum_aestivum	12	34.76	1.36
13	2014	2014		KARNAL	KARNAL		1	NWPZ	NORMAL	NOT_HOSTILE	7.5	1	HW_2044	18	Triticum_aestivum	13	45.53	6.12
14	2014	2014		KARNAL	KARNAL		1	NWPZ	NORMAL	NOT_HOSTILE	7.5	1	DBW_46	7	Triticum_aestivum	14	44.26	6.12
15	2014	2014		KARNAL	KARNAL		1	NWPZ	NORMAL	NOT_HOSTILE	7.5	1	RAJ_4238	32	Triticum_aestivum	15	40.23	5.332
16	2014	2014		KARNAL	KARNAL		1	NWPZ	NORMAL	NOT_HOSTILE	7.5	1	RAJ_4229	31	Triticum_aestivum	16	43.48	5.92
17	2014	2014		KARNAL	KARNAL		1	NWPZ	NORMAL	NOT_HOSTILE	7.5	1	HI_8498	17	Triticum_turgidum	17	50.67	5.88
18	2014	2014		KARNAL	KARNAL		1	NWPZ	NORMAL	NOT_HOSTILE	7.5	1	KRL_1-4	21	Triticum_aestivum	18	39.02	6.08
19	2014	2014		KARNAL	KARNAL		1	NWPZ	NORMAL	NOT_HOSTILE	7.5	1	UP_262	34	Triticum_aestivum	19	38.07	4.32
20	2014	2014		KARNAL	KARNAL		1	NWPZ	NORMAL	NOT_HOSTILE	7.5	1	DPW_621-50	10	Triticum_aestivum	20	42.26	7.32
21	2014	2014		KARNAL	KARNAL		1	NWPZ	NORMAL	NOT_HOSTILE	7.5	1	KRL_213	24	Triticum_aestivum	21	33.70	5.864
22	2014	2014		KARNAL	KARNAL		1	NWPZ	NORMAL	NOT_HOSTILE	7.5	1	KRL_3-4	25	Triticum_aestivum	22	24.74	0.564
23	2014	2014		KARNAL	KARNAL		1	NWPZ	NORMAL	NOT_HOSTILE	7.5	1	HD_2967	15	Triticum_aestivum	23	42.92	6.72
24	2014	2014		KARNAL	KARNAL		1	NWPZ	NORMAL	NOT_HOSTILE	7.5	1	GW_322	11	Triticum_aestivum	24	41.24	5.668
25	2014	2014		KARNAL	KARNAL		1	NWPZ	NORMAL	NOT_HOSTILE	7.5	1	WH_1021	35	Triticum_aestivum	25	35.57	6.66
26	2014	2014		KARNAL	KARNAL		1	NWPZ	NORMAL	NOT_HOSTILE	7.5	1	HD_2932	14	Triticum_aestivum	26	35.92	4.808
[illegible]																		

Samples	B	Na	Mg	P	S	K	Ca	Ti	Li	Be	Al	V	Cr	Mn	Fe	Co	Ni
1	1.346226465	49.53407069	1175.43316	3385.658732	1247.314941	3562.390314	411.3237397	52.57059352	0.004943965	0.000391782	6.995184247	0.005652759	1.074675385	30.25917787	30.86099462	0.027737159	0.516801721
2	1.346226465	49.53407069	1128.571971	3070.286587	1327.611289	3549.260194	393.6351614	52.57059352	0.004943965	0.000391782	6.995184247	0.011711584	1.074675385	28.43342802	29.16197862	0.027737159	0.516801721
3	1.346226465	49.53407069	1130.968993	3095.299577	1155.635427	3714.209126	320.9045098	52.57059352	0.004943965	0.000391782	6.995184247	0.005652759	1.074675385	25.50411317	33.7911061	0.027737159	0.516801721
4	1.346226465	49.53407069	1019.811466	3035.768996	1181.685196	3597.712139	346.1703157	52.57059352	0.004943965	0.000391782	6.995184247	0.005652759	1.074675385	29.16014962	29.09682393	0.027737159	0.516801721
5	1.346226465	49.53407069	1141.16439	3382.799196	1204.606685	3522.221314	407.5533075	52.57059352	0.004943965	0.000391782	6.995184247	0.005652759	1.074675385	27.58646818	26.22012739	0.027737159	0.516801721
6	1.346226465	49.53407069	986.1999559	2884.535118	1099.406274	3286.044382	296.809891	52.57059352	0.010313704	0.000391782	6.995184247	0.005652759	1.074675385	27.87994358	30.99653844	0.027737159	0.516801721
7	1.346226465	49.53407069	1152.468623	3352.013697	1238.453228	3736.073138	322.8551253	52.57059352	0.004943965	0.000391782	6.995184247	0.005652759	1.074675385	34.60913019	29.99159434	0.027737159	0.516801721
8	1.346226465	49.53407069	1139.201066	3315.25828	1234.960195	4297.191917	443.8147364	52.57059352	0.004943965	0.000391782	6.995184247	0.005652759	1.074675385	31.22076327	31.40571391	0.027737159	0.516801721
9	1.346226465	49.53407069	1034.17763	3169.721094	1210.285036	3299.063923	375.4497993	52.57059352	0.004943965	0.000391782	6.995184247	0.005652759	1.074675385	31.40954983	28.6460425	0.027737159	0.516801721
10	1.346226465	49.53407069	1248.809816	3822.461194	1274.08907	4216.079225	424.5069775	52.57059352	0.011496459	0.000391782	6.995184247	0.005652759	1.074675385	28.38647822	31.42531545	0.027737159	0.516801721
11	1.346226465	49.53407069	1043.55211	3182.888522	1076.942852	3325.471523	345.9125695	52.57059352	0.010106504	0.000391782	6.995184247	0.005652759	1.074675385	36.8001073	34.7739007	0.027737159	0.516801721
12	1.346226465	49.53407069	1073.699099	3555.563689	1080.385163	3283.707535	342.5876175	52.57059352	0.030285689	0.00083927	29.81605535	0.044442115	1.074675385	33.85763651	44.30615969	0.027737159	0.516801721
13	1.346226465	49.53407069	1008.250307	2798.383463	1100.029956	3310.271112	387.7170892	52.57059352	0.004943965	0.000391782	6.995184247	0.005652759	1.074675385	27.41024553	27.76127786	0.027737159	0.516801721
14	1.346226465	49.53407069	986.9074852	3054.38528	999.5688243	3568.943376	368.7239874	52.57059352	0.004943965	0.000391782	6.995184247	0.005652759	1.074675385	28.55094974	29.40093486	0.027737159	0.516801721
15	1.346226465	49.53407069	1089.150074	3361.589926	1223.002291	3554.595694	298.8485188	52.57059352	0.004943965	0.000391782	6.995184247	0.012896696	1.074675385	29.59317018	32.44189436	0.027737159	0.516801721
16	1.346226465	49.53407069	1015.102034	2887.783367	1128.237355	3454.510716	284.0021386	52.57059352	0.004943965	0.000391782	6.995184247	0.005652759	1.074675385	26.12356472	28.63511216	0.027737159	0.516801721
17	1.346226465	49.53407069	1025.858248	3418.872651	907.6473461	4077.1772	225.0995124	52.57059352	0.004943965	0.000391782	6.995184247	0.005652759	1.074675385	25.93143565	29.26123962	0.027737159	0.516801721
18	1.346226465	49.53407069	1132.210111	3075.029254	1307.041507	4213.029771	287.4933598	52.57059352	0.004943965	0.000391782	6.995184247	0.005652759	1.074675385	28.81521316	30.27228576	0.027737159	0.516801721
19	1.346226465	49.53407069	1247.137538	3643.908529	1251.797181	3613.117747	336.1844339	52.57059352	0.004943965	0.000391782	6.995184247	0.005652759	1.074675385	32.01750569	33.30576421	0.027737159	1.740338553
20	1.346226465	49.53407069	977.8848772	2666.219387	978.8338463	3107.310646	351.9665638	52.57059352	0.004943965	0.000391782	6.995184247	0.005652759	1.074675385	31.67034951	26.74843787	0.027737159	0.516801721
21	1.346226465	49.53407069	936.2132989	2872.236213	1147.207993	3811.515768	321.2211536	52.57059352	0.004943965	0.000391782	6.995184247	0.005652759	1.074675385	27.52913427	30.70625205	0.027737159	0.516801721
22	1.346226465	49.53407069	1545.479808	4226.176512	1797.129582	5617.830062	590.868401	52.57059352	0.011939764	0.000391782	6.995184247	0.012103812	1.074675385	32.41561815	44.85167723	0.027737159	0.516801721
23	1.346226465	49.53407069	998.1880456	3148.543225	1019.103684	3461.981643	311.0504248	52.57059352	0.004943965	0.000391782	6.995184247	0.005652759	1.074675385	32.50630751	25.1136551	0.027737159	0.516801721
24	1.346226465	49.53407069	1044.091302	3157.256928	1183.048269	4130.356452	372.6547422	52.57059352	0.004943965	0.000391782	6.995184247	0.005652759	1.074675385	33.14206571	29.17224304	0.027737159	0.516801721
25	1.346226465	49.53407069	1091.991077	2992.163009	1205.511161	3461.699047	354.3227233	52.57059352	0.010956068	0.000391782	6.995184247	0.005652759	1.074675385	30.84718356	31.65456447	0.027737159	0.516801721
26	1.346226465	49.53407069	563.4750762	1712.719368	652.4827108	1892.765679	205.0561794	52.57059352	0.004943965	0.000391782	6.995184247	0.005652759	1.074675385	21.22565192	6.427420167	0.027737159	0.516801721
[illegible]																	

Samples	Cu	Zn	As	Se	Rb	Sr	Mo	Ag	Cd	Cs	Ba	Tl	Pb	U
1	3.786386576	23.2778783	0.008856552	0.09596498	14.4114451	5.687333565	0.514194292	0.006342015	0.017057123	0.028890999	4.688989018	0.000522746	0.032992059	0.002621754
2	3.563369651	25.33039115	0.006324761	0.110041893	13.94071236	5.272422496	0.500739007	0.006342015	0.067221519	0.029495207	3.987011506	0.000522746	0.032992059	0.002621754
3	3.847101188	26.15509136	0.002818625	0.091286211	12.50619059	3.493183841	0.6305776	0.006342015	0.066684179	0.021125566	3.303391172	0.000522746	0.032992059	0.002621754
4	3.75060959	23.09804694	0.005968606	0.07773271	12.31683395	5.297165386	0.509636129	0.006342015	0.0604362	0.024058599	4.169595131	0.000522746	0.032992059	0.002621754
5	3.101747677	28.30203855	0.006100001	0.120480683	12.10716793	6.533450646	0.411341869	0.006342015	0.017057123	0.021098202	5.623798265	0.000522746	0.032992059	0.002621754
6	2.550259674	20.52455244	0.011212006	0.145243234	10.74695659	4.378134037	0.365481038	0.006342015	0.017057123	0.02212599	3.880991483	0.000522746	0.032992059	0.002621754
7	3.331586654	25.0953784	0.01293224	0.131485849	13.90298368	4.657982692	0.549209466	0.006342015	0.017057123	0.021237912	4.452106604	0.000522746	0.032992059	0.002621754
8	3.44256661	22.98728432	0.006610085	0.116539026	17.0682656	5.361952943	0.395502484	0.006342015	0.017057123	0.018174009	4.177166675	0.000522746	0.032992059	0.002621754
9	3.073429608	22.76085483	0.006998471	0.124534072	11.17851651	5.251484936	0.620915178	0.006342015	0.017057123	0.025511109	4.519431862	0.000522746	0.032992059	0.002621754
10	4.547798671	28.88418167	0.017161552	0.157459987	15.10829053	7.995231468	0.300422089	0.006342015	0.017057123	0.026993086	11.60571612	0.000522746	0.032992059	0.002621754
11	3.750081687	26.14283714	0.002818625	0.139575488	10.44133354	5.65774318	0.453962797	0.006342015	0.017057123	0.01880799	8.297016253	0.000522746	0.032992059	0.002621754
12	3.387375378	26.45006772	0.011694947	0.093037673	13.53628526	3.827472966	0.366800323	0.006342015	0.017057123	0.033828045	4.348379507	0.000522746	0.032992059	0.002621754
13	2.973801165	19.66219649	0.009561763	0.070088967	9.974682559	5.227060963	0.489278885	0.006342015	0.056899084	0.019456914	4.57432304	0.000522746	0.032992059	0.002621754
14	2.444822267	21.00371709	0.002818625	0.104232247	12.11421155	5.30496659	0.577907239	0.006342015	0.017057123	0.023635677	4.458895924	0.000522746	0.032992059	0.002621754
15	3.461096163	26.52971133	0.002818625	0.096955675	10.048234	4.333395175	0.510228375	0.006342015	0.017057123	0.018231284	5.749716988	0.000522746	0.032992059	0.002621754
16	3.192829335	21.23414398	0.01043858	0.107418513	10.13932733	4.124682961	0.457097354	0.006342015	0.017057123	0.017192932	5.628522145	0.000522746	0.032992059	0.002621754
17	3.815784695	24.33006411	0.005884529	0.099801727	13.23099516	1.560061544	0.423998678	0.006342015	0.062965012	0.025196102	2.637702603	0.000522746	0.032992059	0.002621754
18	3.69587157	23.24995565	0.00655125	0.110884639	13.69239735	3.779408547	0.59000119	0.006342015	0.017057123	0.024495338	4.607520938	0.000522746	0.032992059	0.002621754
19	3.910356539	33.52820412	0.006036751	0.103946168	12.92195236	4.484736114	0.889057935	0.006342015	0.017057123	0.022364413	3.927515786	0.000522746	0.032992059	0.002621754
20	3.174572692	18.36528426	0.007158384	0.121612825	12.10672285	4.512945723	0.521829562	0.006342015	0.017057123	0.027192048	3.87674776	0.000522746	0.032992059	0.002621754
21	3.057028876	19.87825272	0.009670973	0.091186014	13.99406286	4.519599792	0.574732091	0.006342015	0.017057123	0.022773085	3.521002071	0.000522746	0.032992059	0.002621754
22	4.321142266	40.63162628	0.017726233	0.127625829	25.52583462	7.862994404	0.473973518	0.006342015	0.044566386	0.042054354	7.866897077	0.000522746	0.032992059	0.002621754
23	2.61464029	20.93781334	0.005770945	0.081432168	11.6713273	4.304502256	0.51849443	0.006342015	0.017057123	0.02552825	3.69989094	0.000522746	0.032992059	0.002621754
24	3.741590505	23.10326348	0.009737158	0.069025836	11.90870508	5.57724883	0.41108981	0.006342015	0.017057123	0.021371557	6.736634543	0.000522746	0.032992059	0.002621754
25	3.262962702	25.81537823	0.002818625	0.079505501	12.22703013	5.132453199	0.483230078	0.006342015	0.017057123	0.020261733	5.426734311	0.000522746	0.032992059	0.002621754
26	1.612276156	13.16061935	0.002818625	0.057020514	6.27792412	2.688494069	0.219002079	0.006342015	0.017057123	0.010869082	2.968898958	0.000522746	0.032992059	0.002621754
[illegible]														

Samples!	Year!	Year1!	Year2!	Site_Name!	Site2014!	site2015!	Site Ranking!	ZONE!	Soil!	HOSTILE_NOT!	SOIL_pH!	Replication!	Genotype!	Genotype Rank!	Species!	Plot_No!	TGW	GYD_t/ha	B	Na	Mg	P	S	K	Ca	Ti	Li	Be	Al	V	Cr	Mn	Fe	Co	Ni	Cu	Zn	As	Se	Rb	Sr	Mo	Ag	Cd	Cs	Ba	Tl	Pb	U
405	2014	2014		MALDA	MALDA		5	NEPZ	NORMAL	NOT_HOSTILE	7.7	2	HD_2967	15	Triticum_aestivum	45	44.3	3.8	1.346226465	49.53407069	1196.936936	3265.027595	1273.105027	3768.942764	350.5153499	52.57059352	0.004943965	0.000391782	6.995184247	0.005652759	1.074675385	39.77998867	35.66385845	0.027737159	0.516801721	3.736578851	31.14091034	0.008970162	0.080280921	9.937028647	1.589704204	0.330294025	0.006342015	0.017057123	0.0122879	5.709420627	0.000522746	0.032992059	0.002621754
406	2014	2014		MALDA	MALDA		5	NEPZ	NORMAL	NOT_HOSTILE	7.7	2	HI_1563	16	Triticum_aestivum	46	45.0	3.7	1.346226465	49.53407069	1165.532223	2984.662344	1304.436819	3284.099128	394.0774919	52.57059352	0.004943965	0.000391782	6.995184247	0.005652759	1.074675385	35.99208159	42.28052481	0.027737159	0.516801721	4.616369015	23.77837687	0.002818625	0.049729341	7.4948316	1.864826464	0.204183911	0.006342015	0.040241833	0.009851856	6.903920717	0.000522746	0.032992059	0.002621754
407	2014	2014		MALDA	MALDA		5	NEPZ	NORMAL	NOT_HOSTILE	7.7	2	NW_4018	28	Triticum_aestivum	47	37.3	2.8	1.346226465	49.53407069	950.4877691	3076.782558	1183.096923	3517.559155	261.0192995	52.57059352	0.004943965	0.000391782	6.995184247	0.005652759	1.074675385	34.93928702	31.83972208	0.027737159	0.516801721	3.449962211	21.20655181	0.002818625	0.068074241	8.398062781	1.206515499	0.360437698	0.006342015	0.017057123	0.00694108	3.954207617	0.000522746	0.032992059	0.002621754
408	2014	2014		MALDA	MALDA		5	NEPZ	NORMAL	NOT_HOSTILE	7.7	2	KRL_213	24	Triticum_aestivum	48	36.0	3.86	1.346226465	49.53407069	947.2691617	3193.229595	1204.070292	3919.777172	299.5540135	52.57059352	0.004943965	0.000391782	6.995184247	0.005652759	1.074675385	34.16399863	30.9111227	0.027737159	0.516801721	3.492116115	22.67617237	0.002818625	0.059180507	7.454750839	1.608537982	0.273411264	0.016040998	0.017057123	0.005088405	5.664067076	0.000522746	0.032992059	0.002621754
409	2014	2014		MALDA	MALDA		5	NEPZ	NORMAL	NOT_HOSTILE	7.7	2	KHARCHIA_65	20	Triticum_aestivum	49	37.4	1.08	1.346226465	49.53407069	1214.844391	3168.73113	1346.884262	4449.412817	356.7546377	52.57059352	0.004943965	0.000391782	6.995184247	0.005652759	1.074675385	38.28308728	43.84626059	0.027737159	0.516801721	4.11549715	23.11741393	0.002818625	0.042353545	11.13869122	1.373376756	0.179377673	0.006342015	0.039138512	0.011250676	4.495334086	0.000522746	0.032992059	0.002621754
410	2014	2014		MALDA	MALDA		5	NEPZ	NORMAL	NOT_HOSTILE	7.7	2	RW_3684	33	Triticum_aestivum	50	35.7	2.68	1.346226465	49.53407069	851.2288645	2907.719178	1184.363554	3791.999923	262.6681184	52.57059352	0.004943965	0.000391782	6.995184247	0.005652759	1.074675385	40.09418459	36.84709396	0.027737159	0.516801721	3.110910777	19.87026592	0.002818625	0.05683191	10.91507577	1.333306049	0.208562506	0.006342015	0.017057123	0.01178555	3.457824435	0.000522746	0.032992059	0.002621754
411	2014	2014		MALDA	MALDA		5	NEPZ	NORMAL	NOT_HOSTILE	7.7	2	NW_4092	29	Triticum_aestivum	51	35.1	3.16	1.346226465	49.53407069	1132.102747	3737.981282	1432.793994	4142.354692	333.460054	52.57059352	0.004943965	0.000391782	6.995184247	0.005652759	1.074675385	35.47152457	35.99517023	0.027737159	0.516801721	3.956085594	26.53043996	0.012870481	0.075206754	11.30117059	1.488621306	0.247177642	0.006342015	0.017057123	0.013019231	4.141817298	0.000522746	0.032992059	0.002621754
412	2014	2014		MALDA	MALDA		5	NEPZ	NORMAL	NOT_HOSTILE	7.7	2	GW_322	11	Triticum_aestivum	52	39.4	3.46	1.346226465	49.53407069	1006.255443	2855.932252	1188.2294	3784.464115	335.9325306	52.57059352	0.004943965	0.000391782	6.995184247	0.005652759	1.074675385	35.71565032	34.15836176	0.027737159	0.516801721	3.998843332	24.2364279	0.002818625	0.043538287	9.594793512	1.545182035	0.264905143	0.006342015	0.017057123	0.010693063	4.900715495	0.000522746	0.032992059	0.002621754
413	2014	2014		MALDA	MALDA		5	NEPZ	NORMAL	NOT_HOSTILE	7.7	2	UP_262	34	Triticum_aestivum	53	41.2	2.8	1.346226465	49.53407069	1270.104522	3463.985338	1413.380408	3936.70526	355.3491144	52.57059352	0.004943965	0.000391782	6.995184247	0.005652759	1.074675385	42.48114603	37.15873912	0.027737159	0.516801721	4.344385726	25.78398483	0.011423573	0.057077859	8.869776544	1.393270034	0.303136188	0.006342015	0.017057123	0.013483534	4.020613409	0.000522746	0.032992059	0.002621754
414	2014	2014		MALDA	MALDA		5	NEPZ	NORMAL	NOT_HOSTILE	7.7	2	DBW_51	8	Triticum_aestivum	54	37.9	3.48	1.346226465	49.53407069	972.2716759	3147.779812	1025.516253	3412.149013	299.3895306	52.57059352	0.004943965	0.000391782	6.995184247	0.005652759	1.074675385	33.32018094	28.85225913	0.027737159	0.516801721	3.024336816	22.33096481	0.002818625	0.063403875	6.432173667	1.468697347	0.234988769	0.006342015	0.017057123	0.00563549	5.481196559	0.000522746	0.032992059	0.002621754
415	2014	2014		MALDA	MALDA		5	NEPZ	NORMAL	NOT_HOSTILE	7.7	2	RAJ_4238	32	Triticum_aestivum	55	42.2	3.36	1.346226465	49.53407069	1167.783068	3280.290881	1297.172372	4043.269639	298.0118376	52.57059352	0.004943965	0.000391782	6.995184247	0.005652759	1.074675385	33.6164625	38.56144075	0.027737159	0.516801721	3.637612058	22.01655776	0.011234406	0.060111168	7.848807534	1.343392019	0.243503352	0.006342015	0.043432875	0.009476548	5.204284951	0.000522746	0.032992059	0.002621754
416	2014	2014		MALDA	MALDA		5	NEPZ	NORMAL	NOT_HOSTILE	7.7	2	DBW_17	5	Triticum_aestivum	56	37.9	3.3	1.346226465	49.53407069	1277.164179	4547.169209	1503.852524	4552.429574	263.2542641	52.57059352	0.004943965	0.000391782	6.995184247	0.005652759	1.074675385	44.90488962	38.52077523	0.027737159	0.516801721	3.647618115	33.65984934	0.002818625	0.049077506	10.94450356	1.630680639	0.228638544	0.006342015	0.04447965	0.012066627	5.470976134	0.000522746	0.032992059	0.002621754
417	2014	2014		MALDA	MALDA		5	NEPZ	NORMAL	NOT_HOSTILE	7.7	2	KRL_1-4	21	Triticum_aestivum	57	46.7	3.36	1.346226465	49.53407069	1253.065483	3326.445247	1571.730261	3996.680705	312.7960171	52.57059352	0.004943965	0.000391782	6.995184247	0.005652759	1.074675385	38.5811878	23.39135008	0.027737159	0.516801721	3.038217344	28.48961731	0.01617436	0.038205484	9.87525658	1.200597538	0.338388505	0.006342015	0.045536554	0.015717923	3.58071368	0.000522746	0.032992059	0.002621754
418	2014	2014		MALDA	MALDA		5	NEPZ	NORMAL	NOT_HOSTILE	7.7	2	WH_1105	36	Triticum_aestivum	58	35.4	3.16	1.346226465	49.53407069	1128.675982	2976.418253	1331.959585	3627.0827	381.8812322	52.57059352	0.004943965	0.000391782	6.995184247	0.005652759	1.074675385	37.6734404	31.01254728	0.027737159	0.516801721	4.282382657	24.13690255	0.002818625	0.072337432	9.416955576	1.705590505	0.275017384	0.006342015	0.017057123	0.013399015	4.157897693	0.000522746	0.032992059	0.002621754
419	2014	2014		MALDA	MALDA		5	NEPZ	NORMAL	NOT_HOSTILE	7.7	2	NW_1067	27	Triticum_aestivum	59	35.5	2.88	1.346226465	49.53407069	967.7732402	3136.587313	1253.704963	4371.681629	383.1773041	52.57059352	0.004943965	0.000391782	6.995184247	0.005652759	1.074675385	35.22220217	34.29237398	0.027737159	0.516801721	3.575943326	19.50328071	0.002818625	0.074923548	8.059575318	1.693071823	0.222485399	0.006342015	0.017057123	0.005725427	4.985234079	0.000522746	0.032992059	0.002621754
420	2014	2014		MALDA	MALDA		5	NEPZ	NORMAL	NOT_HOSTILE	7.7	2	DBW_39	6	Triticum_aestivum	60	49.6	3.76	1.346226465	49.53407069	1215.427632	3632.592911	1270.620637	4113.958032	251.8814922	52.57059352	0.004943965	0.000391782	6.995184247	0.005652759	1.074675385	38.2234798	33.54960489	0.027737159	0.516801721	3.631192434	32.58331917	0.008083965	0.118939872	6.824306107	1.250406367	0.422766072	0.013119812	0.017057123	0.005252764	4.754088287	0.000522746	0.032992059	0.002621754
421	2014	2014		MALDA	MALDA		5	NEPZ	NORMAL	NOT_HOSTILE	7.7	2	HD_2009	12	Triticum_aestivum	61	41.9	3.4	1.346226465	49.53407069	1064.874258	3074.166924	1389.033218	3384.543739	357.8446143	52.57059352	0.004943965	0.000391782	6.995184247	0.005652759	1.074675385	34.39407955	36.22204462	0.027737159	0.516801721	3.494295797	20.51083234	0.012017789	0.075491854	9.820596794	1.212615367	0.215638279	0.006342015	0.017057123	0.013721659	2.163648772	0.000522746	0.032992059	0.002621754
422	2014	2014		MALDA	MALDA		5	NEPZ	NORMAL	NOT_HOSTILE	7.7	2	K_0307	19	Triticum_aestivum	62	41.0	2.9	1.346226465	49.53407069	1418.268716	4256.241545	1556.059777	4849.686431	342.6957034	52.57059352	0.004943965	0.000391782	6.995184247	0.005652759	1.074675385	50.52284822	41.46853908	0.027737159	0.516801721	4.402018623	38.92123215	0.002818625	0.083136754	12.03681607	1.635124854	0.325330821	0.006342015	0.039745536	0.01689793	4.315743022	0.000522746	0.032992059	0.002621754
423	2014	2014		MALDA	MALDA		5	NEPZ	NORMAL	NOT_HOSTILE	7.7	2	KRL_3-4	25	Triticum_aestivum	63	33.9	1.52	1.346226465	49.53407069	1450.252023	4577.964295	1646.29013	4294.350525	375.8183386	52.57059352	0.004943965	0.000391782	6.995184247	0.005652759	1.074675385	46.0500176	44.06524777	0.027737159	0.516801721	3.625384906	39.02010668	0.007470384	0.088408515	15.74152203	1.519560122	0.279817433	0.006342015	0.062436964	0.019829571	3.36951677	0.000522746	0.032992059	0.002621754
424	2014	2014		MALDA	MALDA		5	NEPZ	NORMAL	NOT_HOSTILE	7.7	2	PDW_314	30	Triticum_turgidum	64	46.1	2.48	1.346226465	49.53407069	958.0117971	3357.129956	1210.753983	3768.977942	307.0176379	52.57059352	0.004943965	0.000391782	6.995184247	0.005652759	1.074675385	26.42616976	29.72029377	0.027737159	0.516801721	4.395637985	28.41452172	0.007051216	0.108599458	9.685096866	0.551870877	0.37470532	0.006342015	0.085156364	0.014679659	2.135320149	0.000522746	0.032992059	0.002621754
425	2014	2014		MALDA	MALDA		5	NEPZ	NORMAL	NOT_HOSTILE	7.7	2	KRL_210	23	Triticum_aestivum	65	32.9	3.32	1.346226465	49.53407069	1042.123414	2994.505093	1155.542509	3647.871998	310.4493428	52.57059352	0.004943965	0.000391782	6.995184247	0.005652759	1.074675385	42.93640554	33.93339778	0.027737159	0.516801721	3.643023424	23.14760618	0.002818625	0.059800981	9.355316118	1.630228471	0.24664797	0.015775829	0.017057123	0.009774293	4.445294303	0.000522746	0.032992059	0.002621754
426	2014	2014		MALDA	MALDA		5	NEPZ	NORMAL	NOT_HOSTILE	7.7	2	HI_8498	17	Triticum_turgidum	66	48.4	2.42	1.346226465	49.53407069	910.041882	3252.360052	1022.374091	3888.968034	235.4916564	52.57059352	0.004943965	0.000391782	6.995184247	0.005652759	1.074675385	27.07488328	29.32457852	0.027737159	0.516801721	4.050041971	27.25681771	0.002818625	0.100087507	7.536468491	0.463369544	0.242558717	0.006342015	0.017057123	0.010007061	2.397146139	0.000522746	0.032992059	0.002621754
427	2014	2014		MALDA	MALDA		5	NEPZ	NORMAL	NOT_HOSTILE	7.7	2	DBW_46	7	Triticum_aestivum	67	45.1	4.14	1.346226465	49.53407069	1036.545616	3284.321804	1108.217524	3508.76029	350.7340059	52.57059352	0.004943965	0.000391782	6.995184247	0.005652759	1.074675385	32.32035751	31.71258565	0.027737159	0.516801721	2.697399025	22.16299009	0.010718238	0.060661303	8.315588427	1.374932857	0.173891898	0.006342015	0.017057123	0.00881727	4.057932041	0.000522746	0.032992059	0.002621754
428	2014	2014		MALDA	MALDA		5	NEPZ	NORMAL	NOT_HOSTILE	7.7	2	RAJ_4229	31	Triticum_aestivum	68	38.1	3.6	1.346226465	49.53407069	1045.587364	2885.185523	1261.393164	3901.838432	288.7078737	52.57059352	0.004943965	0.000391782	6.995184247	0.005652759	1.074675385	29.3314528	34.70397291	0.027737159	0.516801721	3.742955276	18.50967569	0.014306762	0.056633528	6.841259565	1.082262648	0.191238213	0.006342015	0.017057123	0.007766393	4.978553374	0.000522746	0.032992059	0.002621754
429	2014	2014		MALDA	MALDA		5	NEPZ	NORMAL	NOT_HOSTILE	7.7	2	CBW_38	2	Triticum_aestivum	69	39.3	3.68	1.346226465	49.53407069	958.1290476	2872.815317	997.1880109	[illegible]	[illegible]	[illegible]	[illegible]	[illegible]	[illegible]	[illegible]	[illegible]	[illegible]	[illegible]	[illegible]	[illegible]	[illegible]	[illegible]	[illegible]	[illegible]	[illegible]	[illegible]	[illegible]	[illegible]	[illegible]	[illegible]	[illegible]	[illegible]	[illegible]	[illegible]

Samples!	Year!	Year1!	Year2!	Site_Name!	Site2014!	site2015!	Site Ranking!	ZONE!	Soil!	HOSTILE_NOT!	SOIL_pH!	Replication!	Genotype!	Genotype Rank!
565	2015		2015	HISAR		HISAR	2	NWPZ	SALINE	HOSTILE	8.1	2	MACS_6222	26
566	2015		2015	HISAR		HISAR	2	NWPZ	SALINE	HOSTILE	8.1	2	HD_2733	13
567	2015		2015	HISAR		HISAR	2	NWPZ	SALINE	HOSTILE	8.1	2	KRL_1-4	21
568	2015		2015	HISAR		HISAR	2	NWPZ	SALINE	HOSTILE	8.1	2	K_0307	19
569	2015		2015	HISAR		HISAR	2	NWPZ	SALINE	HOSTILE	8.1	2	KRL_19	22
570	2015		2015	HISAR		HISAR	2	NWPZ	SALINE	HOSTILE	8.1	2	HI_8498	17
571	2015		2015	HISAR		HISAR	2	NWPZ	SALINE	HOSTILE	8.1	2	WH_1105	36
572	2015		2015	HISAR		HISAR	2	NWPZ	SALINE	HOSTILE	8.1	2	RAJ_4229	31
573	2015		2015	HISAR		HISAR	2	NWPZ	SALINE	HOSTILE	8.1	2	NW_1067	27
574	2015		2015	HISAR		HISAR	2	NWPZ	SALINE	HOSTILE	8.1	2	DBW_71	9
575	2015		2015	HISAR		HISAR	2	NWPZ	SALINE	HOSTILE	8.1	2	RAJ_4238	32
576	2015		2015	HISAR		HISAR	2	NWPZ	SALINE	HOSTILE	8.1	2	DBW_16	4
577	2015		2015	KUMARGANJ-RECLAIMED		KUMARGANJ-RECLAIMED	3	NEPZ	NORMAL	NOT_HOSTILE	8.3	1	PDW_314	30
578	2015		2015	KUMARGANJ-RECLAIMED		KUMARGANJ-RECLAIMED	3	NEPZ	NORMAL	NOT_HOSTILE	8.3	1	HD_2967	15
579	2015		2015	KUMARGANJ-RECLAIMED		KUMARGANJ-RECLAIMED	3	NEPZ	NORMAL	NOT_HOSTILE	8.3	1	DPW_621-50	10
580	2015		2015	KUMARGANJ-RECLAIMED		KUMARGANJ-RECLAIMED	3	NEPZ	NORMAL	NOT_HOSTILE	8.3	1	DBW_17	5
581	2015		2015	KUMARGANJ-RECLAIMED		KUMARGANJ-RECLAIMED	3	NEPZ	NORMAL	NOT_HOSTILE	8.3	1	K_0307	19
582	2015		2015	KUMARGANJ-RECLAIMED		KUMARGANJ-RECLAIMED	3	NEPZ	NORMAL	NOT_HOSTILE	8.3	1	BH_1146	1
583	2015		2015	KUMARGANJ-RECLAIMED		KUMARGANJ-RECLAIMED	3	NEPZ	NORMAL	NOT_HOSTILE	8.3	1	RAJ_4229	31
584	2015		2015	KUMARGANJ-RECLAIMED		KUMARGANJ-RECLAIMED	3	NEPZ	NORMAL	NOT_HOSTILE	8.3	1	DBW_14	3
585	2015		2015	KUMARGANJ-RECLAIMED		KUMARGANJ-RECLAIMED	3	NEPZ	NORMAL	NOT_HOSTILE	8.3	1	DBW_46	7
586	2015		2015	KUMARGANJ-RECLAIMED		KUMARGANJ-RECLAIMED	3	NEPZ	NORMAL	NOT_HOSTILE	8.3	1	KHARCHIA_65	20
587	2015		2015	KUMARGANJ-RECLAIMED		KUMARGANJ-RECLAIMED	3	NEPZ	NORMAL	NOT_HOSTILE	8.3	1	DBW_16	4
588	2015		2015	KUMARGANJ-RECLAIMED		KUMARGANJ-RECLAIMED	3	NEPZ	NORMAL	NOT_HOSTILE	8.3	1	HI_8498	17
589	2015		2015	KUMARGANJ-RECLAIMED		KUMARGANJ-RECLAIMED	3	NEPZ	NORMAL	NOT_HOSTILE	8.3	1	MACS_6222	26
590	2015		2015	KUMARGANJ-RECLAIMED		KUMARGANJ-RECLAIMED	3	NEPZ	NORMAL	NOT_HOSTILE	8.3	1	DBW_71	9
591	2015		2015	KUMARGANJ-RECLAIMED		KUMARGANJ-RECLAIMED	3	NEPZ	NORMAL	NOT_HOSTILE	8.3	1	NW_4018	28
592	2015		2015	KUMARGANJ-RECLAIMED		KUMARGANJ-RECLAIMED	3	NEPZ	NORMAL	NOT_HOSTILE	8.3	1	NW_4092	29
593	2015		2015	KUMARGANJ-RECLAIMED		KUMARGANJ-RECLAIMED	3	NEPZ	NORMAL	NOT_HOSTILE	8.3	1	WH_1021	35
594	2015		2015	KUMARGANJ-RECLAIMED		KUMARGANJ-RECLAIMED	3	NEPZ	NORMAL	NOT_HOSTILE	8.3	1	KRL_19	22
595	2015		2015	KUMARGANJ-RECLAIMED		KUMARGANJ-RECLAIMED	3	NEPZ	NORMAL	NOT_HOSTILE	8.3	1	DBW_51	8
596	2015		2015	KUMARGANJ-RECLAIMED		KUMARGANJ-RECLAIMED	3	NEPZ	NORMAL	NOT_HOSTILE	8.3	1	HI_1563	16
597	2015		2015	KUMARGANJ-RECLAIMED		KUMARGANJ-RECLAIMED	3	NEPZ	NORMAL	NOT_HOSTILE	8.3	1	CBW_38	2
598	2015		2015	KUMARGANJ-RECLAIMED		KUMARGANJ-RECLAIMED	3	NEPZ	NORMAL	NOT_HOSTILE	8.3	1	GW_322	11
599	2015		2015	KUMARGANJ-RECLAIMED		KUMARGANJ-RECLAIMED	3	NEPZ	NORMAL	NOT_HOSTILE	8.3	1	RW_3684	33
600	2015		2015	KUMARGANJ-RECLAIMED		KUMARGANJ-RECLAIMED	3	NEPZ	NORMAL	NOT_HOSTILE	8.3	1	KRL_3-4	25
601	2015		2015	KUMARGANJ-RECLAIMED		KUMARGANJ-RECLAIMED	3	NEPZ	NORMAL	NOT_HOSTILE	8.3	1	KRL_1-4	21
602	2015		2015	KUMARGANJ-RECLAIMED		KUMARGANJ-RECLAIMED	3	NEPZ	NORMAL	NOT_HOSTILE	8.3	1	UP_262	34
603	2015		2015	KUMARGANJ-RECLAIMED		KUMARGANJ-RECLAIMED	3	NEPZ	NORMAL	NOT_HOSTILE	8.3	1	HD_2932	14
604	2015		2015	KUMARGANJ-RECLAIMED		KUMARGANJ-RECLAIMED	3	NEPZ	NORMAL	NOT_HOSTILE	8.3	1	HW_2044	18
605	2015		2015	KUMARGANJ-RECLAIMED		KUMARGANJ-RECLAIMED	3	NEPZ	NORMAL	NOT_HOSTILE	8.3	1	HD_2009	12
606	2015		2015	KUMARGANJ-RECLAIMED		KUMARGANJ-RECLAIMED	3	NEPZ	NORMAL	NOT_HOSTILE	8.3	1	HD_2733	13
607	2015		2015	KUMARGANJ-RECLAIMED		KUMARGANJ-RECLAIMED	3	NEPZ	NORMAL	NOT_HOSTILE	8.3	1	DBW_39	6
608	2015		2015	KUMARGANJ-RECLAIMED		KUMARGANJ-RECLAIMED	3	NEPZ	NORMAL	NOT_HOSTILE	8.3	1	NW_1067	27
609	2015		2015	KUMARGANJ-RECLAIMED		KUMARGANJ-RECLAIMED	3	NEPZ	NORMAL	NOT_HOSTILE	8.3	1	WH_1105	36
610	2015		2015	KUMARGANJ-RECLAIMED		KUMARGANJ-RECLAIMED	3	NEPZ	NORMAL	NOT_HOSTILE	8.3	1	RAJ_4238	32
611	2015		2015	KUMARGANJ-RECLAIMED		KUMARGANJ-RECLAIMED	3	NEPZ	NORMAL	NOT_HOSTILE	8.3	1	KRL_210	23
612	2015		2015	KUMARGANJ-RECLAIMED		KUMARGANJ-RECLAIMED	3	NEPZ	NORMAL	NOT_HOSTILE	8.3	1	KRL_213	24
613	2015		2015	KUMARGANJ-RECLAIMED		KUMARGANJ-RECLAIMED	3	NEPZ	NORMAL	NOT_HOSTILE	8.3	2	KRL_3-4	25
614	2015		2015	KUMARGANJ-RECLAIMED		KUMARGANJ-RECLAIMED	3	NEPZ	NORMAL	NOT_HOSTILE	8.3	2	UP_262	34
615	2015		2015	KUMARGANJ-RECLAIMED		KUMARGANJ-RECLAIMED	3	NEPZ	NORMAL	NOT_HOSTILE	8.3	2	KHARCHIA_65	20
616	2015		2015	KUMARGANJ-RECLAIMED		KUMARGANJ-RECLAIMED	3	NEPZ	NORMAL	NOT_HOSTILE	8.3	2	DBW_17	5
617	2015		2015	KUMARGANJ-RECLAIMED		KUMARGANJ-RECLAIMED	3	NEPZ	NORMAL	NOT_HOSTILE	8.3	2	DBW_71	9
618	2015		2015	KUMARGANJ-RECLAIMED		KUMARGANJ-RECLAIMED	3	NEPZ	NORMAL	NOT_HOSTILE	8.3	2	DBW_39	6
619	2015		2015	KUMARGANJ-RECLAIMED		KUMARGANJ-RECLAIMED	3	NEPZ	NORMAL	NOT_HOSTILE	8.3	2	WH_1021	35
620	2015		2015	KUMARGANJ-RECLAIMED		KUMARGANJ-RECLAIMED	3	NEPZ	NORMAL	NOT_HOSTILE	8.3	2	KRL_210	23
621	2015		2015	KUMARGANJ-RECLAIMED		KUMARGANJ-RECLAIMED	3	NEPZ	NORMAL	NOT_HOSTILE	8.3	2	DBW_16	4

Samples!	Year!	Year1!	Year2!	Site_Name!	Site2014!	site2015!	Site Ranking!	ZONE!	Soil!	HOSTILE_NOT!	SOIL_ pH!	Replication!	Genotype!	Genotype Rank!	Species!	Plot_No!	TGW	GYD_t/ha
812	2015		2015	MALDA		MALDA	5	NEPZ	NORMAL	NOT_HOSTILE	7.7	1	HI_1563	16	Triticum_aestivum	20	34.2	5.2
813	2015		2015	MALDA		MALDA	5	NEPZ	NORMAL	NOT_HOSTILE	7.7	1	KRL_3-4	25	Triticum_aestivum	21	22.2	1.62
814	2015		2015	MALDA		MALDA	5	NEPZ	NORMAL	NOT_HOSTILE	7.7	1	DBW_46	7	Triticum_aestivum	22	37.1	3.64
815	2015		2015	MALDA		MALDA	5	NEPZ	NORMAL	NOT_HOSTILE	7.7	1	NW_4092	29	Triticum_aestivum	23	40.3	5.32
816	2015		2015	MALDA		MALDA	5	NEPZ	NORMAL	NOT_HOSTILE	7.7	1	HD_2733	13	Triticum_aestivum	24	46.2	4.7
817	2015		2015	MALDA		MALDA	5	NEPZ	NORMAL	NOT_HOSTILE	7.7	1	KRL_210	23	Triticum_aestivum	25	36.7	4.46
818	2015		2015	MALDA		MALDA	5	NEPZ	NORMAL	NOT_HOSTILE	7.7	1	NW_4018	28	Triticum_aestivum	26	39.4	4.02
819	2015		2015	MALDA		MALDA	5	NEPZ	NORMAL	NOT_HOSTILE	7.7	1	RAJ_4238	32	Triticum_aestivum	27	33.0	4.78
820	2015		2015	MALDA		MALDA	5	NEPZ	NORMAL	NOT_HOSTILE	7.7	1	KHARCHIA_65	20	Triticum_aestivum	28	31.5	2.58
821	2015		2015	MALDA		MALDA	5	NEPZ	NORMAL	NOT_HOSTILE	7.7	1	CBW_38	2	Triticum_aestivum	29	37.5	4.86
822	2015		2015	MALDA		MALDA	5	NEPZ	NORMAL	NOT_HOSTILE	7.7	1	KRL_19	22	Triticum_aestivum	30	29.4	4.06
823	2015		2015	MALDA		MALDA	5	NEPZ	NORMAL	NOT_HOSTILE	7.7	1	DBW_51	8	Triticum_aestivum	31	38.4	4.34
824	2015		2015	MALDA		MALDA	5	NEPZ	NORMAL	NOT_HOSTILE	7.7	1	K_0307	19	Triticum_aestivum	32	38.6	4.8
825	2015		2015	MALDA		MALDA	5	NEPZ	NORMAL	NOT_HOSTILE	7.7	1	DPW_621-50	10	Triticum_aestivum	33	44.9	2.94
826	2015		2015	MALDA		MALDA	5	NEPZ	NORMAL	NOT_HOSTILE	7.7	1	HD_2932	14	Triticum_aestivum	34	33.1	4.56
827	2015		2015	MALDA		MALDA	5	NEPZ	NORMAL	NOT_HOSTILE	7.7	1	MACS_6222	26	Triticum_aestivum	35	34.8	4.88
828	2015		2015	MALDA		MALDA	5	NEPZ	NORMAL	NOT_HOSTILE	7.7	1	DBW_39	6	Triticum_aestivum	36	45.8	4.82
829	2015		2015	MALDA		MALDA	5	NEPZ	NORMAL	NOT_HOSTILE	7.7	2	MACS_6222	26	Triticum_aestivum	37	38.4	4.68
830	2015		2015	MALDA		MALDA	5	NEPZ	NORMAL	NOT_HOSTILE	7.7	2	WH_1021	35	Triticum_aestivum	38	41.2	5.3
831	2015		2015	MALDA		MALDA	5	NEPZ	NORMAL	NOT_HOSTILE	7.7	2	HD_2733	13	Triticum_aestivum	39	46.0	4.1
832	2015		2015	MALDA		MALDA	5	NEPZ	NORMAL	NOT_HOSTILE	7.7	2	HW_2044	18	Triticum_aestivum	40	34.6	4.46
833	2015		2015	MALDA		MALDA	5	NEPZ	NORMAL	NOT_HOSTILE	7.7	2	BH_1146	1	Triticum_aestivum	41	36.0	3.84
834	2015		2015	MALDA		MALDA	5	NEPZ	NORMAL	NOT_HOSTILE	7.7	2	KRL_19	22	Triticum_aestivum	42	28.3	4.08
835	2015		2015	MALDA		MALDA	5	NEPZ	NORMAL	NOT_HOSTILE	7.7	2	DBW_71	9	Triticum_aestivum	43	38.2	4.46
836	2015		2015	MALDA		MALDA	5	NEPZ	NORMAL	NOT_HOSTILE	7.7	2	DPW_621-50	10	Triticum_aestivum	44	35.8	4.24
837	2015		2015	MALDA		MALDA	5	NEPZ	NORMAL	NOT_HOSTILE	7.7	2	HD_2967	15	Triticum_aestivum	45	38.4	5.48
838	2015		2015	MALDA		MALDA	5	NEPZ	NORMAL	NOT_HOSTILE	7.7	2	HI_1563	16	Triticum_aestivum	46	32.9	4.82
839	2015		2015	MALDA		MALDA	5	NEPZ	NORMAL	NOT_HOSTILE	7.7	2	NW_4018	28	Triticum_aestivum	47	35.5	4.38
840	2015		2015	MALDA		MALDA	5	NEPZ	NORMAL	NOT_HOSTILE	7.7	2	KRL_213	24	Triticum_aestivum	48	31.8	5.96
841	2015		2015	MALDA		MALDA	5	NEPZ	NORMAL	NOT_HOSTILE	7.7	2	KHARCHIA_65	20	Triticum_aestivum	49	40.4	3.4
842	2015		2015	MALDA		MALDA	5	NEPZ	NORMAL	NOT_HOSTILE	7.7	2	RW_3684	33	Triticum_aestivum	50	34.4	4.82
843	2015		2015	MALDA		MALDA	5	NEPZ	NORMAL	NOT_HOSTILE	7.7	2	NW_4092	29	Triticum_aestivum	51	40.2	4.74
844	2015		2015	MALDA		MALDA	5	NEPZ	NORMAL	NOT_HOSTILE	7.7	2	GW_322	11	Triticum_aestivum	52	34.5	5.64
845	2015		2015	MALDA		MALDA	5	NEPZ	NORMAL	NOT_HOSTILE	7.7	2	UP_262	34	Triticum_aestivum	53	37.3	5.36
846	2015		2015	MALDA		MALDA	5	NEPZ	NORMAL	NOT_HOSTILE	7.7	2	DBW_51	8	Triticum_aestivum	54	38.4	5.02
847	2015		2015	MALDA		MALDA	5	NEPZ	NORMAL	NOT_HOSTILE	7.7	2	RAJ_4238	32	Triticum_aestivum	55	43.4	2.4
848	2015		2015	MALDA		MALDA	5	NEPZ	NORMAL	NOT_HOSTILE	7.7	2	DBW_17	5	Triticum_aestivum	56	34.1	3.6
849	2015		2015	MALDA		MALDA	5	NEPZ	NORMAL	NOT_HOSTILE	7.7	2	KRL_1-4	21	Triticum_aestivum	57	36.6	4.92
850	2015		2015	MALDA		MALDA	5	NEPZ	NORMAL	NOT_HOSTILE	7.7	2	WH_1105	36	Triticum_aestivum	58	32.3	4.22
851	2015		2015	MALDA		MALDA	5	NEPZ	NORMAL	NOT_HOSTILE	7.7	2	NW_1067	27	Triticum_aestivum	59	35.6	4.24
852	2015		2015	MALDA		MALDA	5	NEPZ	NORMAL	NOT_HOSTILE	7.7	2	DBW_39	6	Triticum_aestivum	60	42.4	5.68
853	2015		2015	MALDA		MALDA	5	NEPZ	NORMAL	NOT_HOSTILE	7.7	2	HD_2009	12	Triticum_aestivum	61	46.0	4.48
854	2015		2015	MALDA		MALDA	5	NEPZ	NORMAL	NOT_HOSTILE	7.7	2	K_0307	19	Triticum_aestivum	62	40.2	4.68
855	2015		2015	MALDA		MALDA	5	NEPZ	NORMAL	NOT_HOSTILE	7.7	2	KRL_3-4	25	Triticum_aestivum	63	19.6	1.44
856	2015		2015	MALDA		MALDA	5	NEPZ	NORMAL	NOT_HOSTILE	7.7	2	PDW_314	30	Triticum_turgidum	64	43.2	5.2
857	2015		2015	MALDA		MALDA	5	NEPZ	NORMAL	NOT_HOSTILE	7.7	2	KRL_210	23	Triticum_aestivum	65	33.6	6.22
858	2015		2015	MALDA		MALDA	5	NEPZ	NORMAL	NOT_HOSTILE	7.7	2	HI_8498	17	Triticum_turgidum	66	47.6	4.38
859	2015		2015	MALDA		MALDA	5	NEPZ	NORMAL	NOT_HOSTILE	7.7	2	DBW_46	7	Triticum_aestivum	67	43.1	3.98
860	2015		2015	MALDA		MALDA	5	NEPZ	NORMAL	NOT_HOSTILE	7.7	2	RAJ_4229	31	Triticum_aestivum	68	33.5	5.64
861	2015		2015	MALDA		MALDA	5	NEPZ	NORMAL	NOT_HOSTILE	7.7	2	CBW_38	2	Triticum_aestivum	69	36.1	6
862	2015		2015	MALDA		MALDA	5	NEPZ	NORMAL	NOT_HOSTILE	7.7	2	DBW_14	3	Triticum_aestivum	70	34.1	5.32
863	2015		2015	MALDA		MALDA	5	NEPZ	NORMAL	NOT_HOSTILE	7.7	2	HD_2932	14	Triticum_aestivum	71	32.2	5.9
864	2015		2015	MALDA		MALDA	5	NEPZ	NORMAL	NOT_HOSTILE	7.7	2	DBW_16	4	Triticum_aestivum	72	37.8	5.28

Samples!	B	Na	Mg	P	S	K	Ca	Ti	Li	Be	Al	V
812	1.346226465	49.53407069	1045.713252	3535.327312	1257.968235	3657.487695	343.3304606	52.57059352	0.004943965	0.000391782	6.995184247	0.005652759
813	1.346226465	49.53407069	1450.519743	4907.145377	2240.046459	5340.470749	434.5016623	52.57059352	0.004943965	0.000391782	6.995184247	0.005652759
814	1.346226465	49.53407069	993.2036552	3528.547674	1548.557687	3908.655552	340.2716294	52.57059352	0.004943965	0.000391782	6.995184247	0.005652759
815	1.346226465	49.53407069	991.5934578	3659.026337	1533.04599	3791.216734	299.4080461	52.57059352	0.004943965	0.000391782	6.995184247	0.005652759
816	1.346226465	49.53407069	1254.51358	4224.248112	1569.898925	4113.410578	259.9045276	52.57059352	0.004943965	0.000391782	6.995184247	0.005652759
817	1.346226465	49.53407069	946.8784551	3514.588355	1303.654748	4202.975908	322.5409683	52.57059352	0.004943965	0.000391782	6.995184247	0.005652759
818	1.346226465	49.53407069	888.7314872	3265.123212	1416.604604	3885.313916	236.3851027	52.57059352	0.004943965	0.000391782	6.995184247	0.005652759
819	1.346226465	49.53407069	1023.380845	3698.20473	1547.428491	4409.623979	296.3819018	52.57059352	0.004943965	0.000391782	6.995184247	0.005652759
820	1.346226465	49.53407069	1103.196813	3969.365532	1377.086741	4707.087026	332.2167155	52.57059352	0.004943965	0.000391782	6.995184247	0.005652759
821	1.346226465	49.53407069	1003.969268	3552.452113	1431.678139	3924.883691	270.4624502	52.57059352	0.004943965	0.000391782	6.995184247	0.005652759
822	1.346226465	49.53407069	1016.512996	3694.332282	1470.049696	4510.673401	360.5419494	52.57059352	0.004943965	0.000391782	6.995184247	0.005652759
823	1.346226465	49.53407069	1005.132003	3516.320833	1305.143329	4087.932697	255.8064377	52.57059352	0.004943965	0.000391782	6.995184247	0.005652759
824	1.346226465	49.53407069	1112.167661	3613.640199	1360.237307	3817.017633	334.8605922	52.57059352	0.004943965	0.000391782	6.995184247	0.005652759
825	1.346226465	49.53407069	1706.466256	4894.178631	2058.730235	3875.059572	392.0023797	52.57059352	0.004943965	0.000391782	6.995184247	0.005652759
826	1.346226465	49.53407069	1113.005311	3639.60547	1719.031939	3945.206142	321.6896342	52.57059352	0.004943965	0.000391782	6.995184247	0.005652759
827	1.346226465	49.53407069	969.8408969	3408.266755	1363.201214	4146.543005	342.1090389	52.57059352	0.004943965	0.000391782	6.995184247	0.005652759
828	1.346226465	49.53407069	1064.7041	3645.432987	1589.750222	3729.318336	238.1851002	52.57059352	0.004943965	0.000391782	6.995184247	0.005652759
829	1.346226465	49.53407069	937.3813421	3081.724393	1234.532801	4012.741973	300.3549973	52.57059352	0.004943965	0.000391782	6.995184247	0.005652759
830	1.346226465	49.53407069	932.512417	3045.42334	1268.089977	3756.146887	228.8907208	52.57059352	0.004943965	0.000391782	6.995184247	0.005652759
831	1.346226465	49.53407069	1205.413247	3782.335303	1786.877943	3753.811242	285.8341638	52.57059352	0.004943965	0.000391782	6.995184247	0.014911021
832	1.346226465	49.53407069	920.2926223	2933.367002	1355.008027	4197.547499	343.3624022	52.57059352	0.004943965	0.000391782	6.995184247	0.005652759
833	1.346226465	49.53407069	1103.04728	3784.000108	1827.285734	3508.197628	284.5005816	52.57059352	0.004943965	0.000391782	6.995184247	0.005652759
834	1.346226465	49.53407069	1054.685556	3509.157061	1551.099792	4470.509534	395.6064794	52.57059352	0.004943965	0.000391782	6.995184247	0.005652759
835	1.346226465	49.53407069	925.7378537	3396.814464	1326.480957	4210.030092	213.4234263	52.57059352	0.004943965	0.000391782	6.995184247	0.005652759
836	1.346226465	49.53407069	943.7193112	3371.463005	1342.144106	4342.219255	273.09379	52.57059352	0.004943965	0.000391782	6.995184247	0.005652759
837	1.346226465	49.53407069	1141.032131	3724.676472	1608.276219	4131.025509	315.5948129	52.57059352	0.004943965	0.000391782	6.995184247	0.005652759
838	1.346226465	49.53407069	988.7199209	3163.524783	1462.994652	3666.487173	349.3047625	52.57059352	0.004943965	0.000391782	6.995184247	0.005652759
839	1.346226465	49.53407069	1132.382798	3568.886339	1825.349351	3764.942523	289.3515637	52.57059352	0.004943965	0.000391782	6.995184247	0.005652759
840	1.346226465	49.53407069	835.3889998	3173.441325	1581.532581	3838.02142	272.6574946	52.57059352	0.004943965	0.000391782	6.995184247	0.005652759
841	1.346226465	49.53407069	933.1519179	3124.612554	1143.51429	3883.461735	270.071623	52.57059352	0.004943965	0.000391782	6.995184247	0.01686516
842	1.346226465	49.53407069	912.8596636	3291.037968	1358.641917	4072.596541	301.0146722	52.57059352	0.023212381	0.000391782	6.995184247	0.013096058
843	1.346226465	49.53407069	1197.356267	4324.99612	1825.170285	4158.094922	304.5166884	52.57059352	0.004943965	0.000391782	6.995184247	0.005652759
844	1.346226465	49.53407069	1031.733824	3436.942156	1479.403243	4366.713038	356.3770249	52.57059352	0.004943965	0.000391782	6.995184247	0.005652759
845	1.346226465	49.53407069	1104.307958	3301.002207	1723.211584	3612.867499	298.9882795	52.57059352	0.004943965	0.000391782	6.995184247	0.005652759
846	1.346226465	49.53407069	1113.171683	3992.567355	1687.090645	4021.845599	285.2851484	52.57059352	0.004943965	0.000391782	6.995184247	0.005652759
847	1.346226465	49.53407069	1438.855801	4685.155021	1866.464932	3723.174956	267.9325877	52.57059352	0.004943965	0.000391782	6.995184247	0.005652759
848	1.346226465	49.53407069	991.9968779	3715.179688	1433.397331	4292.111388	258.2139145	52.57059352	0.004943965	0.000391782	6.995184247	0.005652759
849	1.346226465	49.53407069	1132.826303	3494.442613	1531.630602	3827.660194	309.3067231	52.57059352	0.004943965	0.000391782	6.995184247	0.005652759
850	1.346226465	49.53407069	992.6695736	3402.722221	1542.278669	4034.428405	353.0146765	52.57059352	0.011452626	0.000391782	6.995184247	0.005652759
851	1.346226465	49.53407069	1087.577689	3830.800537	1807.496907	4785.787427	384.6794793	52.57059352	0.004943965	0.000391782	6.995184247	0.005652759
852	1.346226465	49.53407069	1122.068222	3829.889801	1529.967682	3969.776333	259.4558865	52.57059352	0.004943965	0.000391782	6.995184247	0.005652759
853	1.346226465	49.53407069	893.7987073	3427.0714	1377.034606	4185.082314	299.6636328	52.57059352	0.004943965	0.000391782	6.995184247	0.005652759
854	1.346226465	49.53407069	1064.50868	3452.091572	1369.282326	4003.704002	275.6331064	52.57059352	0.004943965	0.000391782	6.995184247	0.005652759
855	1.346226465	49.53407069	1436.155376	4339.686274	2097.538488	5151.50104	457.032522	52.57059352	0.004943965	0.000391782	6.995184247	0.005652759
856	1.346226465	49.53407069	874.0455572	3537.548146	1423.936594	4184.28425	299.1708798	52.57059352	0.004943965	0.000391782	6.995184247	0.005652759
857	1.346226465	49.53407069	1039.682448	3529.388403	1696.647176	3698.783393	403.0700534	52.57059352	0.004943965	0.000391782	6.995184247	0.005652759
858	1.346226465	49.53407069	978.2370609	3714.750735	1290.927588	4562.324846	285.1626625	52.57059352	0.004943965	0.000391782	6.995184247	0.005652759
859	1.346226465	49.53407069	1041.601415	3869.09292	1366.633631	4343.120725	294.8471877	52.57059352	0.004943965	0.000391782	6.995184247	0.005652759
860	1.346226465	49.53407069	1007.782199	3803.462638	1407.522975	4614.828194	258.4431783	52.57059352	0.004943965	0.000391782	6.995184247	0.005652759
861	1.346226465	49.53407069	1081.527362	3818.487459	1549.143022	4132.385044	239.8391529	52.57059352	0.004943965	0.000391782	6.995184247	0.005652759
862	1.346226465	49.53407069	1126.335684	3907.351057	1522.932073	3892.531946	346.8115899	52.57059352	0.004943965	0.000391782	6.995184247	0.005652759
863	1.346226465	49.53407069	1041.523171	3731.217077	1652.746927	4096.991292	329.4027817	52.57059352	0.004943965	0.000391782	6.995184247	0.005652759
864	1.346226465	49.53407069	1001.563226	3222.09357	1417.879408	3508.295999	284.9937185	52.57059352	0.004943965	0.000391782	6.995184247	0.005652759

Samples!	Cr	Mn	Fe	Co	Ni	Cu	Zn	As	Se
812	1.074675385	45.41255742	43.15880314	0.080958943	0.516801721	5.06019453	36.23205674	0.006075947	0.060637731
813	1.074675385	49.17841682	57.3066999	0.061773811	0.516801721	5.21191946	41.5466112	0.009981597	0.072282027
814	1.074675385	40.20003887	40.77985453	0.027737159	0.516801721	3.828720182	33.51314772	0.00610026	0.055740079
815	1.074675385	38.95215189	38.53227552	0.027737159	0.516801721	4.409602732	30.80568762	0.002818625	0.056779157
816	1.074675385	46.73674465	42.59414534	0.027737159	0.516801721	3.891585756	41.31012813	0.002818625	0.078249388
817	1.074675385	47.48878032	37.65717398	0.027737159	0.516801721	4.413010902	30.19339011	0.002818625	0.053603821
818	1.074675385	37.33340132	32.6711801	0.027737159	0.516801721	4.131055252	31.26721182	0.002818625	0.069540665
819	1.074675385	37.1414535	43.25159572	0.027737159	0.516801721	4.64467011	27.69219514	0.012253097	0.041724761
820	1.074675385	35.01431897	41.04644061	0.027737159	0.516801721	4.713637286	39.53300031	0.007508146	0.10641802
821	1.074675385	53.75029581	36.61652504	0.027737159	0.516801721	3.918536185	31.10503321	0.002818625	0.048133951
822	1.074675385	36.64535099	36.08600331	0.027737159	0.516801721	4.335715928	33.17569458	0.005988583	0.056462706
823	1.074675385	35.48877687	33.72535742	0.027737159	0.516801721	4.079849756	33.40406781	0.002818625	0.05631447
824	1.074675385	43.58690065	40.8719565	0.027737159	0.516801721	4.908650988	39.23074015	0.002818625	0.071094312
825	1.074675385	62.07790286	60.60906995	0.027737159	0.516801721	6.512395262	58.08166864	0.00762673	0.086571739
826	1.074675385	45.18222348	39.3101157	0.027737159	0.516801721	4.117371973	38.54870996	0.009818339	0.087340028
827	1.074675385	43.01264546	72.28225505	0.027737159	0.516801721	3.794414065	34.91769056	0.002818625	0.037618986
828	1.074675385	40.4348058	40.7128572	0.027737159	0.516801721	3.948019029	37.32376524	0.002818625	0.051056287
829	1.074675385	41.64096735	44.6793504	0.027737159	1.198820067	5.059188062	30.97809244	0.020932453	0.065444896
830	1.074675385	34.51632553	44.63809018	0.027737159	0.516801721	5.680301567	32.47517502	0.002818625	0.088209648
831	2.257866016	49.70692626	62.49306012	0.059901705	0.516801721	5.5133633	35.17269357	0.027901398	0.058377595
832	1.074675385	40.14291547	44.32110365	0.096886804	0.516801721	4.716514424	25.58066944	0.014463446	0.043642639
833	1.074675385	52.62845353	51.57017906	0.027737159	0.516801721	4.849779565	35.81696089	0.002818625	0.064091253
834	1.074675385	35.14063865	40.5502323	0.027737159	0.516801721	4.842163432	30.83489149	0.002818625	0.062253996
835	1.074675385	33.76936972	35.62470309	0.027737159	0.516801721	4.252638663	26.34730835	0.002818625	0.067676132
836	1.074675385	36.98098257	37.24596392	0.027737159	0.516801721	4.164498385	28.6573703	0.00650176	0.035841256
837	1.074675385	44.20268823	41.79397006	0.027737159	0.516801721	4.305013157	37.64730119	0.04052469	0.06724557
838	1.074675385	41.25421201	45.648407	0.027737159	0.516801721	5.022110621	29.1535282	0.002818625	0.061270537
839	1.074675385	45.70414841	48.45700699	0.027737159	0.516801721	5.857526212	35.60964631	0.0068478	0.056890881
840	1.074675385	39.30696056	45.78499629	0.027737159	0.516801721	6.109081153	27.6093957	0.010402559	0.061634674
841	4.211442367	38.2429024	59.04387785	0.058362336	2.132792827	5.084372102	34.51755954	0.011440779	0.079354795
842	1.074675385	44.45282164	37.2435284	0.027737159	0.516801721	4.297370186	29.00168535	0.015820101	0.07201486
843	1.074675385	44.68251392	48.80280914	0.027737159	0.516801721	5.075770504	41.46597323	0.020672973	0.049899541
844	1.074675385	42.22698801	35.5139898	0.027737159	0.516801721	4.734189348	29.62724995	0.008718934	0.043063811
845	1.074675385	42.6951917	44.49212658	0.027737159	0.516801721	4.361210672	33.92935524	0.002818625	0.047617234
846	1.074675385	45.20903567	44.00691636	0.027737159	0.516801721	4.452312458	42.41871208	0.03654667	0.07986998
847	1.074675385	53.64735133	50.6425172	0.027737159	0.516801721	5.666286989	52.12277561	0.007010277	0.141240081
848	1.074675385	40.04934753	36.79086883	0.027737159	0.516801721	4.453549891	35.50334462	0.007998302	0.042269842
849	1.074675385	43.57958581	47.15757621	0.027737159	0.516801721	6.236096457	28.61565496	0.007291787	0.038828055
850	1.074675385	48.22231625	50.66633337	0.027737159	0.516801721	5.686898436	28.60496442	0.012067674	0.056534225
851	1.074675385	44.81210965	52.71560208	0.027737159	1.266143337	4.952321453	30.18630581	0.002818625	0.059730962
852	1.074675385	48.75011618	46.93862022	0.027737159	0.516801721	4.244852837	33.1486182	0.002818625	0.05629204
853	1.074675385	33.79303913	37.57290324	0.027737159	0.516801721	3.972466526	24.23936392	0.002818625	0.037208898
854	1.074675385	46.06300548	42.03182396	0.027737159	0.516801721	4.5789183	32.97353724	0.002818625	0.037607954
855	1.074675385	42.59332608	50.14358477	0.062265787	0.516801721	4.421209406	38.503547	0.011819065	0.063072537
856	1.074675385	31.75325564	40.6815724	0.027737159	0.516801721	4.985809964	27.2502812	0.002818625	0.041140216
857	1.074675385	58.11509695	51.40428104	0.027737159	0.516801721	4.39475441	31.39486442	0.002818625	0.049486912
858	1.074675385	34.52487439	41.91036005	0.027737159	0.516801721	5.243822927	29.66133277	0.014345127	0.057927147
859	1.074675385	45.8026937	51.33890751	0.027737159	0.516801721	5.297597081	29.19889986	0.002818625	0.044084633
860	1.074675385	39.07610336	42.28340524	0.027737159	0.516801721	5.084745672	28.45178631	0.002818625	0.035783111
861	1.074675385	44.16790905	44.3826685	0.027737159	0.516801721	4.231083227	31.36262375	0.008059997	0.055176507
862	1.074675385	45.30448094	44.2936879	0.027737159	0.516801721	4.923343428	29.22100567	0.006476555	0.052393529
863	1.074675385	47.63276892	51.46794748	0.082216573	0.516801721	4.039425905	31.55927497	0.002818625	0.050969563
864	1.074675385	37.586693	40.44129329	0.027737159	0.516801721	4.547143724	32.53688275	0.002818625	0.073651398

Samples!	Rb	Sr	Mo	Ag	Cd	Cs	Ba	Tl	Pb	U
812	4.138514665	2.00593359	0.334773253	0.006342015	0.017057123	0.002928171	10.09967369	0.000522746	0.032992059	0.002621754
813	14.00365463	1.608571422	0.209054632	0.006342015	0.064949718	0.012963849	5.470597859	0.000522746	0.032992059	0.002621754
814	8.263007822	1.322528481	0.289323756	0.006342015	0.017057123	0.007536665	4.124941884	0.000522746	0.032992059	0.002621754
815	7.312458783	1.080107955	0.233844368	0.006342015	0.017057123	0.006835655	3.040990948	0.000522746	0.032992059	0.002621754
816	5.728227048	1.347539238	0.344305257	0.006342015	0.041446673	0.003237907	5.051138021	0.000522746	0.032992059	0.002621754
817	3.007693481	1.766548153	0.349148008	0.006342015	0.017057123	0.001398177	6.109181263	0.000522746	0.032992059	0.002621754
818	5.207747319	1.137924539	0.342702922	0.006342015	0.017057123	0.002787477	4.487895639	0.000522746	0.032992059	0.002621754
819	6.619144079	1.390939199	0.225611841	0.006342015	0.040584032	0.006724226	6.54527537	0.000522746	0.032992059	0.002621754
820	8.613023423	1.227749347	0.224752543	0.006342015	0.017057123	0.009088311	4.013985363	0.000522746	0.032992059	0.002621754
821	6.26983659	1.189117284	0.260736131	0.006342015	0.017057123	0.003010866	4.380950546	0.000522746	0.032992059	0.002621754
822	5.680265969	1.747273731	0.21401274	0.006342015	0.038586383	0.004017599	8.56149697	0.000522746	0.032992059	0.002621754
823	2.660607229	1.053599989	0.392501273	0.006342015	0.017057123	0.001835272	3.997797355	0.000522746	0.032992059	0.002621754
824	4.130269429	1.266360436	0.294010374	0.006342015	0.041451182	0.002523472	3.179515814	0.000522746	0.032992059	0.002621754
825	8.248043249	1.727631472	0.389777823	0.006342015	0.017057123	0.008085132	4.579308562	0.000522746	0.032992059	0.002621754
826	7.561118758	1.190830224	0.33643431	0.006342015	0.017057123	0.006484272	4.430943403	0.000522746	0.032992059	0.002621754
827	4.710118114	1.253150652	0.176765587	0.006342015	0.017057123	0.003078597	6.248238347	0.000522746	0.032992059	0.002621754
828	5.390776525	1.182902416	0.236340554	0.006342015	0.042423384	0.002975683	3.322803158	0.000522746	0.032992059	0.002621754
829	2.920475536	1.230926103	0.403707581	0.015481329	0.017057123	0.001349214	5.538089585	0.000522746	0.032992059	0.002621754
830	3.777294147	1.058192921	0.366473929	0.012916753	0.017057123	0.001991731	4.976435171	0.000522746	0.032992059	0.002621754
831	8.176624706	1.53266541	0.379852611	0.015051974	0.049279589	0.007037342	4.228973876	0.000522746	0.10478074	0.002621754
832	7.17594575	1.451879362	0.234554577	0.006342015	0.017057123	0.009089875	5.590544732	0.000522746	0.165284149	0.002621754
833	7.186632867	1.020439431	0.274159265	0.006342015	0.062820186	0.005385341	4.375331098	0.000522746	0.11090005	0.002621754
834	6.82407892	1.890139101	0.184100443	0.006342015	0.038447216	0.005138075	8.227545669	0.000522746	0.032992059	0.002621754
835	2.291376336	0.866403638	0.34875412	0.006342015	0.017057123	0.000992628	3.129057052	0.000522746	0.032992059	0.002621754
836	3.146003783	1.201229155	0.270320391	0.006342015	0.035231373	0.001761832	6.965708481	0.000522746	0.032992059	0.002621754
837	8.447886069	1.241165872	0.314904637	0.006342015	0.017057123	0.007086639	3.348822355	0.000522746	0.032992059	0.002621754
838	6.910210643	1.838407332	0.202819779	0.006342015	0.035562646	0.007048767	8.62356657	0.000522746	0.032992059	0.002621754
839	6.173821142	1.095196875	0.438335929	0.017835366	0.059473495	0.00479658	4.438789125	0.000522746	0.032992059	0.002621754
840	6.653073148	1.219721822	0.422010066	0.021968042	0.017057123	0.005141359	3.081556135	0.000522746	0.032992059	0.002621754
841	3.141761488	1.023379493	0.466965376	0.039977378	0.017057123	0.002699314	3.314802569	0.000522746	0.128063241	0.011896438
842	7.103858496	1.944654974	0.281732881	0.013427139	0.017057123	0.004148698	6.497383924	0.000522746	0.032992059	0.002621754
843	9.673834551	1.037227275	0.377230493	0.006342015	0.017057123	0.010684394	2.617063045	0.000522746	0.032992059	0.002621754
844	9.03852765	1.552046603	0.174767547	0.006342015	0.017057123	0.007668982	4.827995314	0.000522746	0.032992059	0.002621754
845	6.968416068	1.05558421	0.295177906	0.006342015	0.039019588	0.008235155	3.086297743	0.000522746	0.032992059	0.002621754
846	6.24397635	1.095011702	0.354756031	0.006342015	0.017057123	0.005223024	3.427429838	0.000522746	0.032992059	0.002621754
847	5.918299866	1.155914114	0.483056036	0.006342015	0.017057123	0.004485977	3.652316601	0.000522746	0.032992059	0.002621754
848	6.310074106	1.61856098	0.258496941	0.006342015	0.017057123	0.004550213	5.365377579	0.000522746	0.032992059	0.002621754
849	6.490543862	1.279584137	0.320287881	0.017239338	0.05781224	0.009262309	4.148555665	0.000522746	0.032992059	0.002621754
850	11.08503362	1.335757316	0.29030658	0.006342015	0.017057123	0.012797646	3.780592633	0.000522746	0.121728643	0.002621754
851	7.578920107	1.655668411	0.234044103	0.006342015	0.064079715	0.004004151	4.99064129	0.000522746	0.032992059	0.002621754
852	6.600345275	1.119872126	0.262384984	0.006342015	0.055879432	0.003726873	3.237728882	0.000522746	0.032992059	0.002621754
853	3.484809096	1.047483499	0.323084313	0.006342015	0.017057123	0.002785248	2.70348843	0.000522746	0.032992059	0.002621754
854	5.552866298	1.026042231	0.271383639	0.006342015	0.041042258	0.004991125	2.950541552	0.000522746	0.032992059	0.002621754
855	18.02526092	1.711793369	0.192183332	0.006342015	0.066332965	0.021695453	4.874812228	0.000522746	0.032992059	0.002621754
856	10.71311703	0.49677157	0.209356901	0.006342015	0.135846457	0.010058869	1.168014209	0.000522746	0.032992059	0.002621754
857	6.881218967	1.759844443	0.179140566	0.006342015	0.017057123	0.005204468	4.011357122	0.000522746	0.032992059	0.002621754
858	4.985609839	0.60443266	0.223730136	0.006342015	0.044374953	0.004683516	2.174011495	0.000522746	0.032992059	0.002621754
859	4.497953561	1.131263021	0.464938416	0.015989984	0.017057123	0.004347188	3.965241973	0.000522746	0.032992059	0.002621754
860	6.664733714	1.121888433	0.311852891	0.013611803	0.041524098	0.006316388	5.032674145	0.000522746	0.032992059	0.002621754
861	8.73363221	1.051171357	0.287903768	0.006342015	0.017057123	0.004801396	3.431249288	0.000522746	0.032992059	0.002621754
862	6.783066216	1.67035663	0.241484396	0.006342015	0.062379362	0.006223686	6.625042932	0.000522746	0.032992059	0.002621754
863	6.792907248	1.100759597	0.222179051	0.006342015	0.017057123	0.004547862	3.497133262	0.000522746	0.032992059	0.002621754
864	4.423078541	0.987388353	0.305214992	0.006342015	0.017057123	0.003842797	4.15105807	0.000522746	0.032992059	0.002621754
