# Supplementary material for: Variation in grain Zn concentration, and the grain ionome, in field-grown Indian wheat
Source: PLoS One. 2018 Jan 30;13(1):e0192026. doi: 10.1371/journal.pone.0192026 (PMC5790267; doi:10.1371/journal.pone.0192026)
Supplement: S4 Table — Data below limit of detection (LOD) have been replaced by half LOD values and values above than mean+(5*SD) have been removed. (PDF) [file pone.0192026.s004.pdf]

Supplementary Table 4. Data of grain mineral composition traits (mg/kg) of 36 genotypes evaluated at five sites in 2013-14 and at six sites in 2014-15. Data below limit of detection (LOD) have been replaced by half LOD values and data above mean+5*SD have been removed.

Samples	Year	Year1	Year2	Site_Name	Site2014	site2015	Site Ranking	ZONE	Soil	HOSTILE_NOT	SOIL_pH	Replication	Genotype	Genotype Rank	Species	Plot_No	TGW	GYD_t/ha
1	2014	2014		KARNAL	KARNAL		1	NWPZ	NORMAL	NOT_HOSTILE	7.5	1	WH_1105	36	Triticum_aestivum	1	38.35	8.016
2	2014	2014		KARNAL	KARNAL		1	NWPZ	NORMAL	NOT_HOSTILE	7.5	1	NW_4092	29	Triticum_aestivum	2	36.46	6.44
3	2014	2014		KARNAL	KARNAL		1	NWPZ	NORMAL	NOT_HOSTILE	7.5	1	DBW_16	4	Triticum_aestivum	3	38.64	5.44
4	2014	2014		KARNAL	KARNAL		1	NWPZ	NORMAL	NOT_HOSTILE	7.5	1	NW_4018	28	Triticum_aestivum	4	33.52	6.372
5	2014	2014		KARNAL	KARNAL		1	NWPZ	NORMAL	NOT_HOSTILE	7.5	1	HD_2733	13	Triticum_aestivum	5	32.15	3.88
6	2014	2014		KARNAL	KARNAL		1	NWPZ	NORMAL	NOT_HOSTILE	7.5	1	DBW_39	6	Triticum_aestivum	6	41.35	7.52
7	2014	2014		KARNAL	KARNAL		1	NWPZ	NORMAL	NOT_HOSTILE	7.5	1	CBW_38	2	Triticum_aestivum	7	40.83	7.208
8	2014	2014		KARNAL	KARNAL		1	NWPZ	NORMAL	NOT_HOSTILE	7.5	1	NW_1067	27	Triticum_aestivum	8	33.57	5.32
9	2014	2014		KARNAL	KARNAL		1	NWPZ	NORMAL	NOT_HOSTILE	7.5	1	DBW_51	8	Triticum_aestivum	9	44.18	6.64
10	2014	2014		KARNAL	KARNAL		1	NWPZ	NORMAL	NOT_HOSTILE	7.5	1	KRL_19	22	Triticum_aestivum	10	28.69	3.86
11	2014	2014		KARNAL	KARNAL		1	NWPZ	NORMAL	NOT_HOSTILE	7.5	1	HI_1563	16	Triticum_aestivum	11	39.50	5.776
12	2014	2014		KARNAL	KARNAL		1	NWPZ	NORMAL	NOT_HOSTILE	7.5	1	KHARCHIA_65	20	Triticum_aestivum	12	34.76	1.36
13	2014	2014		KARNAL	KARNAL		1	NWPZ	NORMAL	NOT_HOSTILE	7.5	1	HW_2044	18	Triticum_aestivum	13	45.53	6.12
14	2014	2014		KARNAL	KARNAL		1	NWPZ	NORMAL	NOT_HOSTILE	7.5	1	DBW_46	7	Triticum_aestivum	14	44.26	6.12
15	2014	2014		KARNAL	KARNAL		1	NWPZ	NORMAL	NOT_HOSTILE	7.5	1	RAJ_4238	32	Triticum_aestivum	15	40.23	5.332
16	2014	2014		KARNAL	KARNAL		1	NWPZ	NORMAL	NOT_HOSTILE	7.5	1	RAJ_4229	31	Triticum_aestivum	16	43.48	5.92
17	2014	2014		KARNAL	KARNAL		1	NWPZ	NORMAL	NOT_HOSTILE	7.5	1	HI_8498	17	Triticum_turgidum	17	50.67	5.88
18	2014	2014		KARNAL	KARNAL		1	NWPZ	NORMAL	NOT_HOSTILE	7.5	1	KRL_1-4	21	Triticum_aestivum	18	39.02	6.08
19	2014	2014		KARNAL	KARNAL		1	NWPZ	NORMAL	NOT_HOSTILE	7.5	1	UP_262	34	Triticum_aestivum	19	38.07	4.32
20	2014	2014		KARNAL	KARNAL		1	NWPZ	NORMAL	NOT_HOSTILE	7.5	1	DPW_621-50	10	Triticum_aestivum	20	42.26	7.32
21	2014	2014		KARNAL	KARNAL		1	NWPZ	NORMAL	NOT_HOSTILE	7.5	1	KRL_213	24	Triticum_aestivum	21	33.70	5.864
22	2014	2014		KARNAL	KARNAL		1	NWPZ	NORMAL	NOT_HOSTILE	7.5	1	KRL_3-4	25	Triticum_aestivum	22	24.74	0.564
23	2014	2014		KARNAL	KARNAL		1	NWPZ	NORMAL	NOT_HOSTILE	7.5	1	HD_2967	15	Triticum_aestivum	23	42.92	6.72
24	2014	2014		KARNAL	KARNAL		1	NWPZ	NORMAL	NOT_HOSTILE	7.5	1	GW_322	11	Triticum_aestivum	24	41.24	5.668
25	2014	2014		KARNAL	KARNAL		1	NWPZ	NORMAL	NOT_HOSTILE	7.5	1	WH_1021	35	[illegible]	[illegible]	[illegible]	[illegible]
26	2014	2014		KARNAL	KARNAL		1	NWPZ	NORMAL	NOT_HOSTILE	7.5	1	HD_2932	14	[illegible]	[illegible]	[illegible]	[illegible]
27	2014	2014		KARNAL	KARNAL		1	NWPZ	NORMAL	NOT_HOSTILE	7.5	1	MACS_6222	26	[illegible]	[illegible]	[illegible]	[illegible]
28	2014	2014		KARNAL	KARNAL		1	NWPZ	NORMAL	NOT_HOSTILE	7.5	1	DBW_17	5	[illegible]	[illegible]	[illegible]	[illegible]
29	2014	2014		KARNAL	KARNAL		1	NWPZ	NORMAL	NOT_HOSTILE	7.5	1	RW_3684	33	[illegible]	[illegible]	[illegible]	[illegible]
30	2014	2014		KARNAL	KARNAL		1	NWPZ	NORMAL	NOT_HOSTILE	7.5	1	PDW_314	30	[illegible]	[illegible]	[illegible]	[illegible]
31	2014	2014		KARNAL	KARNAL		1	NWPZ	NORMAL	NOT_HOSTILE	7.5	1	DBW_14	3	[illegible]	[illegible]	[illegible]	[illegible]
[illegible]	[illegible]	[illegible]	[illegible]	[illegible]	[illegible]	[illegible]	[illegible]	[illegible]	[illegible]	[illegible]	[illegible]	[illegible]	[illegible]	[illegible]	[illegible]	[illegible]	[illegible]	[illegible]

Samples	B	Na	Mg	P	S	K	Ca	Ti	Li	Be	Al	V	Cr	Mn
1	1.346226465	49.53407069	1175.43316	3385.658732	1247.314941	3562.390314	411.3237397	52.57059352	0.004943965	0.000391782	6.995184247	0.005652759	1.074675385	30.25917787
2	1.346226465	49.53407069	1128.571971	3070.286587	1327.611289	3549.260194	393.6351614	52.57059352	0.004943965	0.000391782	6.995184247	0.011711584	1.074675385	28.43342802
3	1.346226465	49.53407069	1130.968993	3095.299577	1155.635427	3714.209126	320.9045098	52.57059352	0.004943965	0.000391782	6.995184247	0.005652759	1.074675385	25.50411317
4	1.346226465	49.53407069	1019.811466	3035.768996	1181.685196	3597.712139	346.1703157	52.57059352	0.004943965	0.000391782	6.995184247	0.005652759	1.074675385	29.16014962
5	1.346226465	49.53407069	1141.16439	3382.799196	1204.606685	3522.221314	407.5533075	52.57059352	0.004943965	0.000391782	6.995184247	0.005652759	1.074675385	27.58646818
6	1.346226465	49.53407069	986.1999559	2884.535118	1099.406274	3286.044382	296.809891	52.57059352	0.010313704	0.000391782	6.995184247	0.005652759	1.074675385	27.87994358
7	1.346226465	49.53407069	1152.468623	3352.013697	1238.453228	3736.073138	322.8551253	52.57059352	0.004943965	0.000391782	6.995184247	0.005652759	1.074675385	34.60913019
8	1.346226465	49.53407069	1139.201066	3315.25828	1234.960195	4297.191917	443.8147364	52.57059352	0.004943965	0.000391782	6.995184247	0.005652759	1.074675385	31.22076327
9	1.346226465	49.53407069	1034.17763	3169.721094	1210.285036	3299.063923	375.4497993	52.57059352	0.004943965	0.000391782	6.995184247	0.005652759	1.074675385	31.40954983
10	1.346226465	49.53407069	1248.809816	3822.461194	1274.08907	4216.079225	424.5069775	52.57059352	0.011496459	0.000391782	6.995184247	0.005652759	1.074675385	28.38647822
11	1.346226465	49.53407069	1043.55211	3182.888522	1076.942852	3325.471523	345.9125695	52.57059352	0.010106504	0.000391782	6.995184247	0.005652759	1.074675385	36.8001073
12	1.346226465	49.53407069	1073.699099	3555.563689	1080.385163	3283.707535	342.5876175	52.57059352	0.030285689	0.00083927	29.81605535	0.044442115	1.074675385	33.85763651
13	1.346226465	49.53407069	1008.250307	2798.383463	1100.029956	3310.271112	387.7170892	52.57059352	0.004943965	0.000391782	6.995184247	0.005652759	1.074675385	27.41024553
14	1.346226465	49.53407069	986.9074852	3054.38528	999.5688243	3568.943376	368.7239874	52.57059352	0.004943965	0.000391782	6.995184247	0.005652759	1.074675385	28.55094974
15	1.346226465	49.53407069	1089.150074	3361.589926	1223.002291	3554.595694	298.8485188	52.57059352	0.004943965	0.000391782	6.995184247	0.012896696	1.074675385	29.59317018
16	1.346226465	49.53407069	1015.102034	2887.783367	1128.237355	3454.510716	284.0021386	52.57059352	0.004943965	0.000391782	6.995184247	0.005652759	1.074675385	26.12356472
17	1.346226465	49.53407069	1025.858248	3418.872651	907.6473461	4077.1772	225.0995124	52.57059352	0.004943965	0.000391782	6.995184247	0.005652759	1.074675385	25.93143565
18	1.346226465	49.53407069	1132.210111	3075.029254	1307.041507	4213.029771	287.4933598	52.57059352	0.004943965	0.000391782	6.995184247	0.005652759	1.074675385	28.81521316
19	1.346226465	49.53407069	1247.137538	3643.908529	1251.797181	3613.117747	336.1844339	52.57059352	0.004943965	0.000391782	6.995184247	0.005652759	1.074675385	32.01750569
20	1.346226465	49.53407069	977.8848772	2666.219387	978.8338463	3107.310646	351.9665638	52.57059352	0.004943965	0.000391782	6.995184247	0.005652759	1.074675385	31.67034951
21	1.346226465	49.53407069	936.2132989	2872.236213	1147.207993	3811.515768	321.2211536	52.57059352	0.004943965	0.000391782	6.995184247	0.005652759	1.074675385	27.52913427
22	1.346226465	49.53407069	1545.479808	4226.176512	1797.129582	5617.830062	590.868401	52.57059352	0.011939764	0.000391782	6.995184247	0.012103812	1.074675385	32.41561815
23	1.346226465	49.53407069	998.1880456	3148.543225	1019.103684	3461.981643	311.0504248	52.57059352	0.004943965	0.000391782	6.995184247	0.005652759	1.074675385	32.50630751
24	1.346226465	49.53407069	1044.091302	3157.256928	1183.048269	4130.356452	372.6547422	52.57059352	0.004943965	0.000391782	6.995184247	0.005652759	1.074675385	33.14206571
[illegible]	[illegible]	[illegible]	[illegible]	[illegible]	[illegible]	[illegible]	[illegible]	[illegible]	[illegible]	[illegible]	[illegible]	[illegible]	[illegible]	[illegible]

Samples	Fe	Co	Ni	Cu	Zn	As	Se	Rb	Sr	Mo	Ag	Cd	Cs	Ba	Tl	Pb	U
1	30.86099462	0.027737159	0.516801721	3.786386576	23.2778783	0.008856552	0.09596498	14.4114451	5.687333565	0.514194292	0.006342015	0.017057123	0.028890999	4.688989018	0.000522746	0.032992059	0.002621754
2	29.16197862	0.027737159	0.516801721	3.563369651	25.33039115	0.006324761	0.110041893	13.94071236	5.272422496	0.500739007	0.006342015	0.067221519	0.029495207	3.987011506	0.000522746	0.032992059	0.002621754
3	33.7911061	0.027737159	0.516801721	3.847101188	26.15509136	0.002818625	0.091286211	12.50619059	3.493183841	0.6305776	0.006342015	0.066684179	0.021125566	3.303391172	0.000522746	0.032992059	0.002621754
4	29.09682393	0.027737159	0.516801721	3.75060959	23.09804694	0.005968606	0.07773271	12.31683395	5.297165386	0.509636129	0.006342015	0.0604362	0.024058599	4.169595131	0.000522746	0.032992059	0.002621754
5	26.22012739	0.027737159	0.516801721	3.101747677	28.30203855	0.006100001	0.120480683	12.10716793	6.533450646	0.411341869	0.006342015	0.017057123	0.021098202	5.623798265	0.000522746	0.032992059	0.002621754
6	30.99653844	0.027737159	0.516801721	2.550259674	20.52455244	0.011212006	0.145243234	10.74695659	4.378134037	0.365481038	0.006342015	0.017057123	0.02212599	3.880991483	0.000522746	0.032992059	0.002621754
7	29.99159434	0.027737159	0.516801721	3.331586654	25.0953784	0.01293224	0.131485849	13.90298368	4.657982692	0.549209466	0.006342015	0.017057123	0.021237912	4.452106604	0.000522746	0.032992059	0.002621754
8	31.40571391	0.027737159	0.516801721	3.44256661	22.98728432	0.006610085	0.116539026	17.0682656	5.361952943	0.395502484	0.006342015	0.017057123	0.018174009	4.177166675	0.000522746	0.032992059	0.002621754
9	28.6460425	0.027737159	0.516801721	3.073429608	22.76085483	0.006998471	0.124534072	11.17851651	5.251484936	0.620915178	0.006342015	0.017057123	0.025511109	4.519431862	0.000522746	0.032992059	0.002621754
10	31.42531545	0.027737159	0.516801721	4.547798671	28.88418167	0.017161552	0.157459987	15.10829053	7.995231468	0.300422089	0.006342015	0.017057123	0.026993086	11.60571612	0.000522746	0.032992059	0.002621754
11	34.7739007	0.027737159	0.516801721	3.750081687	26.14283714	0.002818625	0.139575488	10.44133354	5.65774318	0.453962797	0.006342015	0.017057123	0.01880799	8.297016253	0.000522746	0.032992059	0.002621754
12	44.30615969	0.027737159	0.516801721	3.387375378	26.45006772	0.011694947	0.093037673	13.53628526	3.827472966	0.366800323	0.006342015	0.017057123	0.033828045	4.348379507	0.000522746	0.032992059	0.002621754
13	27.76127786	0.027737159	0.516801721	2.973801165	19.66219649	0.009561763	0.070088967	9.974682559	5.227060963	0.489278885	0.006342015	0.056899084	0.019456914	4.57432304	0.000522746	0.032992059	0.002621754
14	29.40093486	0.027737159	0.516801721	2.444822267	21.00371709	0.002818625	0.104232247	12.11421155	5.30496659	0.577907239	0.006342015	0.017057123	0.023635677	4.458895924	0.000522746	0.032992059	0.002621754
15	32.44189436	0.027737159	0.516801721	3.461096163	26.52971133	0.002818625	0.096955675	10.048234	4.333395175	0.510228375	0.006342015	0.017057123	0.018231284	5.749716988	0.000522746	0.032992059	0.002621754
16	28.63511216	0.027737159	0.516801721	3.192829335	21.23414398	0.01043858	0.107418513	10.13932733	4.124682961	0.457097354	0.006342015	0.017057123	0.017192932	5.628522145	0.000522746	0.032992059	0.002621754
17	29.26123962	0.027737159	0.516801721	3.815784695	24.33006411	0.005884529	0.099801727	13.23099516	1.560061544	0.423998678	0.006342015	0.062965012	0.025196102	2.637702603	0.000522746	0.032992059	0.002621754
18	30.27228576	0.027737159	0.516801721	3.69587157	23.24995565	0.00655125	0.110884639	13.69239735	3.779408547	0.59000119	0.006342015	0.017057123	0.024495338	4.607520938	0.000522746	0.032992059	0.002621754
19	33.30576421	0.027737159	1.740338553	3.910356539	33.52820412	0.006036751	0.103946168	12.92195236	4.484736114	0.889057935	0.006342015	0.017057123	0.022364413	3.927515786	0.000522746	0.032992059	0.002621754
20	26.74843787	0.027737159	0.516801721	3.174572692	18.36528426	0.007158384	0.121612825	12.10672285	4.512945723	0.521829562	0.006342015	0.017057123	0.027192048	3.87674776	0.000522746	0.032992059	0.002621754
21	30.70625205	0.027737159	0.516801721	3.057028876	19.87825272	0.009670973	0.091186014	13.99406286	4.519599792	0.574732091	0.006342015	0.017057123	0.022773085	3.521002071	0.000522746	0.032992059	0.002621754
22	44.85167723	0.027737159	0.516801721	4.321142266	40.63162628	0.017726233	0.127625829	25.52583462	7.862994404	0.473973518	0.006342015	0.044566386	0.042054354	7.866897077	0.000522746	0.032992059	0.002621754
23	25.1136551	0.027737159	0.516801721	2.61464029	20.93781334	0.005770945	0.081432168	11.6713273	4.304502256	0.51849443	0.006342015	0.017057123	0.02552825	3.69989094	0.000522746	0.032992059	0.002621754
24	29.17224304	0.027737159	0.516801721	3.741590505	23.10326348	0.009737158	0.069025836	11.90870508	5.57724883	0.41108981	0.006342015	0.017057123	0.021371557	6.736634543	0.000522746	0.032992059	0.002621754
[illegible]	[illegible]	[illegible]	[illegible]	[illegible]	[illegible]	[illegible]	[illegible]	[illegible]	[illegible]	[illegible]	[illegible]	[illegible]	[illegible]	[illegible]	[illegible]	[illegible]	[illegible]

Samples!	Year!	Year1!	Year2!	Site_Name!	Site2014!	site2015!	Site Ranking!	ZONE!	Soil!	HOSTILE_NOT!	SOIL_pH!	Replication!	Genotype!	Genotype Rank!	Species!
405	2014	2014		MALDA	MALDA		5	NEPZ	NORMAL	NOT_HOSTILE	7.7	2	HD_2967	15	Triticum_aestivum
406	2014	2014		MALDA	MALDA		5	NEPZ	NORMAL	NOT_HOSTILE	7.7	2	HI_1563	16	Triticum_aestivum
407	2014	2014		MALDA	MALDA		5	NEPZ	NORMAL	NOT_HOSTILE	7.7	2	NW_4018	28	Triticum_aestivum
408	2014	2014		MALDA	MALDA		5	NEPZ	NORMAL	NOT_HOSTILE	7.7	2	KRL_213	24	Triticum_aestivum
409	2014	2014		MALDA	MALDA		5	NEPZ	NORMAL	NOT_HOSTILE	7.7	2	KHARCHIA_65	20	Triticum_aestivum
410	2014	2014		MALDA	MALDA		5	NEPZ	NORMAL	NOT_HOSTILE	7.7	2	RW_3684	33	Triticum_aestivum
411	2014	2014		MALDA	MALDA		5	NEPZ	NORMAL	NOT_HOSTILE	7.7	2	NW_4092	29	Triticum_aestivum
412	2014	2014		MALDA	MALDA		5	NEPZ	NORMAL	NOT_HOSTILE	7.7	2	GW_322	11	Triticum_aestivum
413	2014	2014		MALDA	MALDA		5	NEPZ	NORMAL	NOT_HOSTILE	7.7	2	UP_262	34	Triticum_aestivum
414	2014	2014		MALDA	MALDA		5	NEPZ	NORMAL	NOT_HOSTILE	7.7	2	DBW_51	8	Triticum_aestivum
415	2014	2014		MALDA	MALDA		5	NEPZ	NORMAL	NOT_HOSTILE	7.7	2	RAJ_4238	32	Triticum_aestivum
416	2014	2014		MALDA	MALDA		5	NEPZ	NORMAL	NOT_HOSTILE	7.7	2	DBW_17	5	Triticum_aestivum
417	2014	2014		MALDA	MALDA		5	NEPZ	NORMAL	NOT_HOSTILE	7.7	2	KRL_1-4	21	Triticum_aestivum
418	2014	2014		MALDA	MALDA		5	NEPZ	NORMAL	NOT_HOSTILE	7.7	2	WH_1105	36	Triticum_aestivum
419	2014	2014		MALDA	MALDA		5	NEPZ	NORMAL	NOT_HOSTILE	7.7	2	NW_1067	27	Triticum_aestivum
420	2014	2014		MALDA	MALDA		5	NEPZ	NORMAL	NOT_HOSTILE	7.7	2	DBW_39	6	Triticum_aestivum
421	2014	2014		MALDA	MALDA		5	NEPZ	NORMAL	NOT_HOSTILE	7.7	2	HD_2009	12	Triticum_aestivum
422	2014	2014		MALDA	MALDA		5	NEPZ	NORMAL	NOT_HOSTILE	7.7	2	K_0307	19	Triticum_aestivum
423	2014	2014		MALDA	MALDA		5	NEPZ	NORMAL	NOT_HOSTILE	7.7	2	KRL_3-4	25	Triticum_aestivum
424	2014	2014		MALDA	MALDA		5	NEPZ	NORMAL	NOT_HOSTILE	7.7	2	PDW_314	30	Triticum_turgidum
425	2014	2014		MALDA	MALDA		5	NEPZ	NORMAL	NOT_HOSTILE	7.7	2	KRL_210	23	Triticum_aestivum
426	2014	2014		MALDA	MALDA		5	NEPZ	NORMAL	NOT_HOSTILE	7.7	2	HI_8498	17	Triticum_turgidum
427	2014	2014		MALDA	MALDA		5	NEPZ	NORMAL	NOT_HOSTILE	7.7	2	DBW_46	7	Triticum_aestivum
428	2014	2014		MALDA	MALDA		5	NEPZ	NORMAL	NOT_HOSTILE	7.7	2	RAJ_4229	31	Triticum_aestivum
429	2014	2014		MALDA	MALDA		5	NEPZ	NORMAL	NOT_HOSTILE	7.7	2	CBW_38	2	Triticum_aestivum
430	2014	2014		MALDA	MALDA		5	NEPZ	NORMAL	NOT_HOSTILE	7.7	2	DBW_14	3	Triticum_aestivum
431	2014	2014		MALDA	MALDA		5	NEPZ	NORMAL	NOT_HOSTILE	7.7	2	HD_2932	14	Triticum_aestivum
432	2014	2014		MALDA	MALDA		5	NEPZ	NORMAL	NOT_HOSTILE	7.7	2	DBW_16	4	Triticum_aestivum
433	2015		2015	KARNAL		KARNAL	1	NWPZ	NORMAL	NOT_HOSTILE	7.5	1	WH_1105	36	Triticum_aestivum
434	2015		2015	KARNAL		KARNAL	1	NWPZ	NORMAL	NOT_HOSTILE	7.5	1	NW_4092	29	Triticum_aestivum
435	2015		2015	KARNAL		KARNAL	1	NWPZ	NORMAL	NOT_HOSTILE	7.5	1	DBW_16	4	Triticum_aestivum
436	2015		2015	KARNAL		KARNAL	1	NWPZ	NORMAL	NOT_HOSTILE	7.5	1	NW_4018	28	Triticum_aestivum
437	2015		2015	KARNAL		KARNAL	1	NWPZ	NORMAL	NOT_HOSTILE	7.5	1	HD_2733	13	Triticum_aestivum
438	2015		2015	KARNAL		KARNAL	1	NWPZ	NORMAL	NOT_HOSTILE	7.5	1	DBW_39	6	Triticum_aestivum
439	2015		2015	KARNAL		KARNAL	1	NWPZ	NORMAL	NOT_HOSTILE	7.5	1	CBW_38	2	Triticum_aestivum
440	2015		2015	KARNAL		KARNAL	1	NWPZ	NORMAL	NOT_HOSTILE	7.5	1	NW_1067	27	Triticum_aestivum
441	2015		2015	KARNAL		KARNAL	1	NWPZ	NORMAL	NOT_HOSTILE	7.5	1	DBW_51	8	Triticum_aestivum
442	2015		2015	KARNAL		KARNAL	1	NWPZ	NORMAL	NOT_HOSTILE	7.5	1	KRL_19	22	Triticum_aestivum
443	2015		2015	KARNAL		KARNAL	1	NWPZ	NORMAL	NOT_HOSTILE	7.5	1	HI_1563	16	Triticum_aestivum
444	2015		2015	KARNAL		KARNAL	1	NWPZ	NORMAL	NOT_HOSTILE	7.5	1	KHARCHIA_65	20	Triticum_aestivum
445	2015		2015	KARNAL		KARNAL	1	NWPZ	NORMAL	NOT_HOSTILE	7.5	1	HW_2044	18	Triticum_aestivum
446	2015		2015	KARNAL		KARNAL	1	NWPZ	NORMAL	NOT_HOSTILE	7.5	1	DBW_46	7	Triticum_aestivum
447	2015		2015	KARNAL		KARNAL	1	NWPZ	NORMAL	NOT_HOSTILE	7.5	1	RAJ_4238	32	Triticum_aestivum
448	2015		2015	KARNAL		KARNAL	1	NWPZ	NORMAL	NOT_HOSTILE	7.5	1	RAJ_4229	31	Triticum_aestivum
449	2015		2015	KARNAL		KARNAL	1	NWPZ	NORMAL	NOT_HOSTILE	7.5	1	HI_8498	17	Triticum_turgidum
450	2015		2015	KARNAL		KARNAL	1	NWPZ	NORMAL	NOT_HOSTILE	7.5	1	KRL_1-4	21	Triticum_aestivum
451	2015		2015	KARNAL		KARNAL	1	NWPZ	NORMAL	NOT_HOSTILE	7.5	1	UP_262	34	Triticum_aestivum
452	2015		2015	KARNAL		KARNAL	1	NWPZ	NORMAL	NOT_HOSTILE	7.5	1	DPW_621-50	10	Triticum_aestivum
453	2015		2015	KARNAL		KARNAL	1	NWPZ	NORMAL	NOT_HOSTILE	7.5	1	KRL_213	24	Triticum_aestivum
454	2015		2015	KARNAL		KARNAL	1	NWPZ	NORMAL	NOT_HOSTILE	7.5	1	KRL_3-4	25	Triticum_aestivum
455	2015		2015	KARNAL		KARNAL	1	NWPZ	NORMAL	NOT_HOSTILE	7.5	1	HD_2967	15	Triticum_aestivum
456	2015		2015	KARNAL		KARNAL	1	NWPZ	NORMAL	NOT_HOSTILE	7.5	1	GW_322	11	Triticum_aestivum
457	2015		2015	KARNAL		KARNAL	1	NWPZ	NORMAL	NOT_HOSTILE	7.5	1	WH_1021	35	Triticum_aestivum
458	2015		2015	KARNAL		KARNAL	1	NWPZ	NORMAL	NOT_HOSTILE	7.5	1	HD_2932	14	Triticum_aestivum
459	2015		2015	KARNAL		KARNAL	1	NWPZ	NORMAL	NOT_HOSTILE	7.5	1	MACS_6222	26	Triticum_aestivum
460	2015		2015	KARNAL		KARNAL	1	NWPZ	NORMAL	NOT_HOSTILE	7.5	1	DBW_17	5	Triticum_aestivum
461	2015		2015	KARNAL		KARNAL	1	NWPZ	NORMAL	NOT_HOSTILE	7.5	1	RW_3684	33	Triticum_aestivum
462	2015		2015	KARNAL		KARNAL	1	NWPZ	NORMAL	NOT_HOSTILE	7.5	1	PDW_314	30	Triticum_turgidum
463	2015		2015	KARNAL		KARNAL	1	NWPZ	NORMAL	NOT_HOSTILE	7.5	1	DBW_14	3	Triticum_aestivum
464	2015		2015	KARNAL		KARNAL	1	NWPZ	NORMAL	NOT_HOSTILE	7.5	1	KRL_210	23	Triticum_aestivum
465	2015		2015	KARNAL		KARNAL	1	NWPZ	NORMAL	NOT_HOSTILE	7.5	1	HD_2009	12	Triticum_aestivum
466	2015		2015	KARNAL		KARNAL	1	NWPZ	NORMAL	NOT_HOSTILE	7.5	1	DBW_71	9	Triticum_aestivum
467	2015		2015	KARNAL		KARNAL	1	NWPZ	NORMAL	NOT_HOSTILE	7.5	1	BH_1146	1	Triticum_aestivum
468	2015		2015	KARNAL		KARNAL	1	NWPZ	NORMAL	NOT_HOSTILE	7.5	1	K_0307	19	Triticum_aestivum
469	2015		2015	KARNAL		KARNAL	1	NWPZ	NORMAL	NOT_HOSTILE	7.5	2	GW_322	11	Triticum_aestivum
470	2015		2015	KARNAL		KARNAL	1	NWPZ	NORMAL	NOT_HOSTILE	7.5	2	WH_1105	36	Triticum_aestivum
471	2015		2015	KARNAL		KARNAL	1	NWPZ	NORMAL	NOT_HOSTILE	7.5	2	DBW_17	5	Triticum_aestivum
472	2015		2015	KARNAL		KARNAL	1	NWPZ	NORMAL	NOT_HOSTILE	7.5	2	BH_1146	1	Triticum_aestivum
473	2015		2015	KARNAL		KARNAL	1	NWPZ	NORMAL	NOT_HOSTILE	7.5	2	RAJ_4229	31	Triticum_aestivum
474	2015		2015	KARNAL		KARNAL	1	NWPZ	NORMAL	NOT_HOSTILE	7.5	2	NW_1067	27	Triticum_aestivum
475	2015		2015	KARNAL		KARNAL	1	NWPZ	NORMAL	NOT_HOSTILE	7.5	2	HI_1563	16	Triticum_aestivum
476	2015		2015	KARNAL		KARNAL	1	NWPZ	NORMAL	NOT_HOSTILE	7.5	2	RAJ_4238	32	Triticum_aestivum
477	2015		2015	KARNAL		KARNAL	1	NWPZ	NORMAL	NOT_HOSTILE	7.5	2	KRL_213	24	Triticum_aestivum
478	2015		2015	KARNAL		KARNAL	1	NWPZ	NORMAL	NOT_HOSTILE	7.5	2	HD_2932	14	Triticum_aestivum
479	2015		2015	KARNAL		KARNAL	1	NWPZ	NORMAL	NOT_HOSTILE	7.5	2	K_0307	19	Triticum_aestivum
480	2015		2015	KARNAL		KARNAL	1	NWPZ	NORMAL	NOT_HOSTILE	7.5	2	NW_4018	28	Triticum_aestivum
481	2015		2015	KARNAL		KARNAL	1	NWPZ	NORMAL	NOT_HOSTILE	7.5	2	WH_1021	35	Triticum_aestivum
482	2015		2015	KARNAL		KARNAL	1	NWPZ	NORMAL	NOT_HOSTILE	7.5	2	DBW_51	8	Triticum_aestivum
483	2015		2015	KARNAL		KARNAL	1	NWPZ	NORMAL	NOT_HOSTILE	7.5	2	HD_2733	13	Triticum_aestivum
484	2015		2015	KARNAL		KARNAL	1	NWPZ	NORMAL	NOT_HOSTILE	7.5	2	HW_2044	18	Triticum_aestivum
485	2015		2015	KARNAL		KARNAL	1	NWPZ	NORMAL	NOT_HOSTILE	7.5	2	DPW_621-50	10	Triticum_aestivum
486	2015		2015	KARNAL		KARNAL	1	NWPZ	NORMAL	NOT_HOSTILE	7.5	2	DBW_71	9	Triticum_aestivum
487	2015		2015	KARNAL		KARNAL	1	NWPZ	NORMAL	NOT_HOSTILE	7.5	2	KHARCHIA_65	20	Triticum_aestivum
488	2015		2015	KARNAL		KARNAL	1	NWPZ	NORMAL	NOT_HOSTILE	7.5	2	UP_262	34	Triticum_aestivum
489	2015		2015	KARNAL		KARNAL	1	NWPZ	NORMAL	NOT_HOSTILE	7.5	2	DBW_46	7	Triticum_aestivum
490	2015		2015	KARNAL		KARNAL	1	NWPZ	NORMAL	NOT_HOSTILE	7.5	2	MACS_6222	26	Triticum_aestivum
491	2015		2015	KARNAL		KARNAL	1	NWPZ	NORMAL	NOT_HOSTILE	7.5	2	DBW_16	4	Triticum_aestivum
492	2015		2015	KARNAL		KARNAL	1	NWPZ	NORMAL	NOT_HOSTILE	7.5	2	HD_2009	12	Triticum_aestivum
493	2015		2015	KARNAL		KARNAL	1	NWPZ	NORMAL	NOT_HOSTILE	7.5	2	KRL_210	23	Triticum_aestivum
494	2015		2015	KARNAL		KARNAL	1	NWPZ	NORMAL	NOT_HOSTILE	7.5	2	CBW_38	2	Triticum_aestivum
495	2015		2015	KARNAL		KARNAL	1	NWPZ	NORMAL	NOT_HOSTILE	7.5	2	PDW_314	30	Triticum_turgidum
496	2015		2015	KARNAL		KARNAL	1	NWPZ	NORMAL	NOT_HOSTILE	7.5	2	KRL_3-4	25	Triticum_aestivum
497	2015		2015	KARNAL		KARNAL	1	NWPZ	NORMAL	NOT_HOSTILE	7.5	2	HI_8498	17	Triticum_turgidum
498	2015		2015	KARNAL		KARNAL	1	NWPZ	NORMAL	NOT_HOSTILE	7.5	2	NW_4092	29	Triticum_aestivum
499	2015		2015	KARNAL		KARNAL	1	NWPZ	NORMAL	NOT_HOSTILE	7.5	2	KRL_1-4	21	Triticum_aestivum
500	2015		2015	KARNAL		KARNAL	1	NWPZ	NORMAL	NOT_HOSTILE	7.5	2	DBW_14	3	Triticum_aestivum
501	2015		2015	KARNAL		KARNAL	1	NWPZ	NORMAL	NOT_HOSTILE	7.5	2	DBW_39	6	Triticum_aestivum
502	2015		2015	KARNAL		KARNAL	1	NWPZ	NORMAL	NOT_HOSTILE	7.5	2	HD_2967	15	Triticum_aestivum
503	2015		2015	KARNAL		KARNAL	1	NWPZ	NORMAL	NOT_HOSTILE	7.5	2	KRL_19	22	Triticum_aestivum
504	2015		2015	KARNAL		KARNAL	1	NWPZ	NORMAL	NOT_HOSTILE	7.5	2	RW_3684	33	Triticum_aestivum
505	2015		2015	HISAR		HISAR	2	NWPZ	SALINE	HOSTILE	8.1	1	KRL_3-4	25	Triticum_aestivum
506	2015		2015	HISAR		HISAR	2	NWPZ	SALINE	HOSTILE	8.1	1	KRL_19	22	Triticum_aestivum
507	2015		2015	HISAR		HISAR	2	NWPZ	SALINE	HOSTILE	8.1	1	GW_322	11	Triticum_aestivum
508	2015		2015	HISAR		HISAR	2	NWPZ	SALINE	HOSTILE	8.1	1	NW_1067	27	Triticum_aestivum
509	2015		2015	HISAR		HISAR	2	NWPZ	SALINE	HOSTILE	8.1	1	DBW_17	5	Triticum_aestivum
510	2015		2015	HISAR		HISAR	2	NWPZ	SALINE	HOSTILE	8.1	1	HD_2967	15	Triticum_aestivum
511	2015		2015	HISAR		HISAR	2	NWPZ	SALINE	HOSTILE	8.1	1	KRL_1-4	21	Triticum_aestivum
512	2015		2015	HISAR		HISAR	2	NWPZ	SALINE	HOSTILE	8.1	1	HW_2044	18	Triticum_aestivum
513	2015		2015	HISAR		HISAR	2	NWPZ	SALINE	HOSTILE	8.1	1	RAJ_4238	32	Triticum_aestivum
514	2015		2015	HISAR		HISAR	2	NWPZ	SALINE	HOSTILE	8.1	1	HD_2932	14	Triticum_aestivum
515	2015		2015	HISAR		HISAR	2	NWPZ	SALINE	HOSTILE	8.1	1	UP_262	34	Triticum_aestivum
516	2015		2015	HISAR		HISAR	2	NWPZ	SALINE	HOSTILE	8.1	1	PDW_314	30	Triticum_turgidum
517	2015		2015	HISAR		HISAR	2	NWPZ	SALINE	HOSTILE	8.1	1	BH_1146	1	Triticum_aestivum
518	2015		2015	HISAR		HISAR	2	NWPZ	SALINE	HOSTILE	8.1	1	K_0307	19	Triticum_aestivum
519	2015		2015	HISAR		HISAR	2	NWPZ	SALINE	HOSTILE	8.1	1	DBW_71	9	Triticum_aestivum
520	2015		2015	HISAR		HISAR	2	NWPZ	SALINE	HOSTILE	8.1	1	NW_4092	29	Triticum_aestivum
521	2015		2015	HISAR		HISAR	2	NWPZ	SALINE	HOSTILE	8.1	1	CBW_38	2	Triticum_aestivum
522	2015		2015	HISAR		HISAR	2	NWPZ	SALINE	HOSTILE	8.1	1	DBW_46	7	Triticum_aestivum
523	2015		2015	HISAR		HISAR	2	NWPZ	SALINE	HOSTILE	8.1	1	HD_2733	13	Triticum_aestivum
524	2015		2015	HISAR		HISAR	2	NWPZ	SALINE	HOSTILE	8.1	1	DBW_51	8	Triticum_aestivum
525	2015		2015	HISAR		HISAR	2	NWPZ	SALINE	HOSTILE	8.1	1	NW_4018	28	Triticum_aestivum
526	2015		2015	HISAR		HISAR	2	NWPZ	SALINE	HOSTILE	8.1	1	HI_1563	16	Triticum_aestivum
527	2015		2015	HISAR		HISAR	2	NWPZ	SALINE	HOSTILE	8.1	1	RAJ_4229	31	Triticum_aestivum
528	2015		2015	HISAR		HISAR	2	NWPZ	SALINE	HOSTILE	8.1	1	HD_2009	12	Triticum_aestivum
529	2015		2015	HISAR		HISAR	2	NWPZ	SALINE	HOSTILE	8.1	1	HI_8498	17	Triticum_turgidum
530	2015		2015	HISAR		HISAR	2	NWPZ	SALINE	HOSTILE	8.1	1	RW_3684	33	Triticum_aestivum
531	2015		2015	HISAR		HISAR	2	NWPZ	SALINE	HOSTILE	8.1	1	WH_1105	36	Triticum_aestivum
532	2015		2015	HISAR		HISAR	2	NWPZ	SALINE	HOSTILE	8.1	1	DPW_621-50	10	Triticum_aestivum
533	2015		2015	HISAR		HISAR	2	NWPZ	SALINE	HOSTILE	8.1	1	DBW_14	3	Triticum_aestivum
534	2015		2015	HISAR		HISAR	2	NWPZ	SALINE	HOSTILE	8.1	1	KRL_210	23	Triticum_aestivum
535	2015		2015	HISAR		HISAR	2	NWPZ	SALINE	HOSTILE	8.1	1	DBW_16	4	Triticum_aestivum
536	2015		2015	HISAR		HISAR	2	NWPZ	SALINE	HOSTILE	8.1	1	MACS_6222	26	Triticum_aestivum
537	2015		2015	HISAR		HISAR	2	NWPZ	SALINE	HOSTILE	8.1	1	WH_1021	35	Triticum_aestivum
538	2015		2015	HISAR		HISAR	2	NWPZ	SALINE	HOSTILE	8.1	1	KRL_213	24	Triticum_aestivum
539	2015		2015	HISAR		HISAR	2	NWPZ	SALINE	HOSTILE	8.1	1	KHARCHIA_65	20	Triticum_aestivum
540	2015		2015	HISAR		HISAR	2	NWPZ	SALINE	HOSTILE	8.1	1	DBW_39	6	Triticum_aestivum
541	2015		2015	HISAR		HISAR	2	NWPZ	SALINE	HOSTILE	8.1	2	WH_1021	35	Triticum_aestivum
542	2015		2015	HISAR		HISAR	2	NWPZ	SALINE	HOSTILE	8.1	2	DBW_51	8	Triticum_aestivum
543	2015		2015	HISAR		HISAR	2	NWPZ	SALINE	HOSTILE	8.1	2	DBW_46	7	Triticum_aestivum
544	2015		2015	HISAR		HISAR	2	NWPZ	SALINE	HOSTILE	8.1	2	DPW_621-50	10	Triticum_aestivum
545	2015		2015	HISAR		HISAR	2	NWPZ	SALINE	HOSTILE	8.1	2	HD_2932	14	Triticum_aestivum
546	2015		2015	HISAR		HISAR	2	NWPZ	SALINE	HOSTILE	8.1	2	GW_322	11	Triticum_aestivum
547	2015		2015	HISAR		HISAR	2	NWPZ	SALINE	HOSTILE	8.1	2	PDW_314	30	Triticum_turgidum
548	2015		2015	HISAR		HISAR	2	NWPZ	SALINE	HOSTILE	8.1	2	KRL_3-4	25	Triticum_aestivum
549	2015		2015	HISAR		HISAR	2	NWPZ	SALINE	HOSTILE	8.1	2	KHARCHIA_65	20	Triticum_aestivum
550	2015		2015	HISAR		HISAR	2	NWPZ	SALINE	HOSTILE	8.1	2	HD_2009	12	Triticum_aestivum
551	2015		2015	HISAR		HISAR	2	NWPZ	SALINE	HOSTILE	8.1	2	NW_4092	29	Triticum_aestivum
552	2015		2015	HISAR		HISAR	2	NWPZ	SALINE	HOSTILE	8.1	2	RW_3684	33	Triticum_aestivum
553	2015		2015	HISAR		HISAR	2	NWPZ	SALINE	HOSTILE	8.1	2	HW_2044	18	Triticum_aestivum
554	2015		2015	HISAR		HISAR	2	NWPZ	SALINE	HOSTILE	8.1	2	DBW_14	3	Triticum_aestivum
555	2015		2015	HISAR		HISAR	2	NWPZ	SALINE	HOSTILE	8.1	2	NW_4018	28	Triticum_aestivum
556	2015		2015	HISAR		HISAR	2	NWPZ	SALINE	HOSTILE	8.1	2	BH_1146	1	Triticum_aestivum
557	2015		2015	HISAR		HISAR	2	NWPZ	SALINE	HOSTILE	8.1	2	HD_2967	15	Triticum_aestivum
558	2015		2015	HISAR		HISAR	2	NWPZ	SALINE	HOSTILE	8.1	2	KRL_213	24	Triticum_aestivum
559	2015		2015	HISAR		HISAR	2	NWPZ	SALINE	HOSTILE	8.1	2	DBW_39	6	Triticum_aestivum
560	2015		2015	HISAR		HISAR	2	NWPZ	SALINE	HOSTILE	8.1	2	HI_1563	16	Triticum_aestivum
561	2015		2015	HISAR		HISAR	2	NWPZ	SALINE	HOSTILE	8.1	2	UP_262	34	Triticum_aestivum
562	2015		2015	HISAR		HISAR	2	NWPZ	SALINE	HOSTILE	8.1	2	CBW_38	2	Triticum_aestivum
563	2015		2015	HISAR		HISAR	2	NWPZ	SALINE	HOSTILE	8.1	2	DBW_17	5	Triticum_aestivum
564	2015		2015	HISAR		HISAR	2	NWPZ	SALINE	HOSTILE	8.1	2	KRL_210	23	Triticum_aestivum
565	2015		2015	HISAR		HISAR	2	NWPZ	SALINE	HOSTILE	8.1	2	MACS_6222	26	Triticum_aestivum
566	2015		2015	HISAR		HISAR	2	NWPZ	SALINE	HOSTILE	8.1	2	HD_2733	13	Triticum_aestivum
567	2015		2015	HISAR		HISAR	2	NWPZ	SALINE	HOSTILE	8.1	2	KRL_1-4	21	Triticum_aestivum
568	2015		2015	HISAR		HISAR	2	NWPZ	SALINE	HOSTILE	8.1	2	K_0307	19	Triticum_aestivum
569	2015		2015	HISAR		HISAR	2	NWPZ	SALINE	HOSTILE	8.1	2	KRL_19	22	Triticum_aestivum
570	2015		2015	HISAR		HISAR	2	NWPZ	SALINE	HOSTILE	8.1	2	HI_8498	17	Triticum_turgidum
571	2015		2015	HISAR		HISAR	2	NWPZ	SALINE	HOSTILE	8.1	2	WH_1105	36	Triticum_aestivum
572	2015		2015	HISAR		HISAR	2	NWPZ	SALINE	HOSTILE	8.1	2	RAJ_4229	31	Triticum_aestivum
573	2015		2015	HISAR		HISAR	2	NWPZ	SALINE	HOSTILE	8.1	2	NW_1067	27	Triticum_aestivum
574	2015		2015	HISAR		HISAR	2	NWPZ	SALINE	HOSTILE	8.1	2	DBW_71	9	Triticum_aestivum
575	2015		2015	HISAR		HISAR	2	NWPZ	SALINE	HOSTILE	8.1	2	RAJ_4238	32	Triticum_aestivum
576	2015		2015	HISAR		HISAR	2	NWPZ	SALINE	HOSTILE	8.1	2	DBW_16	4	Triticum_aestivum
577	2015		2015	KUMARGANJ-RECLAIMED		KUMARGANJ-RECLAIMED	3	NEPZ	NORMAL	NOT_HOSTILE	8.3	1	PDW_314	30	Triticum_turgidum
578	2015		2015	KUMARGANJ-RECLAIMED		KUMARGANJ-RECLAIMED	3	NEPZ	NORMAL	NOT_HOSTILE	8.3	1	HD_2967	15	Triticum_aestivum
579	2015		2015	KUMARGANJ-RECLAIMED		KUMARGANJ-RECLAIMED	3	NEPZ	NORMAL	NOT_HOSTILE	8.3	1	DPW_621-50	10	Triticum_aestivum
580	2015		2015	KUMARGANJ-RECLAIMED		KUMARGANJ-RECLAIMED	3	NEPZ	NORMAL	NOT_HOSTILE	8.3	1	DBW_17	5	Triticum_aestivum
581	2015		2015	KUMARGANJ-RECLAIMED		KUMARGANJ-RECLAIMED	3	NEPZ	NORMAL	NOT_HOSTILE	8.3	1	K_0307	19	Triticum_aestivum
582	2015		2015	KUMARGANJ-RECLAIMED		KUMARGANJ-RECLAIMED	3	NEPZ	NORMAL	NOT_HOSTILE	8.3	1	BH_1146	1	Triticum_aestivum
583	2015		2015	KUMARGANJ-RECLAIMED		KUMARGANJ-RECLAIMED	3	NEPZ	NORMAL	NOT_HOSTILE	8.3	1	RAJ_4229	31	Triticum_aestivum
584	2015		2015	KUMARGANJ-RECLAIMED		KUMARGANJ-RECLAIMED	3	NEPZ	NORMAL	NOT_HOSTILE	8.3	1	DBW_14	3	Triticum_aestivum
585	2015		2015	KUMARGANJ-RECLAIMED		KUMARGANJ-RECLAIMED	3	NEPZ	NORMAL	NOT_HOSTILE	8.3	1	DBW_46	7	Triticum_aestivum
586	2015		2015	KUMARGANJ-RECLAIMED		KUMARGANJ-RECLAIMED	3	NEPZ	NORMAL	NOT_HOSTILE	8.3	1	KHARCHIA_65	20	Triticum_aestivum
587	2015		2015	KUMARGANJ-RECLAIMED		KUMARGANJ-RECLAIMED	3	NEPZ	NORMAL	NOT_HOSTILE	8.3	1	DBW_16	4	Triticum_aestivum
588	2015		2015	KUMARGANJ-RECLAIMED		KUMARGANJ-RECLAIMED	3	NEPZ	NORMAL	NOT_HOSTILE	8.3	1	HI_8498	17	Triticum_turgidum
589	2015		2015	KUMARGANJ-RECLAIMED		KUMARGANJ-RECLAIMED	3	NEPZ	NORMAL	NOT_HOSTILE	8.3	1	MACS_6222	26	Triticum_aestivum
590	2015		2015	KUMARGANJ-RECLAIMED		KUMARGANJ-RECLAIMED	3	NEPZ	NORMAL	NOT_HOSTILE	8.3	1	DBW_71	9	Triticum_aestivum
591	2015		2015	KUMARGANJ-RECLAIMED		KUMARGANJ-RECLAIMED	3	NEPZ	NORMAL	NOT_HOSTILE	8.3	1	NW_4018	28	Triticum_aestivum
592	2015		2015	KUMARGANJ-RECLAIMED		KUMARGANJ-RECLAIMED	3	NEPZ	NORMAL	NOT_HOSTILE	8.3	1	NW_4092	29	Triticum_aestivum
593	2015		2015	KUMARGANJ-RECLAIMED		KUMARGANJ-RECLAIMED	3	NEPZ	NORMAL	NOT_HOSTILE	8.3	1	WH_1021	35	Triticum_aestivum
594	2015		2015	KUMARGANJ-RECLAIMED		KUMARGANJ-RECLAIMED	3	NEPZ	NORMAL	NOT_HOSTILE	8.3	1	KRL_19	22	Triticum_aestivum
595	2015		2015	KUMARGANJ-RECLAIMED		KUMARGANJ-RECLAIMED	3	NEPZ	NORMAL	NOT_HOSTILE	8.3	1	DBW_51	8	Triticum_aestivum
596	2015		2015	KUMARGANJ-RECLAIMED		KUMARGANJ-RECLAIMED	3	NEPZ	NORMAL	NOT_HOSTILE	8.3	1	HI_1563	16	Triticum_aestivum
597	2015		2015	KUMARGANJ-RECLAIMED		KUMARGANJ-RECLAIMED	3	NEPZ	NORMAL	NOT_HOSTILE	8.3	1	CBW_38	2	Triticum_aestivum
598	2015		2015	KUMARGANJ-RECLAIMED		KUMARGANJ-RECLAIMED	3	NEPZ	NORMAL	NOT_HOSTILE	8.3	1	GW_322	11	Triticum_aestivum
599	2015		2015	KUMARGANJ-RECLAIMED		KUMARGANJ-RECLAIMED	3	NEPZ	NORMAL	NOT_HOSTILE	8.3	1	RW_3684	33	Triticum_aestivum
600	2015		2015	KUMARGANJ-RECLAIMED		KUMARGANJ-RECLAIMED	3	NEPZ	NORMAL	NOT_HOSTILE	8.3	1	KRL_3-4	25	Triticum_aestivum
601	2015		2015	KUMARGANJ-RECLAIMED		KUMARGANJ-RECLAIMED	3	NEPZ	NORMAL	NOT_HOSTILE	8.3	1	KRL_1-4	21	Triticum_aestivum
602	2015		2015	KUMARGANJ-RECLAIMED		KUMARGANJ-RECLAIMED	3	NEPZ	NORMAL	NOT_HOSTILE	8.3	1	UP_262	34	Triticum_aestivum
603	2015		2015	KUMARGANJ-RECLAIMED		KUMARGANJ-RECLAIMED	3	NEPZ	NORMAL	NOT_HOSTILE	8.3	1	HD_2932	14	Triticum_aestivum
604	2015		2015	KUMARGANJ-RECLAIMED		KUMARGANJ-RECLAIMED	3	NEPZ	NORMAL	NOT_HOSTILE	8.3	1	HW_2044	18	Triticum_aestivum
605	2015		2015	KUMARGANJ-RECLAIMED		KUMARGANJ-RECLAIMED	3	NEPZ	NORMAL	NOT_HOSTILE	8.3	1	HD_2009	12	Triticum_aestivum
606	2015		2015	KUMARGANJ-RECLAIMED		KUMARGANJ-RECLAIMED	3	NEPZ	NORMAL	NOT_HOSTILE	8.3	1	HD_2733	13	Triticum_aestivum
607	2015		2015	KUMARGANJ-RECLAIMED		KUMARGANJ-RECLAIMED	3	NEPZ	NORMAL	NOT_HOSTILE	8.3	1	DBW_39	6	Triticum_aestivum
608	2015		2015	KUMARGANJ-RECLAIMED		KUMARGANJ-RECLAIMED	3	NEPZ	NORMAL	NOT_HOSTILE	8.3	1	NW_1067	27	Triticum_aestivum
609	2015		2015	KUMARGANJ-RECLAIMED		KUMARGANJ-RECLAIMED	3	NEPZ	NORMAL	NOT_HOSTILE	8.3	1	WH_1105	36	Triticum_aestivum
610	2015		2015	KUMARGANJ-RECLAIMED		KUMARGANJ-RECLAIMED	3	NEPZ	NORMAL	NOT_HOSTILE	8.3	1	RAJ_4238	32	Triticum_aestivum
611	2015		2015	KUMARGANJ-RECLAIMED		KUMARGANJ-RECLAIMED	3	NEPZ	NORMAL	NOT_HOSTILE	8.3	1	KRL_210	23	Triticum_aestivum
612	2015		2015	KUMARGANJ-RECLAIMED		KUMARGANJ-RECLAIMED	3	NEPZ	NORMAL	NOT_HOSTILE	8.3	1	KRL_213	24	Triticum_aestivum
613	2015		2015	KUMARGANJ-RECLAIMED		KUMARGANJ-RECLAIMED	3	NEPZ	NORMAL	NOT_HOSTILE	8.3	2	KRL_3-4	25	Triticum_aestivum
614	2015		2015	KUMARGANJ-RECLAIMED		KUMARGANJ-RECLAIMED	3	NEPZ	NORMAL	NOT_HOSTILE	8.3	2	UP_262	34	Triticum_aestivum
615	2015		2015	KUMARGANJ-RECLAIMED		KUMARGANJ-RECLAIMED	3	NEPZ	NORMAL	NOT_HOSTILE	8.3	2	KHARCHIA_65	20	Triticum_aestivum
616	2015		2015	KUMARGANJ-RECLAIMED		KUMARGANJ-RECLAIMED	3	NEPZ	NORMAL	NOT_HOSTILE	8.3	2	DBW_17	5	Triticum_aestivum
617	2015		2015	KUMARGANJ-RECLAIMED		KUMARGANJ-RECLAIMED	3	NEPZ	NORMAL	NOT_HOSTILE	8.3	2	DBW_71	9	Triticum_aestivum
618	2015		2015	KUMARGANJ-RECLAIMED		KUMARGANJ-RECLAIMED	3	NEPZ	NORMAL	NOT_HOSTILE	8.3	2	DBW_39	6	Triticum_aestivum
619	2015		2015	KUMARGANJ-RECLAIMED		KUMARGANJ-RECLAIMED	3	NEPZ	NORMAL	NOT_HOSTILE	8.3	2	WH_1021	35	Triticum_aestivum
620	2015		2015	KUMARGANJ-RECLAIMED		KUMARGANJ-RECLAIMED	3	NEPZ	NORMAL	NOT_HOSTILE	8.3	2	KRL_210	23	Triticum_aestivum
621	2015		2015	KUMARGANJ-RECLAIMED		KUMARGANJ-RECLAIMED	3	NEPZ	NORMAL	NOT_HOSTILE	8.3	2	DBW_16	4	Triticum_aestivum
622	2015		2015	KUMARGANJ-RECLAIMED		KUMARGANJ-RECLAIMED	3	NEPZ	NORMAL	NOT_HOSTILE	8.3	2	HD_2009	12	Triticum_aestivum
623	2015		2015	KUMARGANJ-RECLAIMED		KUMARGANJ-RECLAIMED	3	NEPZ	NORMAL	NOT_HOSTILE	8.3	2	CBW_38	2	Triticum_aestivum
624	2015		2015	KUMARGANJ-RECLAIMED		KUMARGANJ-RECLAIMED	3	NEPZ	NORMAL	NOT_HOSTILE	8.3	2	DPW_621-50	10	Triticum_aestivum
625	2015		2015	KUMARGANJ-RECLAIMED		KUMARGANJ-RECLAIMED	3	NEPZ	NORMAL	NOT_HOSTILE	8.3	2	DBW_46	7	Triticum_aestivum
626	2015		2015	KUMARGANJ-RECLAIMED		KUMARGANJ-RECLAIMED	3	NEPZ	NORMAL	NOT_HOSTILE	8.3	2	RAJ_4238	32	Triticum_aestivum
627	2015		2015	KUMARGANJ-RECLAIMED		KUMARGANJ-RECLAIMED	3	NEPZ	NORMAL	NOT_HOSTILE	8.3	2	NW_4092	29	Triticum_aestivum
628	2015		2015	KUMARGANJ-RECLAIMED		KUMARGANJ-RECLAIMED	3	NEPZ	NORMAL	NOT_HOSTILE	8.3	2	KRL_1-4	21	Triticum_aestivum
629	2015		2015	KUMARGANJ-RECLAIMED		KUMARGANJ-RECLAIMED	3	NEPZ	NORMAL	NOT_HOSTILE	8.3	2	K_0307	19	Triticum_aestivum
630	2015		2015	KUMARGANJ-RECLAIMED		KUMARGANJ-RECLAIMED	3	NEPZ	NORMAL	NOT_HOSTILE	8.3	2	GW_322	11	Triticum_aestivum
631	2015		2015	KUMARGANJ-RECLAIMED		KUMARGANJ-RECLAIMED	3	NEPZ	NORMAL	NOT_HOSTILE	8.3	2	DBW_51	8	Triticum_aestivum
632	2015		2015	KUMARGANJ-RECLAIMED		KUMARGANJ-RECLAIMED	3	NEPZ	NORMAL	NOT_HOSTILE	8.3	2	PDW_314	30	Triticum_turgidum
633	2015		2015	KUMARGANJ-RECLAIMED		KUMARGANJ-RECLAIMED	3	NEPZ	NORMAL	NOT_HOSTILE	8.3	2	MACS_6222	26	Triticum_aestivum
634	2015		2015	KUMARGANJ-RECLAIMED		KUMARGANJ-RECLAIMED	3	NEPZ	NORMAL	NOT_HOSTILE	8.3	2	HD_2932	14	Triticum_aestivum
635	2015		2015	KUMARGANJ-RECLAIMED		KUMARGANJ-RECLAIMED	3	NEPZ	NORMAL	NOT_HOSTILE	8.3	2	KRL_213	24	Triticum_aestivum
636	2015		2015	KUMARGANJ-RECLAIMED		KUMARGANJ-RECLAIMED	3	NEPZ	NORMAL	NOT_HOSTILE	8.3	2	RAJ_4229	31	Triticum_aestivum
637	2015		2015	KUMARGANJ-RECLAIMED		KUMARGANJ-RECLAIMED	3	NEPZ	NORMAL	NOT_HOSTILE	8.3	2	WH_1105	36	Triticum_aestivum
638	2015		2015	KUMARGANJ-RECLAIMED		KUMARGANJ-RECLAIMED	3	NEPZ	NORMAL	NOT_HOSTILE	8.3	2	HD_2967	15	Triticum_aestivum
639	2015		2015	KUMARGANJ-RECLAIMED		KUMARGANJ-RECLAIMED	3	NEPZ	NORMAL	NOT_HOSTILE	8.3	2	HD_2733	13	Triticum_aestivum
640	2015		2015	KUMARGANJ-RECLAIMED		KUMARGANJ-RECLAIMED	3	NEPZ	NORMAL	NOT_HOSTILE	8.3	2	NW_4018	28	Triticum_aestivum
641	2015		2015	KUMARGANJ-RECLAIMED		KUMARGANJ-RECLAIMED	3	NEPZ	NORMAL	NOT_HOSTILE	8.3	2	HI_1563	16	Triticum_aestivum
642	2015		2015	KUMARGANJ-RECLAIMED		KUMARGANJ-RECLAIMED	3	NEPZ	NORMAL	NOT_HOSTILE	8.3	2	DBW_14	3	Triticum_aestivum
643	2015		2015	KUMARGANJ-RECLAIMED		KUMARGANJ-RECLAIMED	3	NEPZ	NORMAL	NOT_HOSTILE	8.3	2	HI_8498	17	Triticum_turgidum
644	2015		2015	KUMARGANJ-RECLAIMED		KUMARGANJ-RECLAIMED	3	NEPZ	NORMAL	NOT_HOSTILE	8.3	2	BH_1146	1	Triticum_aestivum
645	2015		2015	KUMARGANJ-RECLAIMED		KUMARGANJ-RECLAIMED	3	NEPZ	NORMAL	NOT_HOSTILE	8.3	2	NW_1067	27	Triticum_aestivum
646	2015		2015	KUMARGANJ-RECLAIMED		KUMARGANJ-RECLAIMED	3	NEPZ	NORMAL	NOT_HOSTILE	8.3	2	RW_3684	33	Triticum_aestivum
647	2015		2015	KUMARGANJ-RECLAIMED		KUMARGANJ-RECLAIMED	3	NEPZ	NORMAL	NOT_HOSTILE	8.3	2	KRL_19	22	Triticum_aestivum
648	2015		2015	KUMARGANJ-RECLAIMED		KUMARGANJ-RECLAIMED	3	NEPZ	NORMAL	NOT_HOSTILE	8.3	2	HW_2044	18	Triticum_aestivum
649	2015		2015	KUMARGANJ-SODIC		KUMARGANJ-SODIC	4	NEPZ	SODIC	HOSTILE	9.5	1	PDW_314	30	Triticum_turgidum
650	2015		2015	KUMARGANJ-SODIC		KUMARGANJ-SODIC	4	NEPZ	SODIC	HOSTILE	9.5	1	HD_2967	15	Triticum_aestivum
651	2015		2015	KUMARGANJ-SODIC		KUMARGANJ-SODIC	4	NEPZ	SODIC	HOSTILE	9.5	1	DPW_621-50	10	Triticum_aestivum
652	2015		2015	KUMARGANJ-SODIC		KUMARGANJ-SODIC	4	NEPZ	SODIC	HOSTILE	9.5	1	DBW_17	5	Triticum_aestivum
653	2015		2015	KUMARGANJ-SODIC		KUMARGANJ-SODIC	4	NEPZ	SODIC	HOSTILE	9.5	1	K_0307	19	Triticum_aestivum
654	2015		2015	KUMARGANJ-SODIC		KUMARGANJ-SODIC	4	NEPZ	SODIC	HOSTILE	9.5	1	BH_1146	1	Triticum_aestivum
655	2015		2015	KUMARGANJ-SODIC		KUMARGANJ-SODIC	4	NEPZ	SODIC	HOSTILE	9.5	1	RAJ_4229	31	Triticum_aestivum
656	2015		2015	KUMARGANJ-SODIC		KUMARGANJ-SODIC	4	NEPZ	SODIC	HOSTILE	9.5	1	DBW_14	3	Triticum_aestivum
657	2015		2015	KUMARGANJ-SODIC		KUMARGANJ-SODIC	4	NEPZ	SODIC	HOSTILE	9.5	1	DBW_46	7	Triticum_aestivum
658	2015		2015	KUMARGANJ-SODIC		KUMARGANJ-SODIC	4	NEPZ	SODIC	HOSTILE	9.5	1	KHARCHIA_65	20	Triticum_aestivum
659	2015		2015	KUMARGANJ-SODIC		KUMARGANJ-SODIC	4	NEPZ	SODIC	HOSTILE	9.5	1	DBW_16	4	Triticum_aestivum
660	2015		2015	KUMARGANJ-SODIC		KUMARGANJ-SODIC	4	NEPZ	SODIC	HOSTILE	9.5	1	HI_8498	17	Triticum_turgidum
661	2015		2015	KUMARGANJ-SODIC		KUMARGANJ-SODIC	4	NEPZ	SODIC	HOSTILE	9.5	1	MACS_6222	26	Triticum_aestivum
662	2015		2015	KUMARGANJ-SODIC		KUMARGANJ-SODIC	4	NEPZ	SODIC	HOSTILE	9.5	1	DBW_71	9	Triticum_aestivum
663	2015		2015	KUMARGANJ-SODIC		KUMARGANJ-SODIC	4	NEPZ	SODIC	HOSTILE	9.5	1	NW_4018	28	Triticum_aestivum
664	2015		2015	KUMARGANJ-SODIC		KUMARGANJ-SODIC	4	NEPZ	SODIC	HOSTILE	9.5	1	NW_4092	29	Triticum_aestivum
665	2015		2015	KUMARGANJ-SODIC		KUMARGANJ-SODIC	4	NEPZ	SODIC	HOSTILE	9.5	1	WH_1021	35	Triticum_aestivum
666	2015		2015	KUMARGANJ-SODIC		KUMARGANJ-SODIC	4	NEPZ	SODIC	HOSTILE	9.5	1	KRL_19	22	Triticum_aestivum
667	2015		2015	KUMARGANJ-SODIC		KUMARGANJ-SODIC	4	NEPZ	SODIC	HOSTILE	9.5	1	DBW_51	8	Triticum_aestivum
668	2015		2015	KUMARGANJ-SODIC		KUMARGANJ-SODIC	4	NEPZ	SODIC	HOSTILE	9.5	1	HI_1563	16	Triticum_aestivum
669	2015		2015	KUMARGANJ-SODIC		KUMARGANJ-SODIC	4	NEPZ	SODIC	HOSTILE	9.5	1	CBW_38	2	Triticum_aestivum
670	2015		2015	KUMARGANJ-SODIC		KUMARGANJ-SODIC	4	NEPZ	SODIC	HOSTILE	9.5	1	GW_322	11	Triticum_aestivum
671	2015		2015	KUMARGANJ-SODIC		KUMARGANJ-SODIC	4	NEPZ	SODIC	HOSTILE	9.5	1	RW_3684	33	Triticum_aestivum
672	2015		2015	KUMARGANJ-SODIC		KUMARGANJ-SODIC	4	NEPZ	SODIC	HOSTILE	9.5	1	KRL_3-4	25	Triticum_aestivum
673	2015		2015	KUMARGANJ-SODIC		KUMARGANJ-SODIC	4	NEPZ	SODIC	HOSTILE	9.5	1	KRL_1-4	21	Triticum_aestivum
674	2015		2015	KUMARGANJ-SODIC		KUMARGANJ-SODIC	4	NEPZ	SODIC	HOSTILE	9.5	1	UP_262	34	Triticum_aestivum
675	2015		2015	KUMARGANJ-SODIC		KUMARGANJ-SODIC	4	NEPZ	SODIC	HOSTILE	9.5	1	HD_2932	14	Triticum_aestivum
676	2015		2015	KUMARGANJ-SODIC		KUMARGANJ-SODIC	4	NEPZ	SODIC	HOSTILE	9.5	1	HW_2044	18	Triticum_aestivum
677	2015		2015	KUMARGANJ-SODIC		KUMARGANJ-SODIC	4	NEPZ	SODIC	HOSTILE	9.5	1	HD_2009	12	Triticum_aestivum
678	2015		2015	KUMARGANJ-SODIC		KUMARGANJ-SODIC	4	NEPZ	SODIC	HOSTILE	9.5	1	HD_2733	13	Triticum_aestivum
679	2015		2015	KUMARGANJ-SODIC		KUMARGANJ-SODIC	4	NEPZ	SODIC	HOSTILE	9.5	1	DBW_39	6	Triticum_aestivum
680	2015		2015	KUMARGANJ-SODIC		KUMARGANJ-SODIC	4	NEPZ	SODIC	HOSTILE	9.5	1	NW_1067	27	Triticum_aestivum
681	2015		2015	KUMARGANJ-SODIC		KUMARGANJ-SODIC	4	NEPZ	SODIC	HOSTILE	9.5	1	WH_1105	36	Triticum_aestivum
682	2015		2015	KUMARGANJ-SODIC		KUMARGANJ-SODIC	4	NEPZ	SODIC	HOSTILE	9.5	1	RAJ_4238	32	Triticum_aestivum
683	2015		2015	KUMARGANJ-SODIC		KUMARGANJ-SODIC	4	NEPZ	SODIC	HOSTILE	9.5	1	KRL_210	23	Triticum_aestivum
684	2015		2015	KUMARGANJ-SODIC		KUMARGANJ-SODIC	4	NEPZ	SODIC	HOSTILE	9.5	1	KRL_213	24	Triticum_aestivum
685	2015		2015	KUMARGANJ-SODIC		KUMARGANJ-SODIC	4	NEPZ	SODIC	HOSTILE	9.5	2	KRL_3-4	25	Triticum_aestivum
686	2015		2015	KUMARGANJ-SODIC		KUMARGANJ-SODIC	4	NEPZ	SODIC	HOSTILE	9.5	2	UP_262	34	Triticum_aestivum
687	2015		2015	KUMARGANJ-SODIC		KUMARGANJ-SODIC	4	NEPZ	SODIC	HOSTILE	9.5	2	KHARCHIA_65	20	Triticum_aestivum
688	2015		2015	KUMARGANJ-SODIC		KUMARGANJ-SODIC	4	NEPZ	SODIC	HOSTILE	9.5	2	DBW_17	5	Triticum_aestivum
689	2015		2015	KUMARGANJ-SODIC		KUMARGANJ-SODIC	4	NEPZ	SODIC	HOSTILE	9.5	2	DBW_71	9	Triticum_aestivum
690	2015		2015	KUMARGANJ-SODIC		KUMARGANJ-SODIC	4	NEPZ	SODIC	HOSTILE	9.5	2	DBW_39	6	Triticum_aestivum
691	2015		2015	KUMARGANJ-SODIC		KUMARGANJ-SODIC	4	NEPZ	SODIC	HOSTILE	9.5	2	WH_1021	35	Triticum_aestivum
692	2015		2015	KUMARGANJ-SODIC		KUMARGANJ-SODIC	4	NEPZ	SODIC	HOSTILE	9.5	2	KRL_210	23	Triticum_aestivum
693	2015		2015	KUMARGANJ-SODIC		KUMARGANJ-SODIC	4	NEPZ	SODIC	HOSTILE	9.5	2	DBW_16	4	Triticum_aestivum
694	2015		2015	KUMARGANJ-SODIC		KUMARGANJ-SODIC	4	NEPZ	SODIC	HOSTILE	9.5	2	HD_2009	12	Triticum_aestivum
695	2015		2015	KUMARGANJ-SODIC		KUMARGANJ-SODIC	4	NEPZ	SODIC	HOSTILE	9.5	2	CBW_38	2	Triticum_aestivum
696	2015		2015	KUMARGANJ-SODIC		KUMARGANJ-SODIC	4	NEPZ	SODIC	HOSTILE	9.5	2	DPW_621-50	10	Triticum_aestivum
697	2015		2015	KUMARGANJ-SODIC		KUMARGANJ-SODIC	4	NEPZ	SODIC	HOSTILE	9.5	2	DBW_46	7	Triticum_aestivum
698	2015		2015	KUMARGANJ-SODIC		KUMARGANJ-SODIC	4	NEPZ	SODIC	HOSTILE	9.5	2	RAJ_4238	32	Triticum_aestivum
699	2015		2015	KUMARGANJ-SODIC		KUMARGANJ-SODIC	4	NEPZ	SODIC	HOSTILE	9.5	2	NW_4092	29	Triticum_aestivum
700	2015		2015	KUMARGANJ-SODIC		KUMARGANJ-SODIC	4	NEPZ	SODIC	HOSTILE	9.5	2	KRL_1-4	21	Triticum_aestivum
701	2015		2015	KUMARGANJ-SODIC		KUMARGANJ-SODIC	4	NEPZ	SODIC	HOSTILE	9.5	2	K_0307	19	Triticum_aestivum
702	2015		2015	KUMARGANJ-SODIC		KUMARGANJ-SODIC	4	NEPZ	SODIC	HOSTILE	9.5	2	GW_322	11	Triticum_aestivum
703	2015		2015	KUMARGANJ-SODIC		KUMARGANJ-SODIC	4	NEPZ	SODIC	HOSTILE	9.5	2	DBW_51	8	Triticum_aestivum
704	2015		2015	KUMARGANJ-SODIC		KUMARGANJ-SODIC	4	NEPZ	SODIC	HOSTILE	9.5	2	PDW_314	30	Triticum_turgidum
705	2015		2015	KUMARGANJ-SODIC		KUMARGANJ-SODIC	4	NEPZ	SODIC	HOSTILE	9.5	2	MACS_6222	26	Triticum_aestivum
706	2015		2015	KUMARGANJ-SODIC		KUMARGANJ-SODIC	4	NEPZ	SODIC	HOSTILE	9.5	2	HD_2932	14	Triticum_aestivum
707	2015		2015	KUMARGANJ-SODIC		KUMARGANJ-SODIC	4	NEPZ	SODIC	HOSTILE	9.5	2	KRL_213	24	Triticum_aestivum
708	2015		2015	KUMARGANJ-SODIC		KUMARGANJ-SODIC	4	NEPZ	SODIC	HOSTILE	9.5	2	RAJ_4229	31	Triticum_aestivum
709	2015		2015	KUMARGANJ-SODIC		KUMARGANJ-SODIC	4	NEPZ	SODIC	HOSTILE	9.5	2	WH_1105	36	Triticum_aestivum
710	2015		2015	KUMARGANJ-SODIC		KUMARGANJ-SODIC	4	NEPZ	SODIC	HOSTILE	9.5	2	HD_2967	15	Triticum_aestivum
711	2015		2015	KUMARGANJ-SODIC		KUMARGANJ-SODIC	4	NEPZ	SODIC	HOSTILE	9.5	2	HD_2733	13	Triticum_aestivum
712	2015		2015	KUMARGANJ-SODIC		KUMARGANJ-SODIC	4	NEPZ	SODIC	HOSTILE	9.5	2	NW_4018	28	Triticum_aestivum
713	2015		2015	KUMARGANJ-SODIC		KUMARGANJ-SODIC	4	NEPZ	SODIC	HOSTILE	9.5	2	HI_1563	16	Triticum_aestivum
714	2015		2015	KUMARGANJ-SODIC		KUMARGANJ-SODIC	4	NEPZ	SODIC	HOSTILE	9.5	2	DBW_14	3	Triticum_aestivum
715	2015		2015	KUMARGANJ-SODIC		KUMARGANJ-SODIC	4	NEPZ	SODIC	HOSTILE	9.5	2	HI_8498	17	Triticum_turgidum
716	2015		2015	KUMARGANJ-SODIC		KUMARGANJ-SODIC	4	NEPZ	SODIC	HOSTILE	9.5	2	BH_1146	1	Triticum_aestivum
717	2015		2015	KUMARGANJ-SODIC		KUMARGANJ-SODIC	4	NEPZ	SODIC	HOSTILE	9.5	2	NW_1067	27	Triticum_aestivum
718	2015		2015	KUMARGANJ-SODIC		KUMARGANJ-SODIC	4	NEPZ	SODIC	HOSTILE	9.5	2	RW_3684	33	Triticum_aestivum
719	2015		2015	KUMARGANJ-SODIC		KUMARGANJ-SODIC	4	NEPZ	SODIC	HOSTILE	9.5	2	KRL_19	22	Triticum_aestivum
720	2015		2015	KUMARGANJ-SODIC		KUMARGANJ-SODIC	4	NEPZ	SODIC	HOSTILE	9.5	2	HW_2044	18	Triticum_aestivum
721	2015		2015	PUNDIBARI		PUNDIBARI	6	NEPZ	ACIDIC	HOSTILE	5.3	1	RW_3684	33	Triticum_aestivum
722	2015		2015	PUNDIBARI		PUNDIBARI	6	NEPZ	ACIDIC	HOSTILE	5.3	1	DBW_14	3	Triticum_aestivum
723	2015		2015	PUNDIBARI		PUNDIBARI	6	NEPZ	ACIDIC	HOSTILE	5.3	1	KRL_1-4	21	Triticum_aestivum
724	2015		2015	PUNDIBARI		PUNDIBARI	6	NEPZ	ACIDIC	HOSTILE	5.3	1	HW_2044	18	Triticum_aestivum
725	2015		2015	PUNDIBARI		PUNDIBARI	6	NEPZ	ACIDIC	HOSTILE	5.3	1	PDW_314	30	Triticum_turgidum
726	2015		2015	PUNDIBARI		PUNDIBARI	6	NEPZ	ACIDIC	HOSTILE	5.3	1	KRL_213	24	Triticum_aestivum
727	2015		2015	PUNDIBARI		PUNDIBARI	6	NEPZ	ACIDIC	HOSTILE	5.3	1	HI_8498	17	Triticum_turgidum
728	2015		2015	PUNDIBARI		PUNDIBARI	6	NEPZ	ACIDIC	HOSTILE	5.3	1	GW_322	11	Triticum_aestivum
729	2015		2015	PUNDIBARI		PUNDIBARI	6	NEPZ	ACIDIC	HOSTILE	5.3	1	DBW_71	9	Triticum_aestivum
730	2015		2015	PUNDIBARI		PUNDIBARI	6	NEPZ	ACIDIC	HOSTILE	5.3	1	WH_1105	36	Triticum_aestivum
731	2015		2015	PUNDIBARI		PUNDIBARI	6	NEPZ	ACIDIC	HOSTILE	5.3	1	RAJ_4229	31	Triticum_aestivum
732	2015		2015	PUNDIBARI		PUNDIBARI	6	NEPZ	ACIDIC	HOSTILE	5.3	1	BH_1146	1	Triticum_aestivum
733	2015		2015	PUNDIBARI		PUNDIBARI	6	NEPZ	ACIDIC	HOSTILE	5.3	1	HD_2009	12	Triticum_aestivum
734	2015		2015	PUNDIBARI		PUNDIBARI	6	NEPZ	ACIDIC	HOSTILE	5.3	1	DBW_17	5	Triticum_aestivum
735	2015		2015	PUNDIBARI		PUNDIBARI	6	NEPZ	ACIDIC	HOSTILE	5.3	1	WH_1021	35	Triticum_aestivum
736	2015		2015	PUNDIBARI		PUNDIBARI	6	NEPZ	ACIDIC	HOSTILE	5.3	1	UP_262	34	Triticum_aestivum
737	2015		2015	PUNDIBARI		PUNDIBARI	6	NEPZ	ACIDIC	HOSTILE	5.3	1	HD_2967	15	Triticum_aestivum
738	2015		2015	PUNDIBARI		PUNDIBARI	6	NEPZ	ACIDIC	HOSTILE	5.3	1	DBW_16	4	Triticum_aestivum
739	2015		2015	PUNDIBARI		PUNDIBARI	6	NEPZ	ACIDIC	HOSTILE	5.3	1	NW_1067	27	Triticum_aestivum
740	2015		2015	PUNDIBARI		PUNDIBARI	6	NEPZ	ACIDIC	HOSTILE	5.3	1	HI_1563	16	Triticum_aestivum
741	2015		2015	PUNDIBARI		PUNDIBARI	6	NEPZ	ACIDIC	HOSTILE	5.3	1	KRL_3-4	25	Triticum_aestivum
742	2015		2015	PUNDIBARI		PUNDIBARI	6	NEPZ	ACIDIC	HOSTILE	5.3	1	DBW_46	7	Triticum_aestivum
743	2015		2015	PUNDIBARI		PUNDIBARI	6	NEPZ	ACIDIC	HOSTILE	5.3	1	NW_4092	29	Triticum_aestivum
744	2015		2015	PUNDIBARI		PUNDIBARI	6	NEPZ	ACIDIC	HOSTILE	5.3	1	HD_2733	13	Triticum_aestivum
745	2015		2015	PUNDIBARI		PUNDIBARI	6	NEPZ	ACIDIC	HOSTILE	5.3	1	KRL_210	23	Triticum_aestivum
746	2015		2015	PUNDIBARI		PUNDIBARI	6	NEPZ	ACIDIC	HOSTILE	5.3	1	NW_4018	28	Triticum_aestivum
747	2015		2015	PUNDIBARI		PUNDIBARI	6	NEPZ	ACIDIC	HOSTILE	5.3	1	RAJ_4238	32	Triticum_aestivum
748	2015		2015	PUNDIBARI		PUNDIBARI	6	NEPZ	ACIDIC	HOSTILE	5.3	1	KHARCHIA_65	20	Triticum_aestivum
749	2015		2015	PUNDIBARI		PUNDIBARI	6	NEPZ	ACIDIC	HOSTILE	5.3	1	CBW_38	2	Triticum_aestivum
750	2015		2015	PUNDIBARI		PUNDIBARI	6	NEPZ	ACIDIC	HOSTILE	5.3	1	KRL_19	22	Triticum_aestivum
751	2015		2015	PUNDIBARI		PUNDIBARI	6	NEPZ	ACIDIC	HOSTILE	5.3	1	DBW_51	8	Triticum_aestivum
752	2015		2015	PUNDIBARI		PUNDIBARI	6	NEPZ	ACIDIC	HOSTILE	5.3	1	K_0307	19	Triticum_aestivum
753	2015		2015	PUNDIBARI		PUNDIBARI	6	NEPZ	ACIDIC	HOSTILE	5.3	1	DPW_621-50	10	Triticum_aestivum
754	2015		2015	PUNDIBARI		PUNDIBARI	6	NEPZ	ACIDIC	HOSTILE	5.3	1	HD_2932	14	Triticum_aestivum
755	2015		2015	PUNDIBARI		PUNDIBARI	6	NEPZ	ACIDIC	HOSTILE	5.3	1	MACS_6222	26	Triticum_aestivum
756	2015		2015	PUNDIBARI		PUNDIBARI	6	NEPZ	ACIDIC	HOSTILE	5.3	1	DBW_39	6	Triticum_aestivum
757	2015		2015	PUNDIBARI		PUNDIBARI	6	NEPZ	ACIDIC	HOSTILE	5.3	2	MACS_6222	26	Triticum_aestivum
758	2015		2015	PUNDIBARI		PUNDIBARI	6	NEPZ	ACIDIC	HOSTILE	5.3	2	WH_1021	35	Triticum_aestivum
759	2015		2015	PUNDIBARI		PUNDIBARI	6	NEPZ	ACIDIC	HOSTILE	5.3	2	HD_2733	13	Triticum_aestivum
760	2015		2015	PUNDIBARI		PUNDIBARI	6	NEPZ	ACIDIC	HOSTILE	5.3	2	HW_2044	18	Triticum_aestivum
761	2015		2015	PUNDIBARI		PUNDIBARI	6	NEPZ	ACIDIC	HOSTILE	5.3	2	BH_1146	1	Triticum_aestivum
762	2015		2015	PUNDIBARI		PUNDIBARI	6	NEPZ	ACIDIC	HOSTILE	5.3	2	KRL_19	22	Triticum_aestivum
763	2015		2015	PUNDIBARI		PUNDIBARI	6	NEPZ	ACIDIC	HOSTILE	5.3	2	DBW_71	9	Triticum_aestivum
764	2015		2015	PUNDIBARI		PUNDIBARI	6	NEPZ	ACIDIC	HOSTILE	5.3	2	DPW_621-50	10	Triticum_aestivum
765	2015		2015	PUNDIBARI		PUNDIBARI	6	NEPZ	ACIDIC	HOSTILE	5.3	2	HD_2967	15	Triticum_aestivum
766	2015		2015	PUNDIBARI		PUNDIBARI	6	NEPZ	ACIDIC	HOSTILE	5.3	2	HI_1563	16	Triticum_aestivum
767	2015		2015	PUNDIBARI		PUNDIBARI	6	NEPZ	ACIDIC	HOSTILE	5.3	2	NW_4018	28	Triticum_aestivum
768	2015		2015	PUNDIBARI		PUNDIBARI	6	NEPZ	ACIDIC	HOSTILE	5.3	2	KRL_213	24	Triticum_aestivum
769	2015		2015	PUNDIBARI		PUNDIBARI	6	NEPZ	ACIDIC	HOSTILE	5.3	2	KHARCHIA_65	20	Triticum_aestivum
770	2015		2015	PUNDIBARI		PUNDIBARI	6	NEPZ	ACIDIC	HOSTILE	5.3	2	RW_3684	33	Triticum_aestivum
771	2015		2015	PUNDIBARI		PUNDIBARI	6	NEPZ	ACIDIC	HOSTILE	5.3	2	NW_4092	29	Triticum_aestivum
772	2015		2015	PUNDIBARI		PUNDIBARI	6	NEPZ	ACIDIC	HOSTILE	5.3	2	GW_322	11	Triticum_aestivum
773	2015		2015	PUNDIBARI		PUNDIBARI	6	NEPZ	ACIDIC	HOSTILE	5.3	2	UP_262	34	Triticum_aestivum
774	2015		2015	PUNDIBARI		PUNDIBARI	6	NEPZ	ACIDIC	HOSTILE	5.3	2	DBW_51	8	Triticum_aestivum
775	2015		2015	PUNDIBARI		PUNDIBARI	6	NEPZ	ACIDIC	HOSTILE	5.3	2	RAJ_4238	32	Triticum_aestivum
776	2015		2015	PUNDIBARI		PUNDIBARI	6	NEPZ	ACIDIC	HOSTILE	5.3	2	DBW_17	5	Triticum_aestivum
777	2015		2015	PUNDIBARI		PUNDIBARI	6	NEPZ	ACIDIC	HOSTILE	5.3	2	KRL_1-4	21	Triticum_aestivum
778	2015		2015	PUNDIBARI		PUNDIBARI	6	NEPZ	ACIDIC	HOSTILE	5.3	2	WH_1105	36	Triticum_aestivum
779	2015		2015	PUNDIBARI		PUNDIBARI	6	NEPZ	ACIDIC	HOSTILE	5.3	2	NW_1067	27	Triticum_aestivum
780	2015		2015	PUNDIBARI		PUNDIBARI	6	NEPZ	ACIDIC	HOSTILE	5.3	2	DBW_39	6	Triticum_aestivum
781	2015		2015	PUNDIBARI		PUNDIBARI	6	NEPZ	ACIDIC	HOSTILE	5.3	2	HD_2009	12	Triticum_aestivum
782	2015		2015	PUNDIBARI		PUNDIBARI	6	NEPZ	ACIDIC	HOSTILE	5.3	2	K_0307	19	Triticum_aestivum
783	2015		2015	PUNDIBARI		PUNDIBARI	6	NEPZ	ACIDIC	HOSTILE	5.3	2	KRL_3-4	25	Triticum_aestivum
784	2015		2015	PUNDIBARI		PUNDIBARI	6	NEPZ	ACIDIC	HOSTILE	5.3	2	PDW_314	30	Triticum_turgidum
785	2015		2015	PUNDIBARI		PUNDIBARI	6	NEPZ	ACIDIC	HOSTILE	5.3	2	KRL_210	23	Triticum_aestivum
786	2015		2015	PUNDIBARI		PUNDIBARI	6	NEPZ	ACIDIC	HOSTILE	5.3	2	HI_8498	17	Triticum_turgidum
787	2015		2015	PUNDIBARI		PUNDIBARI	6	NEPZ	ACIDIC	HOSTILE	5.3	2	DBW_46	7	Triticum_aestivum
788	2015		2015	PUNDIBARI		PUNDIBARI	6	NEPZ	ACIDIC	HOSTILE	5.3	2	RAJ_4229	31	Triticum_aestivum
789	2015		2015	PUNDIBARI		PUNDIBARI	6	NEPZ	ACIDIC	HOSTILE	5.3	2	CBW_38	2	Triticum_aestivum
790	2015		2015	PUNDIBARI		PUNDIBARI	6	NEPZ	ACIDIC	HOSTILE	5.3	2	DBW_14	3	Triticum_aestivum
791	2015		2015	PUNDIBARI		PUNDIBARI	6	NEPZ	ACIDIC	HOSTILE	5.3	2	HD_2932	14	Triticum_aestivum
792	2015		2015	PUNDIBARI		PUNDIBARI	6	NEPZ	ACIDIC	HOSTILE	5.3	2	DBW_16	4	Triticum_aestivum
793	2015		2015	MALDA		MALDA	5	NEPZ	NORMAL	NOT_HOSTILE	7.7	1	RW_3684	33	Triticum_aestivum
794	2015		2015	MALDA		MALDA	5	NEPZ	NORMAL	NOT_HOSTILE	7.7	1	DBW_14	3	Triticum_aestivum
795	2015		2015	MALDA		MALDA	5	NEPZ	NORMAL	NOT_HOSTILE	7.7	1	KRL_1-4	21	Triticum_aestivum
796	2015		2015	MALDA		MALDA	5	NEPZ	NORMAL	NOT_HOSTILE	7.7	1	HW_2044	18	Triticum_aestivum
797	2015		2015	MALDA		MALDA	5	NEPZ	NORMAL	NOT_HOSTILE	7.7	1	PDW_314	30	Triticum_turgidum
798	2015		2015	MALDA		MALDA	5	NEPZ	NORMAL	NOT_HOSTILE	7.7	1	KRL_213	24	Triticum_aestivum
799	2015		2015	MALDA		MALDA	5	NEPZ	NORMAL	NOT_HOSTILE	7.7	1	HI_8498	17	Triticum_turgidum
800	2015		2015	MALDA		MALDA	5	NEPZ	NORMAL	NOT_HOSTILE	7.7	1	GW_322	11	Triticum_aestivum
801	2015		2015	MALDA		MALDA	5	NEPZ	NORMAL	NOT_HOSTILE	7.7	1	DBW_71	9	Triticum_aestivum
802	2015		2015	MALDA		MALDA	5	NEPZ	NORMAL	NOT_HOSTILE	7.7	1	WH_1105	36	Triticum_aestivum
803	2015		2015	MALDA		MALDA	5	NEPZ	NORMAL	NOT_HOSTILE	7.7	1	RAJ_4229	31	Triticum_aestivum
804	2015		2015	MALDA		MALDA	5	NEPZ	NORMAL	NOT_HOSTILE	7.7	1	BH_1146	1	Triticum_aestivum
805	2015		2015	MALDA		MALDA	5	NEPZ	NORMAL	NOT_HOSTILE	7.7	1	HD_2009	12	Triticum_aestivum
806	2015		2015	MALDA		MALDA	5	NEPZ	NORMAL	NOT_HOSTILE	7.7	1	DBW_17	5	Triticum_aestivum
807	2015		2015	MALDA		MALDA	5	NEPZ	NORMAL	NOT_HOSTILE	7.7	1	WH_1021	35	Triticum_aestivum
808	2015		2015	MALDA		MALDA	5	NEPZ	NORMAL	NOT_HOSTILE	7.7	1	UP_262	34	Triticum_aestivum
809	2015		2015	MALDA		MALDA	5	NEPZ	NORMAL	NOT_HOSTILE	7.7	1	HD_2967	15	Triticum_aestivum
810	2015		2015	MALDA		MALDA	5	NEPZ	NORMAL	NOT_HOSTILE	7.7	1	DBW_16	4	Triticum_aestivum
811	2015		2015	MALDA		MALDA	5	NEPZ	NORMAL	NOT_HOSTILE	7.7	1	NW_1067	27	Triticum_aestivum

Samples!	Plot_No!	TGW	GYD_t/ha	B	Na	Mg	P	S	K	Ca	Ti	Li	Be	Al	V	Cr	Mn	Fe	Co	Ni	Cu	Zn	As	Se	Rb	Sr	Mo	Ag	Cd	Cs	Ba	Tl	Pb	U
405	45	44.3	3.8	1.346226465	49.53407069	1196.936936	3265.027595	1273.105027	3768.942764	350.5153499	52.57059352	0.004943965	0.000391782	6.995184247	0.005652759	1.074675385	39.77998867	35.66385845	0.027737159	0.516801721	3.736578851	31.14091034	0.008970162	0.080280921	9.937028647	1.589704204	0.330294025	0.006342015	0.017057123	0.0122879	5.709420627	0.000522746	0.032992059	0.002621754
406	46	45.0	3.7	1.346226465	49.53407069	1165.532223	2984.662344	1304.436819	3284.099128	394.0774919	52.57059352	0.004943965	0.000391782	6.995184247	0.005652759	1.074675385	35.99208159	42.28052481	0.027737159	0.516801721	4.616369015	23.77837687	0.002818625	0.049729341	7.4948316	1.864826464	0.204183911	0.006342015	0.040241833	0.009851856	6.903920717	0.000522746	0.032992059	0.002621754
407	47	37.3	2.8	1.346226465	49.53407069	950.4877691	3076.782558	1183.096923	3517.559155	261.0192995	52.57059352	0.004943965	0.000391782	6.995184247	0.005652759	1.074675385	34.93928702	31.83972208	0.027737159	0.516801721	3.449962211	21.20655181	0.002818625	0.068074241	8.398062781	1.206515499	0.360437698	0.006342015	0.017057123	0.00694108	3.954207617	0.000522746	0.032992059	0.002621754
408	48	36.0	3.86	1.346226465	49.53407069	947.2691617	3193.229595	1204.070292	3919.777172	299.5540135	52.57059352	0.004943965	0.000391782	6.995184247	0.005652759	1.074675385	34.16399863	30.9111227	0.027737159	0.516801721	3.492116115	22.67617237	0.002818625	0.059180507	7.454750839	1.608537982	0.273411264	0.016040998	0.017057123	0.005088405	5.664067076	0.000522746	0.032992059	0.002621754
409	49	37.4	1.08	1.346226465	49.53407069	1214.844391	3168.73113	1346.884262	4449.412817	356.7546377	52.57059352	0.004943965	0.000391782	6.995184247	0.005652759	1.074675385	38.28308728	43.84626059	0.027737159	0.516801721	4.11549715	23.11741393	0.002818625	0.042353545	11.13869122	1.373376756	0.179377673	0.006342015	0.039138512	0.011250676	4.495334086	0.000522746	0.032992059	0.002621754
410	50	35.7	2.68	1.346226465	49.53407069	851.2288645	2907.719178	1184.363554	3791.999923	262.6681184	52.57059352	0.004943965	0.000391782	6.995184247	0.005652759	1.074675385	40.09418459	36.84709396	0.027737159	0.516801721	3.110910777	19.87026592	0.002818625	0.05683191	10.91507577	1.333306049	0.208562506	0.006342015	0.017057123	0.01178555	3.457824435	0.000522746	0.032992059	0.002621754
411	51	35.1	3.16	1.346226465	49.53407069	1132.102747	3737.981282	1432.793994	4142.354692	333.460054	52.57059352	0.004943965	0.000391782	6.995184247	0.005652759	1.074675385	35.47152457	35.99517023	0.027737159	0.516801721	3.956085594	26.53043996	0.012870481	0.075206754	11.30117059	1.488621306	0.247177642	0.006342015	0.017057123	0.013019231	4.141817298	0.000522746	0.032992059	0.002621754
412	52	39.4	3.46	1.346226465	49.53407069	1006.255443	2855.932252	1188.2294	3784.464115	335.9325306	52.57059352	0.004943965	0.000391782	6.995184247	0.005652759	1.074675385	35.71565032	34.15836176	0.027737159	0.516801721	3.998843332	24.2364279	0.002818625	0.043538287	9.594793512	1.545182035	0.264905143	0.006342015	0.017057123	0.010693063	4.900715495	0.000522746	0.032992059	0.002621754
413	53	41.2	2.8	1.346226465	49.53407069	1270.104522	3463.985338	1413.380408	3936.70526	355.3491144	52.57059352	0.004943965	0.000391782	6.995184247	0.005652759	1.074675385	42.48114603	37.15873912	0.027737159	0.516801721	4.344385726	25.78398483	0.011423573	0.057077859	8.869776544	1.393270034	0.303136188	0.006342015	0.017057123	0.013483534	4.020613409	0.000522746	0.032992059	0.002621754
414	54	37.9	3.48	1.346226465	49.53407069	972.2716759	3147.779812	1025.516253	3412.149013	299.3895306	52.57059352	0.004943965	0.000391782	6.995184247	0.005652759	1.074675385	33.32018094	28.85225913	0.027737159	0.516801721	3.024336816	22.33096481	0.002818625	0.063403875	6.432173667	1.468697347	0.234988769	0.006342015	0.017057123	0.00563549	5.481196559	0.000522746	0.032992059	0.002621754
415	55	42.2	3.36	1.346226465	49.53407069	1167.783068	3280.290881	1297.172372	4043.269639	298.0118376	52.57059352	0.004943965	0.000391782	6.995184247	0.005652759	1.074675385	33.6164625	38.56144075	0.027737159	0.516801721	3.637612058	22.01655776	0.011234406	0.060111168	7.848807534	1.343392019	0.243503352	0.006342015	0.043432875	0.009476548	5.204284951	0.000522746	0.032992059	0.002621754
416	56	37.9	3.3	1.346226465	49.53407069	1277.164179	4547.169209	1503.852524	4552.429574	263.2542641	52.57059352	0.004943965	0.000391782	6.995184247	0.005652759	1.074675385	44.90488962	38.52077523	0.027737159	0.516801721	3.647618115	33.65984934	0.002818625	0.049077506	10.94450356	1.630680639	0.228638544	0.006342015	0.04447965	0.012066627	5.470976134	0.000522746	0.032992059	0.002621754
417	57	46.7	3.36	1.346226465	49.53407069	1253.065483	3326.445247	1571.730261	3996.680705	312.7960171	52.57059352	0.004943965	0.000391782	6.995184247	0.005652759	1.074675385	38.5811878	23.39135008	0.027737159	0.516801721	3.038217344	28.48961731	0.01617436	0.038205484	9.87525658	1.200597538	0.338388505	0.006342015	0.045536554	0.015717923	3.58071368	0.000522746	0.032992059	0.002621754
418	58	35.4	3.16	1.346226465	49.53407069	1128.675982	2976.418253	1331.959585	3627.0827	381.8812322	52.57059352	0.004943965	0.000391782	6.995184247	0.005652759	1.074675385	37.6734404	31.01254728	0.027737159	0.516801721	4.282382657	24.13690255	0.002818625	0.072337432	9.416955576	1.705590505	0.275017384	0.006342015	0.017057123	0.013399015	4.157897693	0.000522746	0.032992059	0.002621754
419	59	35.5	2.88	1.346226465	49.53407069	967.7732402	3136.587313	1253.704963	4371.681629	383.1773041	52.57059352	0.004943965	0.000391782	6.995184247	0.005652759	1.074675385	35.22220217	34.29237398	0.027737159	0.516801721	3.575943326	19.50328071	0.002818625	0.074923548	8.059575318	1.693071823	0.222485399	0.006342015	0.017057123	0.005725427	4.985234079	0.000522746	0.032992059	0.002621754
420	60	49.6	3.76	1.346226465	49.53407069	1215.427632	3632.592911	1270.620637	4113.958032	251.8814922	52.57059352	0.004943965	0.000391782	6.995184247	0.005652759	1.074675385	38.2234798	33.54960489	0.027737159	0.516801721	3.631192434	32.58331917	0.008083965	0.118939872	6.824306107	1.250406367	0.422766072	0.013119812	0.017057123	0.005252764	4.754088287	0.000522746	0.032992059	0.002621754
421	61	41.9	3.4	1.346226465	49.53407069	1064.874258	3074.166924	1389.033218	3384.543739	257.8446143	52.57059352	0.004943965	0.000391782	6.995184247	0.005652759	1.074675385	34.39407955	36.22204462	0.027737159	0.516801721	3.494295797	20.51083234	0.012017789	0.075491854	9.820596794	1.212615367	0.215638279	0.006342015	0.017057123	0.013721659	2.163648772	0.000522746	0.032992059	0.002621754
422	62	41.0	2.9	1.346226465	49.53407069	1418.268716	4256.241545	1556.059777	4849.686431	342.6957034	52.57059352	0.004943965	0.000391782	6.995184247	0.005652759	1.074675385	50.52284822	41.46853908	0.027737159	0.516801721	4.402018623	38.92123215	0.002818625	0.083136754	12.03681607	1.635124854	0.325330821	0.006342015	0.039745536	0.01689793	4.315743022	0.000522746	0.032992059	0.002621754
423	63	33.9	1.52	1.346226465	49.53407069	1450.252023	4577.964295	1646.29013	4294.350525	375.8183386	52.57059352	0.004943965	0.000391782	6.995184247	0.005652759	1.074675385	46.0500176	44.06524777	0.027737159	0.516801721	3.625384906	39.02010668	0.007470384	0.088408515	15.74152203	1.519560122	0.279817433	0.006342015	0.062436064	0.019829571	3.36951677	0.000522746	0.032992059	0.002621754
424	64	46.1	2.48	1.346226465	49.53407069	958.0117971	3357.129956	1210.753983	3768.977942	307.0176379	52.57059352	0.004943965	0.000391782	6.995184247	0.005652759	1.074675385	26.42616976	29.72029377	0.027737159	0.516801721	4.395637985	28.41452172	0.007051216	0.108599458	9.685096866	0.551870877	0.37470532	0.006342015	0.085156364	0.014679659	2.135320149	0.000522746	0.032992059	0.002621754
425	65	32.9	3.32	1.346226465	49.53407069	1042.123414	2994.505093	1155.542509	3647.871998	310.4493428	52.57059352	0.004943965	0.000391782	6.995184247	0.005652759	1.074675385	42.93640554	33.93339778	0.027737159	0.516801721	3.643023424	23.14760618	0.002818625	0.059800981	9.355316118	1.630228471	0.24664797	0.015775829	0.017057123	0.009774293	4.445294303	0.000522746	0.032992059	0.002621754
426	66	48.4	2.42	1.346226465	49.53407069	910.041882	3252.360052	1022.374091	3888.968034	235.4916564	52.57059352	0.004943965	0.000391782	6.995184247	0.005652759	1.074675385	27.07488328	29.32457852	0.027737159	0.516801721	4.050041971	27.25681771	0.002818625	0.100087507	7.536468491	0.463369544	0.242558717	0.006342015	0.017057123	0.010007061	2.397146139	0.000522746	0.032992059	0.002621754
427	67	45.1	4.14	1.346226465	49.53407069	1036.545616	3284.321804	1108.217524	3508.76029	350.7340059	52.57059352	0.004943965	0.000391782	6.995184247	0.005652759	1.074675385	32.32035751	31.71258565	0.027737159	0.516801721	2.697399025	22.16299009	0.010718238	0.060661303	8.315588427	1.374932857	0.173891898	0.006342015	0.017057123	0.00881727	4.057932041	0.000522746	0.032992059	0.002621754
428	68	38.1	3.6	1.346226465	49.53407069	1045.587364	2885.185523	1261.393164	3901.838432	288.7078737	52.57059352	0.004943965	0.000391782	6.995184247	0.005652759	1.074675385	29.3314528	34.70397291	0.027737159	0.516801721	3.742955276	18.50967569	0.014306762	0.056633528	6.841259565	1.082262648	0.191238213	0.006342015	0.017057123	0.007766393	4.978553374	0.000522746	0.032992059	0.002621754
429	69	39.3	3.68	1.346226465	49.53407069	958.1290476	2872.815317	997.1880109	3453.776578	230.3676774	52.57059352	0.004943965	0.000391782	6.995184247	0.005652759	1.074675385	37.07315977	29.82917387	0.027737159	0.516801721	3.213692196	22.03858869	0.006627747	0.073592785	8.258772814	0.987790546	0.355603474	0.006342015	0.017057123	0.007714388	4.080187407	0.000522746	0.032992059	0.002621754
430	70	44.6	3.16	1.346226465	49.53407069	909.3893743	2829.493017	1008.639478	3048.053807	213.5928178	52.57059352	0.004943965	0.000391782	6.995184247	0.005652759	1.074675385	33.4275652	28.07504412	0.027737159	0.516801721	3.28014563	23.31507451	0.002818625	0.079492562	6.989255746	0.980813843	0.2809749	0.006342015	0.017057123	0.008754556	4.458233871	0.000522746	0.032992059	0.002621754
431	71	40.1	3.2	1.346226465	49.53407069	1048.573562	3005.039776	1266.50208	3939.590196	384.4513516	52.57059352	0.004943965	0.000391782	6.995184247	0.005652759	1.074675385	43.00588624	32.72411087	0.027737159	0.516801721	3.684274567	22.01136106	0.002818625	0.063604135	8.153068197	1.412527239	0.245985471	0.006342015	0.036671104	0.009369042	5.491434916	0.000522746	0.032992059	0.002621754
432	72	37.8	3.44	1.346226465	49.53407069	392.1943426	1282.732262	412.9787826	1556.079138	93.31501484	52.57059352	0.004943965	0.000391782	6.995184247	0.005652759	1.074675385	12.64165614	6.427420167	0.027737159	0.516801721	0.651169357	10.19900738	0.002818625	0.014843469	2.572803976	0.304696326	0.055700032	0.006342015	0.017057123	0.001235517	1.436995796	0.000522746	0.032992059	0.002621754

Samples!	Year!	Year1!	Year2!	Site_Name!	Site2014!	site2015!	Site Ranking!	ZONE!	Soil!	HOSTILE_NOT!	SOIL_pH!	Replication!	Genotype!	Genotype Rank!	Species!	Plot_No!	TGW	GYD_t/ha
812	2015	2015	2015	MALDA		MALDA	5	NEPZ	NORMAL	NOT_HOSTILE	7.7	1	HI_1563	16	Triticum_aestivum	20	34.2	5.2
813	2015	2015	2015	MALDA		MALDA	5	NEPZ	NORMAL	NOT_HOSTILE	7.7	1	KRL_3-4	25	Triticum_aestivum	21	22.2	1.62
814	2015	2015	2015	MALDA		MALDA	5	NEPZ	NORMAL	NOT_HOSTILE	7.7	1	DBW_46	7	Triticum_aestivum	22	37.1	3.64
815	2015	2015	2015	MALDA		MALDA	5	NEPZ	NORMAL	NOT_HOSTILE	7.7	1	NW_4092	29	Triticum_aestivum	23	40.3	5.32
816	2015	2015	2015	MALDA		MALDA	5	NEPZ	NORMAL	NOT_HOSTILE	7.7	1	HD_2733	13	Triticum_aestivum	24	46.2	4.7
817	2015	2015	2015	MALDA		MALDA	5	NEPZ	NORMAL	NOT_HOSTILE	7.7	1	KRL_210	23	Triticum_aestivum	25	36.7	4.46
818	2015	2015	2015	MALDA		MALDA	5	NEPZ	NORMAL	NOT_HOSTILE	7.7	1	NW_4018	28	Triticum_aestivum	26	39.4	4.02
819	2015	2015	2015	MALDA		MALDA	5	NEPZ	NORMAL	NOT_HOSTILE	7.7	1	RAJ_4238	32	Triticum_aestivum	27	33.0	4.78
820	2015	2015	2015	MALDA		MALDA	5	NEPZ	NORMAL	NOT_HOSTILE	7.7	1	KHARCHIA_65	20	Triticum_aestivum	28	31.5	2.58
821	2015	2015	2015	MALDA		MALDA	5	NEPZ	NORMAL	NOT_HOSTILE	7.7	1	CBW_38	2	Triticum_aestivum	29	37.5	4.86
822	2015	2015	2015	MALDA		MALDA	5	NEPZ	NORMAL	NOT_HOSTILE	7.7	1	KRL_19	22	Triticum_aestivum	30	29.4	4.06
823	2015	2015	2015	MALDA		MALDA	5	NEPZ	NORMAL	NOT_HOSTILE	7.7	1	DBW_51	8	Triticum_aestivum	31	38.4	4.34
824	2015	2015	2015	MALDA		MALDA	5	NEPZ	NORMAL	NOT_HOSTILE	7.7	1	K_0307	19	Triticum_aestivum	32	38.6	4.8
825	2015	2015	2015	MALDA		MALDA	5	NEPZ	NORMAL	NOT_HOSTILE	7.7	1	DPW_621-50	10	Triticum_aestivum	33	44.9	2.94
826	2015	2015	2015	MALDA		MALDA	5	NEPZ	NORMAL	NOT_HOSTILE	7.7	1	HD_2932	14	Triticum_aestivum	34	33.1	4.56
827	2015	2015	2015	MALDA		MALDA	5	NEPZ	NORMAL	NOT_HOSTILE	7.7	1	MACS_6222	26	Triticum_aestivum	35	34.8	4.88
828	2015	2015	2015	MALDA		MALDA	5	NEPZ	NORMAL	NOT_HOSTILE	7.7	1	DBW_39	6	Triticum_aestivum	36	45.8	4.82
829	2015	2015	2015	MALDA		MALDA	5	NEPZ	NORMAL	NOT_HOSTILE	7.7	2	MACS_6222	26	Triticum_aestivum	37	38.4	4.68
830	2015	2015	2015	MALDA		MALDA	5	NEPZ	NORMAL	NOT_HOSTILE	7.7	2	WH_1021	35	Triticum_aestivum	38	41.2	5.3
831	2015	2015	2015	MALDA		MALDA	5	NEPZ	NORMAL	NOT_HOSTILE	7.7	2	HD_2733	13	Triticum_aestivum	39	46.0	4.1
832	2015	2015	2015	MALDA		MALDA	5	NEPZ	NORMAL	NOT_HOSTILE	7.7	2	HW_2044	18	Triticum_aestivum	40	34.6	4.46
833	2015	2015	2015	MALDA		MALDA	5	NEPZ	NORMAL	NOT_HOSTILE	7.7	2	BH_1146	1	Triticum_aestivum	41	36.0	3.84
834	2015	2015	2015	MALDA		MALDA	5	NEPZ	NORMAL	NOT_HOSTILE	7.7	2	KRL_19	22	Triticum_aestivum	42	28.3	4.08
835	2015	2015	2015	MALDA		MALDA	5	NEPZ	NORMAL	NOT_HOSTILE	7.7	2	DBW_71	9	Triticum_aestivum	43	38.2	4.46
836	2015	2015	2015	MALDA		MALDA	5	NEPZ	NORMAL	NOT_HOSTILE	7.7	2	DPW_621-50	10	Triticum_aestivum	44	35.8	4.24
837	2015	2015	2015	MALDA		MALDA	5	NEPZ	NORMAL	NOT_HOSTILE	7.7	2	HD_2967	15	Triticum_aestivum	45	38.4	5.48
838	2015	2015	2015	MALDA		MALDA	5	NEPZ	NORMAL	NOT_HOSTILE	7.7	2	HI_1563	16	Triticum_aestivum	46	32.9	4.82
839	2015	2015	2015	MALDA		MALDA	5	NEPZ	NORMAL	NOT_HOSTILE	7.7	2	NW_4018	28	Triticum_aestivum	47	35.5	4.38
840	2015	2015	2015	MALDA		MALDA	5	NEPZ	NORMAL	NOT_HOSTILE	7.7	2	KRL_213	24	Triticum_aestivum	48	31.8	5.96
841	2015	2015	2015	MALDA		MALDA	5	NEPZ	NORMAL	NOT_HOSTILE	7.7	2	KHARCHIA_65	20	Triticum_aestivum	49	40.4	3.4
842	2015	2015	2015	MALDA		MALDA	5	NEPZ	NORMAL	NOT_HOSTILE	7.7	2	RW_3684	33	Triticum_aestivum	50	34.4	4.82
843	2015	2015	2015	MALDA		MALDA	5	NEPZ	NORMAL	NOT_HOSTILE	7.7	2	NW_4092	29	Triticum_aestivum	51	40.2	4.74
844	2015	2015	2015	MALDA		MALDA	5	NEPZ	NORMAL	NOT_HOSTILE	7.7	2	GW_322	11	Triticum_aestivum	52	34.5	5.64
845	2015	2015	2015	MALDA		MALDA	5	NEPZ	NORMAL	NOT_HOSTILE	7.7	2	UP_262	34	Triticum_aestivum	53	37.3	5.36
846	2015	2015	2015	MALDA		MALDA	5	NEPZ	NORMAL	NOT_HOSTILE	7.7	2	DBW_51	8	Triticum_aestivum	54	38.4	5.02
847	2015	2015	2015	MALDA		MALDA	5	NEPZ	NORMAL	NOT_HOSTILE	7.7	2	RAJ_4238	32	Triticum_aestivum	55	43.4	2.4
848	2015	2015	2015	MALDA		MALDA	5	NEPZ	NORMAL	NOT_HOSTILE	7.7	2	DBW_17	5	Triticum_aestivum	56	34.1	3.6
849	2015	2015	2015	MALDA		MALDA	5	NEPZ	NORMAL	NOT_HOSTILE	7.7	2	KRL_1-4	21	Triticum_aestivum	57	36.6	4.92
850	2015	2015	2015	MALDA		MALDA	5	NEPZ	NORMAL	NOT_HOSTILE	7.7	2	WH_1105	36	Triticum_aestivum	58	32.3	4.22
851	2015	2015	2015	MALDA		MALDA	5	NEPZ	NORMAL	NOT_HOSTILE	7.7	2	NW_1067	27	Triticum_aestivum	59	35.6	4.24
852	2015	2015	2015	MALDA		MALDA	5	NEPZ	NORMAL	NOT_HOSTILE	7.7	2	DBW_39	6	Triticum_aestivum	60	42.4	5.68
853	2015	2015	2015	MALDA		MALDA	5	NEPZ	NORMAL	NOT_HOSTILE	7.7	2	HD_2009	12	Triticum_aestivum	61	46.0	4.48
854	2015	2015	2015	MALDA		MALDA	5	NEPZ	NORMAL	NOT_HOSTILE	7.7	2	K_0307	19	Triticum_aestivum	62	40.2	4.68
855	2015	2015	2015	MALDA		MALDA	5	NEPZ	NORMAL	NOT_HOSTILE	7.7	2	KRL_3-4	25	Triticum_aestivum	63	19.6	1.44
856	2015	2015	2015	MALDA		MALDA	5	NEPZ	NORMAL	NOT_HOSTILE	7.7	2	PDW_314	30	Triticum_turgidum	64	43.2	5.2
857	2015	2015	2015	MALDA		MALDA	5	NEPZ	NORMAL	NOT_HOSTILE	7.7	2	KRL_210	23	Triticum_aestivum	65	33.6	6.22
858	2015	2015	2015	MALDA		MALDA	5	NEPZ	NORMAL	NOT_HOSTILE	7.7	2	HI_8498	17	Triticum_turgidum	66	47.6	4.38
859	2015	2015	2015	MALDA		MALDA	5	NEPZ	NORMAL	NOT_HOSTILE	7.7	2	DBW_46	7	Triticum_aestivum	67	43.1	3.98
860	2015	2015	2015	MALDA		MALDA	5	NEPZ	NORMAL	NOT_HOSTILE	7.7	2	RAJ_4229	31	Triticum_aestivum	68	33.5	5.64
861	2015	2015	2015	MALDA		MALDA	5	NEPZ	NORMAL	NOT_HOSTILE	7.7	2	CBW_38	2	Triticum_aestivum	69	36.1	6
862	2015	2015	2015	MALDA		MALDA	5	NEPZ	NORMAL	NOT_HOSTILE	7.7	2	DBW_14	3	Triticum_aestivum	70	34.1	5.32
863	2015	2015	2015	MALDA		MALDA	5	NEPZ	NORMAL	NOT_HOSTILE	7.7	2	HD_2932	14	Triticum_aestivum	71	32.2	5.9
864	2015	2015	2015	MALDA		MALDA	5	NEPZ	NORMAL	NOT_HOSTILE	7.7	2	DBW_16	4	Triticum_aestivum	72	37.8	5.28

Samples!	B	Na	Mg	P	S	K	Ca	Ti	Li	Be	Al	V	Cr	Mn	Fe	Co
812	1.346226465	49.53407069	1045.713252	3535.327312	1257.968235	3657.487695	343.3304606	52.57059352	0.004943965	0.000391782	6.995184247	0.005652759	1.074675385	45.41255742	43.15880314	0.080958943
813	1.346226465	49.53407069	1450.519743	4907.145377	2240.046459	5340.470749	434.5016623	52.57059352	0.004943965	0.000391782	6.995184247	0.005652759	1.074675385	49.17841682	57.3066999	0.061773811
814	1.346226465	49.53407069	993.2036552	3528.547674	1548.557687	3908.655552	340.2716294	52.57059352	0.004943965	0.000391782	6.995184247	0.005652759	1.074675385	40.20003887	40.77985453	0.027737159
815	1.346226465	49.53407069	991.5934578	3659.026337	1533.04599	3791.216734	299.4080461	52.57059352	0.004943965	0.000391782	6.995184247	0.005652759	1.074675385	38.95215189	38.53227552	0.027737159
816	1.346226465	49.53407069	1254.51358	4224.248112	1569.898925	4113.410578	259.9045276	52.57059352	0.004943965	0.000391782	6.995184247	0.005652759	1.074675385	46.73674465	42.59414534	0.027737159
817	1.346226465	49.53407069	946.8784551	3514.588355	1303.654748	4202.975908	322.5409683	52.57059352	0.004943965	0.000391782	6.995184247	0.005652759	1.074675385	47.48878032	37.65717398	0.027737159
818	1.346226465	49.53407069	888.7314872	3265.123212	1416.604604	3885.313916	236.3851027	52.57059352	0.004943965	0.000391782	6.995184247	0.005652759	1.074675385	37.33340132	32.6711801	0.027737159
819	1.346226465	49.53407069	1023.380845	3698.20473	1547.428491	4409.623979	296.3819018	52.57059352	0.004943965	0.000391782	6.995184247	0.005652759	1.074675385	37.1414535	43.25159572	0.027737159
820	1.346226465	49.53407069	1103.196813	3969.365532	1377.086741	4707.087026	332.2167155	52.57059352	0.004943965	0.000391782	6.995184247	0.005652759	1.074675385	35.01431897	41.04644061	0.027737159
821	1.346226465	49.53407069	1003.969268	3552.452113	1431.678139	3924.883691	270.4624502	52.57059352	0.004943965	0.000391782	6.995184247	0.005652759	1.074675385	53.75029581	36.61652504	0.027737159
822	1.346226465	49.53407069	1016.512996	3694.332282	1470.049696	4510.673401	360.5419494	52.57059352	0.004943965	0.000391782	6.995184247	0.005652759	1.074675385	36.64535099	36.08600331	0.027737159
823	1.346226465	49.53407069	1005.132003	3516.320833	1305.143329	4087.932697	255.8064377	52.57059352	0.004943965	0.000391782	6.995184247	0.005652759	1.074675385	35.48877687	33.72535742	0.027737159
824	1.346226465	49.53407069	1112.167661	3613.640199	1360.237307	3817.017633	334.8605922	52.57059352	0.004943965	0.000391782	6.995184247	0.005652759	1.074675385	43.58690065	40.8719565	0.027737159
825	1.346226465	49.53407069	1706.466256	4894.178631	2058.730235	3875.059572	392.0023797	52.57059352	0.004943965	0.000391782	6.995184247	0.005652759	1.074675385	62.07790286	60.60906995	0.027737159
826	1.346226465	49.53407069	1113.005311	3639.60547	1719.031939	3945.206142	321.6896342	52.57059352	0.004943965	0.000391782	6.995184247	0.005652759	1.074675385	45.18222348	39.3101157	0.027737159
827	1.346226465	49.53407069	969.8408969	3408.266755	1363.201214	4146.543005	342.1090389	52.57059352	0.004943965	0.000391782	6.995184247	0.005652759	1.074675385	43.01264546	72.28225505	0.027737159
828	1.346226465	49.53407069	1064.7041	3645.432987	1589.750222	3729.318336	238.1851002	52.57059352	0.004943965	0.000391782	6.995184247	0.005652759	1.074675385	40.4348058	40.7128572	0.027737159
829	1.346226465	49.53407069	937.3813421	3081.724393	1234.532801	4012.741973	300.3549973	52.57059352	0.004943965	0.000391782	6.995184247	0.005652759	1.074675385	41.64096735	44.6793504	0.027737159
830	1.346226465	49.53407069	932.512417	3045.42334	1268.089977	3756.146887	228.8907208	52.57059352	0.004943965	0.000391782	6.995184247	0.005652759	1.074675385	34.51632553	44.63809018	0.027737159
831	1.346226465	49.53407069	1205.413247	3782.335303	1786.877943	3753.811242	285.8341638	52.57059352	0.004943965	0.000391782	6.995184247	0.014911021	2.257866016	49.70692626	62.49306012	0.059901705
832	1.346226465	49.53407069	920.2926223	2933.367002	1355.008027	4197.547499	343.3624022	52.57059352	0.004943965	0.000391782	6.995184247	0.005652759	1.074675385	40.14291547	44.32110365	0.096886804
833	1.346226465	49.53407069	1103.04728	3784.000108	1827.285734	3508.197628	284.5005816	52.57059352	0.004943965	0.000391782	6.995184247	0.005652759	1.074675385	52.62845353	51.57017906	0.027737159
834	1.346226465	49.53407069	1054.685556	3509.157061	1551.099792	4470.509534	395.6064794	52.57059352	0.004943965	0.000391782	6.995184247	0.005652759	1.074675385	35.14063865	40.5502323	0.027737159
835	1.346226465	49.53407069	925.7378537	3396.814464	1326.480957	4210.030092	213.4234263	52.57059352	0.004943965	0.000391782	6.995184247	0.005652759	1.074675385	33.76936972	35.62470309	0.027737159
836	1.346226465	49.53407069	943.7193112	3371.463005	1342.144106	4342.219255	273.09379	52.57059352	0.004943965	0.000391782	6.995184247	0.005652759	1.074675385	36.98098257	37.24596392	0.027737159
837	1.346226465	49.53407069	1141.032131	3724.676472	1608.276219	4131.025509	315.5948129	52.57059352	0.004943965	0.000391782	6.995184247	0.005652759	1.074675385	44.20268823	41.79397006	0.027737159
838	1.346226465	49.53407069	988.7199209	3163.524783	1462.994652	3666.487173	349.3047625	52.57059352	0.004943965	0.000391782	6.995184247	0.005652759	1.074675385	41.25421201	45.648407	0.027737159
839	1.346226465	49.53407069	1132.382798	3568.886339	1825.349351	3764.942523	289.3515637	52.57059352	0.004943965	0.000391782	6.995184247	0.005652759	1.074675385	45.70414841	48.45700699	0.027737159
840	1.346226465	49.53407069	835.3889998	3173.441325	1581.532581	3838.02142	272.6574946	52.57059352	0.004943965	0.000391782	6.995184247	0.005652759	1.074675385	39.30696056	45.78499629	0.027737159
841	1.346226465	49.53407069	933.1519179	3124.612554	1143.51429	3883.461735	270.071623	52.57059352	0.004943965	0.000391782	6.995184247	0.01686516		38.2429024	59.04387785	0.058362336
842	1.346226465	49.53407069	912.8596636	3291.037968	1358.641917	4072.596541	301.0146722	52.57059352	0.023212381	0.000391782	6.995184247	0.013096058	1.074675385	44.45282164	37.2435284	0.027737159
843	1.346226465	49.53407069	1197.356267	4324.99612	1825.170285	4158.094922	304.5166884	52.57059352	0.004943965	0.000391782	6.995184247	0.005652759	1.074675385	44.68251392	48.80280914	0.027737159
844	1.346226465	49.53407069	1031.733824	3436.942156	1479.403243	4366.713038	356.3770249	52.57059352	0.004943965	0.000391782	6.995184247	0.005652759	1.074675385	42.22698801	35.5139898	0.027737159
845	1.346226465	49.53407069	1104.307958	3301.002207	1723.211584	3612.867499	298.9882795	52.57059352	0.004943965	0.000391782	6.995184247	0.005652759	1.074675385	42.6951917	44.49212658	0.027737159
846	1.346226465	49.53407069	1113.171683	3992.567355	1687.090645	4021.845599	285.2851484	52.57059352	0.004943965	0.000391782	6.995184247	0.005652759	1.074675385	45.20903567	44.00691636	0.027737159
847	1.346226465	49.53407069	1438.855801	4685.155021	1866.464932	3723.174956	267.9325877	52.57059352	0.004943965	0.000391782	6.995184247	0.005652759	1.074675385	53.64735133	50.6425172	0.027737159
848	1.346226465	49.53407069	991.9968779	3715.179688	1433.397331	4292.111388	258.2139145	52.57059352	0.004943965	0.000391782	6.995184247	0.005652759	1.074675385	40.04934753	36.79086883	0.027737159
849	1.346226465	49.53407069	1132.826303	3494.442613	1531.630602	3827.660194	309.3067231	52.57059352	0.004943965	0.000391782	6.995184247	0.005652759	1.074675385	43.57958581	47.15757621	0.027737159
850	1.346226465	49.53407069	992.6695736	3402.722221	1542.278669	4034.428405	353.0146765	52.57059352	0.011452626	0.000391782	6.995184247	0.005652759	1.074675385	48.22231625	50.66633337	0.027737159
851	1.346226465	49.53407069	1087.577689	3830.800537	1807.496907	4785.787427	384.6794793	52.57059352	0.004943965	0.000391782	6.995184247	0.005652759	1.074675385	44.81210965	52.71560208	0.027737159
852	1.346226465	49.53407069	1122.068222	3829.889801	1529.967682	3969.776333	259.4558865	52.57059352	0.004943965	0.000391782	6.995184247	0.005652759	1.074675385	48.75011618	46.93862022	0.027737159
853	1.346226465	49.53407069	893.7987073	3427.0714	1377.034606	4185.082314	299.6636328	52.57059352	0.004943965	0.000391782	6.995184247	0.005652759	1.074675385	33.79303913	37.57290324	0.027737159
854	1.346226465	49.53407069	1064.50868	3452.091572	1369.282326	4003.704002	275.6331064	52.57059352	0.004943965	0.000391782	6.995184247	0.005652759	1.074675385	46.06300548	42.03182396	0.027737159
855	1.346226465	49.53407069	1436.155376	4339.686274	2097.538488	5151.50104	457.032522	52.57059352	0.004943965	0.000391782	6.995184247	0.005652759	1.074675385	42.59332608	50.14358477	0.062265787
856	1.346226465	49.53407069	874.0455572	3537.548146	1423.936594	4184.28425	299.1708798	52.57059352	0.004943965	0.000391782	6.995184247	0.005652759	1.074675385	31.75325564	40.6815724	0.027737159
857	1.346226465	49.53407069	1039.682448	3529.388403	1696.647176	3698.783393	403.0700534	52.57059352	0.004943965	0.000391782	6.995184247	0.005652759	1.074675385	58.11509695	51.40428104	0.027737159
858	1.346226465	49.53407069	978.2370609	3714.750735	1290.927588	4562.324846	285.1626625	52.57059352	0.004943965	0.000391782	6.995184247	0.005652759	1.074675385	34.52487439	41.91036005	0.027737159
859	1.346226465	49.53407069	1041.601415	3869.09292	1366.633631	4343.120725	294.8471877	52.57059352	0.004943965	0.000391782	6.995184247	0.005652759	1.074675385	45.8026937	51.33890751	0.027737159
860	1.346226465	49.53407069	1007.782199	3803.462638	1407.522975	4614.828194	258.4431783	52.57059352	0.004943965	0.000391782	6.995184247	0.005652759	1.074675385	39.07610336	42.28340524	0.027737159
861	1.346226465	49.53407069	1081.527362	3818.487459	1549.143022	4132.385044	239.8391529	52.57059352	0.004943965	0.000391782	6.995184247	0.005652759	1.074675385	44.16790905	44.3826685	0.027737159
862	1.346226465	49.53407069	1126.335684	3907.351057	1522.932073	3892.531946	346.8115899	52.57059352	0.004943965	0.000391782	6.995184247	0.005652759	1.074675385	45.30448094	44.2936879	0.027737159
863	1.346226465	49.53407069	1041.523171	3731.217077	1652.746927	4096.991292	329.4027817	52.57059352	0.004943965	0.000391782	6.995184247	0.005652759	1.074675385	47.63276892	51.46794748	0.082216573
864	1.346226465	49.53407069	1001.563226	3222.09357	1417.879408	3508.295999	284.9937185	52.57059352	0.004943965	0.000391782	6.995184247	0.005652759	1.074675385	37.586693	40.44129329	0.027737159

Samples!	Ni	Cu	Zn	As	Se	Rb	Sr	Mo	Ag	Cd	Cs	Ba	Tl	Pb	U
812	0.516801721	5.06019453	36.23205674	0.006075947	0.060637731	4.138514665	2.00593359	0.334773253	0.006342015	0.017057123	0.002928171	10.09967369	0.000522746	0.032992059	0.002621754
813	0.516801721	5.21191946	41.5466112	0.009981597	0.072282027	14.00365463	1.608571422	0.209054632	0.006342015	0.064949718	0.012963849	5.470597859	0.000522746	0.032992059	0.002621754
814	0.516801721	3.828720182	33.51314772	0.00610026	0.055740079	8.263007822	1.322528481	0.289323756	0.006342015	0.017057123	0.007536665	4.124941884	0.000522746	0.032992059	0.002621754
815	0.516801721	4.409602732	30.80568762	0.002818625	0.056779157	7.312458783	1.080107955	0.233844368	0.006342015	0.017057123	0.006835655	3.040990948	0.000522746	0.032992059	0.002621754
816	0.516801721	3.891585756	41.31012813	0.002818625	0.078249388	5.728227048	1.347539238	0.344305257	0.006342015	0.041446673	0.003237907	5.051138021	0.000522746	0.032992059	0.002621754
817	0.516801721	4.413010902	30.19339011	0.002818625	0.053603821	3.007693481	1.766548153	0.349148008	0.006342015	0.017057123	0.001398177	6.109181263	0.000522746	0.032992059	0.002621754
818	0.516801721	4.131055252	31.26721182	0.002818625	0.069540665	5.207747319	1.137924539	0.342702922	0.006342015	0.017057123	0.002787477	4.487895639	0.000522746	0.032992059	0.002621754
819	0.516801721	4.64467011	27.69219514	0.012253097	0.041724761	6.619144079	1.390939199	0.225611841	0.006342015	0.040584032	0.006724226	6.54527537	0.000522746	0.032992059	0.002621754
820	0.516801721	4.713637286	39.53300031	0.007508146	0.10641802	8.613023423	1.227749347	0.224752543	0.006342015	0.017057123	0.009088311	4.013985363	0.000522746	0.032992059	0.002621754
821	0.516801721	3.918536185	31.10503321	0.002818625	0.048133951	6.26983659	1.189117284	0.260736131	0.006342015	0.017057123	0.003010866	4.380950546	0.000522746	0.032992059	0.002621754
822	0.516801721	4.335715928	33.17569458	0.005988583	0.056462706	5.680265969	1.747273731	0.21401274	0.006342015	0.038586383	0.004017599	8.56149697	0.000522746	0.032992059	0.002621754
823	0.516801721	4.079849756	33.40406781	0.002818625	0.05631447	2.660607229	1.053599989	0.392501273	0.006342015	0.017057123	0.001835272	3.997797355	0.000522746	0.032992059	0.002621754
824	0.516801721	4.908650988	39.23074015	0.002818625	0.071094312	4.130269429	1.266360436	0.294010374	0.006342015	0.041451182	0.002523472	3.179515814	0.000522746	0.032992059	0.002621754
825	0.516801721	6.512395262	58.08166864	0.00762673	0.086571739	8.248043249	1.727631472	0.389777823	0.006342015	0.017057123	0.008085132	4.579308562	0.000522746	0.032992059	0.002621754
826	0.516801721	4.117371973	38.54870996	0.009818339	0.087340028	7.561118758	1.190830224	0.33643431	0.006342015	0.017057123	0.006484272	4.430943403	0.000522746	0.032992059	0.002621754
827	0.516801721	3.794414065	34.91769056	0.002818625	0.037618986	4.710118114	1.253150652	0.176765587	0.006342015	0.017057123	0.003078597	6.248238347	0.000522746	0.032992059	0.002621754
828	0.516801721	3.948019029	37.32376524	0.002818625	0.051056287	5.390776525	1.182902416	0.236340554	0.006342015	0.042423384	0.002975683	3.322803158	0.000522746	0.032992059	0.002621754
829	1.198820067	5.059188062	30.97809244	0.020932453	0.065444896	2.920475536	1.230926103	0.403707581	0.015481329	0.017057123	0.001349214	5.538089585	0.000522746	0.032992059	0.002621754
830	0.516801721	5.680301567	32.47517502	0.002818625	0.088209648	3.777294147	1.058192921	0.366473929	0.012916753	0.017057123	0.001991731	4.976435171	0.000522746	0.032992059	0.002621754
831	0.516801721	5.5133633	35.17269357	0.027901398	0.058377595	8.176624706	1.53266541	0.379852611	0.015051974	0.049279589	0.007037342	4.228973876	0.000522746	0.10478074	0.002621754
832	0.516801721	4.716514424	25.58066944	0.014463446	0.043642639	7.17594575	1.451879362	0.234554577	0.006342015	0.017057123	0.009089875	5.590544732	0.000522746	0.165284149	0.002621754
833	0.516801721	4.849779565	35.81696089	0.002818625	0.064091253	7.186632867	1.020439431	0.274159265	0.006342015	0.062820186	0.005385341	4.375331098	0.000522746	0.11090005	0.002621754
834	0.516801721	4.842163432	30.83489149	0.002818625	0.062253996	6.82407892	1.890139101	0.184100443	0.006342015	0.038447216	0.005138075	8.227545669	0.000522746	0.032992059	0.002621754
835	0.516801721	4.252638663	26.34730835	0.002818625	0.067676132	2.291376336	0.866403638	0.34875412	0.006342015	0.017057123	0.000992628	3.129057052	0.000522746	0.032992059	0.002621754
836	0.516801721	4.164498385	28.6573703	0.00650176	0.035841256	3.146003783	1.201229155	0.270320391	0.006342015	0.035231373	0.001761832	6.965708481	0.000522746	0.032992059	0.002621754
837	0.516801721	4.305013157	37.64730119	0.04052469	0.06724557	8.447886069	1.241165872	0.314904637	0.006342015	0.017057123	0.007086639	3.348822355	0.000522746	0.032992059	0.002621754
838	0.516801721	5.022110621	29.1535282	0.002818625	0.061270537	6.910210643	1.838407332	0.202819779	0.006342015	0.035562646	0.007048767	8.62356657	0.000522746	0.032992059	0.002621754
839	0.516801721	5.857526212	35.60964631	0.0068478	0.056890881	6.173821142	1.095196875	0.438335929	0.017835366	0.059473495	0.00479658	4.438789125	0.000522746	0.032992059	0.002621754
840	0.516801721	6.109081153	27.6093957	0.010402559	0.061634674	6.653073148	1.219721822	0.422010066	0.021968042	0.017057123	0.005141359	3.081556135	0.000522746	0.032992059	0.002621754
841	2.132792827	5.084372102	34.51755954	0.011440779	0.079354795	3.141761488	1.023379493	0.466965376	0.039977378	0.017057123	0.002699314	3.314802569	0.000522746	0.128063241	0.011896438
842	0.516801721	4.297370186	29.00168535	0.015820101	0.07201486	7.103858496	1.944654974	0.281732881	0.013427139	0.017057123	0.004148698	6.497383924	0.000522746	0.032992059	0.002621754
843	0.516801721	5.075770504	41.46597323	0.020672973	0.049899541	9.673834551	1.037227275	0.377230493	0.006342015	0.017057123	0.010684394	2.617063045	0.000522746	0.032992059	0.002621754
844	0.516801721	4.734189348	29.62724995	0.008718934	0.043063811	9.03852765	1.552046603	0.174767547	0.006342015	0.017057123	0.007668982	4.827995314	0.000522746	0.032992059	0.002621754
845	0.516801721	4.361210672	33.92935524	0.002818625	0.047617234	6.968416068	1.05558421	0.295177906	0.006342015	0.039019588	0.008235155	3.086297743	0.000522746	0.032992059	0.002621754
846	0.516801721	4.452312458	42.41871208	0.03654667	0.07986998	6.24397635	1.095011702	0.354756031	0.006342015	0.017057123	0.005223024	3.427429838	0.000522746	0.032992059	0.002621754
847	0.516801721	5.666286989	52.12277561	0.007010277	0.141240081	5.918299866	1.155914114	0.483056036	0.006342015	0.017057123	0.004485977	3.652316601	0.000522746	0.032992059	0.002621754
848	0.516801721	4.453549891	35.50334462	0.007998302	0.042269842	6.310074106	1.61856098	0.258496941	0.006342015	0.017057123	0.004550213	5.365377579	0.000522746	0.032992059	0.002621754
849	0.516801721	6.236096457	28.61565496	0.007291787	0.038828055	6.490543862	1.279584137	0.320287881	0.017239338	0.05781224	0.009262309	4.148555665	0.000522746	0.032992059	0.002621754
850	0.516801721	5.686898436	28.60496442	0.012067674	0.056534225	11.08503362	1.335757316	0.29030658	0.006342015	0.017057123	0.012797646	3.780592633	0.000522746	0.121728643	0.002621754
851	1.266143337	4.952321453	30.18630581	0.002818625	0.059730962	7.578920107	1.655668411	0.234044103	0.006342015	0.064079715	0.004004151	4.99064129	0.000522746	0.032992059	0.002621754
852	0.516801721	4.244852837	33.1486182	0.002818625	0.05629204	6.600345275	1.119872126	0.262384984	0.006342015	0.055879432	0.003726873	3.237728882	0.000522746	0.032992059	0.002621754
853	0.516801721	3.972466526	24.23936392	0.002818625	0.037208898	3.48480906	1.047483499	0.323084313	0.006342015	0.017057123	0.002785248	2.70348843	0.000522746	0.032992059	0.002621754
854	0.516801721	4.5789183	32.97353724	0.002818625	0.037607954	5.552866298	1.026042231	0.271383639	0.006342015	0.041042258	0.004991125	2.950541552	0.000522746	0.032992059	0.002621754
855	0.516801721	4.421209406	38.503547	0.011819065	0.063072537	18.02526092	1.711793369	0.192183332	0.006342015	0.066332965	0.021695453	4.874812228	0.000522746	0.032992059	0.002621754
856	0.516801721	4.985809964	27.2502812	0.002818625	0.041140216	10.71311703	0.49677157	0.209356901	0.006342015		0.010058869	1.168014209	0.000522746	0.032992059	0.002621754
857	0.516801721	4.39475441	31.39486442	0.002818625	0.049486912	6.881218967	1.759844443	0.179140566	0.006342015	0.017057123	0.005204468	4.011357122	0.000522746	0.032992059	0.002621754
858	0.516801721	5.243822927	29.66133277	0.014345127	0.057927147	4.985609839	0.60443266	0.223730136	0.006342015	0.044374953	0.004683516	2.174011495	0.000522746	0.032992059	0.002621754
859	0.516801721	5.297597081	29.19889986	0.007291787	0.044084633	4.497953561	1.131263021	0.464938416	0.015989984	0.017057123	0.004347188	3.965241973	0.000522746	0.032992059	0.002621754
860	0.516801721	5.084745672	28.45178631	0.002818625	0.035783111	6.664733714	1.121888433	0.311852891	0.013611803	0.041524098	0.006316388	5.032674145	0.000522746	0.032992059	0.002621754
861	0.516801721	4.231083227	31.36262375	0.008059997	0.055176507	8.73363221	1.051171357	0.287903768	0.006342015	0.017057123	0.004801396	3.431249288	0.000522746	0.032992059	0.002621754
862	0.516801721	4.923343428	29.22100567	0.006476555	0.052393529	6.783066216	1.67035663	0.241484396	0.006342015	0.062379362	0.006223686	6.625042932	0.000522746	0.032992059	0.002621754
863	0.516801721	4.039425905	31.55927497	0.002818625	0.050969563	6.792907248	1.100759597	0.222179051	0.006342015	0.017057123	0.004547862	3.497133262	0.000522746	0.032992059	0.002621754
864	0.516801721	4.547143724	32.53688275	0.002818625	0.073651398	4.423078541	0.987388353	0.305214992	0.006342015	0.017057123	0.003842797	4.15105807	0.000522746	0.032992059	0.002621754
